# Gene expression in developing fibres of Upland cotton (*Gossypium hirsutum *L.) was massively altered by domestication

**DOI:** 10.1186/1741-7007-8-139

**Published:** 2010-11-15

**Authors:** Ryan A Rapp, Candace H Haigler, Lex Flagel, Ran H Hovav, Joshua A Udall, Jonathan F Wendel

**Affiliations:** 1 Department of Ecology, Evolution, and Organismal Biology, 251 Bessey Hall, Iowa State University, Ames, IA 50011, USA; 2 Department of Crop Science and Department of Plant Biology, 4405 Williams Hall, Box 7620, North Carolina State University, Raleigh, North Carolina 27695, USA; 3 217 Plant Sciences Institution, Volcani Center, ARO, Bet Dagan, Israel; 4 Department of Biology, Box 90338, Duke University, Durham, North Carolina 27008, USA; 5 295 WIDB, Brigham Young University, Provo, Utah 84602, USA

## Abstract

**Background:**

Understanding the evolutionary genetics of modern crop phenotypes has a dual relevance to evolutionary biology and crop improvement. Modern upland cotton (*Gossypium hirsutum *L.) was developed following thousands of years of artificial selection from a wild form, *G. hirsutum *var. *yucatanense*, which bears a shorter, sparser, layer of single-celled, ovular trichomes ('fibre'). In order to gain an insight into the nature of the developmental genetic transformations that accompanied domestication and crop improvement, we studied the transcriptomes of cotton fibres from wild and domesticated accessions over a developmental time course.

**Results:**

Fibre cells were harvested between 2 and 25 days post-anthesis and encompassed the primary and secondary wall synthesis stages. Using amplified messenger RNA and a custom microarray platform designed to interrogate expression for 40,430 genes, we determined global patterns of expression during fibre development. The fibre transcriptome of domesticated cotton is far more dynamic than that of wild cotton, with over twice as many genes being differentially expressed during development (12,626 versus 5273). Remarkably, a total of 9465 genes were diagnosed as differentially expressed between wild and domesticated fibres when summed across five key developmental time points. Human selection during the initial domestication and subsequent crop improvement has resulted in a biased upregulation of components of the transcriptional network that are important for agronomically advanced fibre, especially in the early stages of development. About 15% of the differentially expressed genes in wild versus domesticated cotton fibre have no homology to the genes in databases.

**Conclusions:**

We show that artificial selection during crop domestication can radically alter the transcriptional developmental network of even a single-celled structure, affecting nearly a quarter of the genes in the genome. Gene expression during fibre development within accessions and expression alteration arising from evolutionary change appears to be 'modular' - complex genic networks have been simultaneously and similarly transformed, in a coordinated fashion, as a consequence of human-mediated selection. These results highlight the complex alteration of the global gene expression machinery that resulted from human selection for a longer, stronger and finer fibre, as well as other aspects of fibre physiology that were not consciously selected. We illustrate how the data can be mined for genes that were unwittingly targeted by aboriginal and/or modern domesticators during crop improvement and/or which potentially control the improved qualities of domesticated cotton fibre.

See Commentary: http://www.biomedcentral.com/1741-7007/8/137

## Background

Domesticated upland cotton, *Gossypium hirsutum *L., provides the largest source of renewable natural textile fibre and also supports the manufacture of diverse consumer products ranging from medical supplies to currency http://www.cotton.org. In addition, by-products from cotton fibre production account for a large percentage of the world's seed oil and protein meal - only bettered by soybean and rapeseed http://www.soystats.com. Upland cotton is now grown commercially around the globe, from the 32nd parallel south in Australia and South America to as far north as the 37th parallel in the USA.

**Figure 1:**
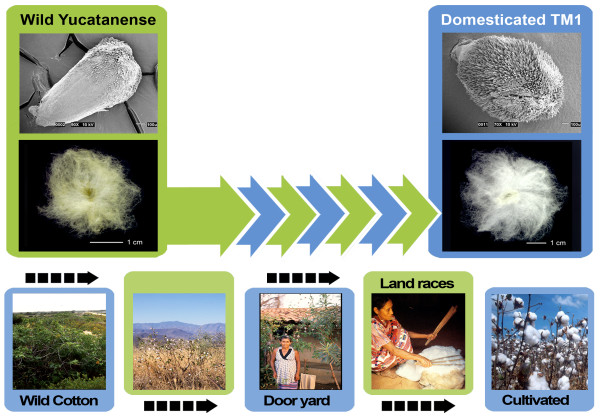
**Domestication of cotton**. Bottom row: domestication by humans. Wild cotton is a sprawling shrub growing in negative association with humans, integrated into native, coastal vegetation (shown bottom left is var. *yucatanense*, image courtesy of J McD Stewart). Domestication has led to a ~7000 year history of the development of perennial dooryard forms, landraces and annualized forms, the latter providing the foundation for modern improvement programmes. Middle row: comparison of a single seed from wild *G. hirsutum *with one from domesticated cotton. Top row: scanning electron microscopy images of cotton ovules at 2 days post anthesis, illustrating differences in the pattern of fibre initials.

As reported elsewhere [1], the genus *Gossypium *appeared approximately 10 million years ago and then diversified into ~45 species with highly variable morphologies, environmental adaptations and life histories. *Gossypium hirsutum *was probably first domesticated ~5000 years ago in the Yucatan peninsula of Mexico [2-4] from plants which were much like the sprawling perennial wild forms (*G. hirsutum *var. *yucatanense*) that are found as integrated components of native vegetation in scattered coastal populations. This progenitor/derivative relationship between the wild plant and the modern crop provides the foundation for our ability to interpret the suite of morphological transitions that led to enhanced fibre quality and yield in domesticated cotton, as well as the concomitant plant growth adaptations required for a crop plant to thrive under agricultural conditions (see Figure 1). For example, Applequist and co-workers [5] showed that wild *G. hirsutum *var. *yucatanense *(called TX2094) has a delayed onset and a shorter period of rapid fibre growth than modern domesticated cotton, *G. hirsutum *cv. TM-1.

Fibres of commercial *Gossypium *species are very elongated and thickened seed epidermal cells that may be spun into yarn. Wild cottons such as *G. hirsutum *var. *yucatanense *have shorter fibres, which, though not spinable, probably represented attractive targets for aboriginal domesticators [6]. Starting from a rangy, perennial shrub with a poorly synchronized fruit set, low yield, photoperiod sensitivity and small seeds that required scarification for germination *in vitro*, human selection over 5 millennia transformed *G. hirsutum *into a high-yielding, annualized, row-crop with a heavy fruit set, photoperiod insensitivity and seeds that germinate readily upon planting. At the same time, fibres became longer, stronger and finer (having less mass per unit length; Figure 1). Myriad semi-domesticated forms and landraces span the wild-to-domesticated continuum, with over 50 cultivated forms and wild races being grouped into seven botanical varieties in the early seminal work [7]. More recent allozyme and restriction fragment length polymorphism (RFLP) analyses [2-4] characterized the genetic diversity within a broad sampling of the over 5000 accessions of *G. hirsutum *maintained by the National Genetic Resources Program http://www.ars-grin.gov/.

Understanding the genetic basis of domesticated phenotypes has dual relevance to evolutionary biology and crop improvement. The molecular underpinnings of morphological and physiological transformations wrought by domestication in other species (such as corn, rice and tomato) have been shown to include diverse molecular phenomena ranging from allelic variants of coding genes to alterations in non-coding DNA remote from the gene of interest [8-16]. For cotton, the initial insights derive from recent studies [17-20] in which comparative gene expression profiling of isolated cotton fibres has been used to suggest, for example, that a key element of the transition from wild to domesticated cotton includes a fine-tuning of the reactive oxygen species (ROS) signalling network which thereby lead to a lengthened period of cell elongation.

As cotton fibre contains a large population of single epidermal cells it provides a facile model for comparative evolutionary genomic analysis [17-19,21,22]. We report the results of a comparative global transcriptomic analysis of fibre development in wild versus domesticated *G. hirsutum*. Fibre cells were harvested at key developmental time points between 2 and 25 days post anthesis (DPA), encompassing the primary and secondary wall synthesis stages. Microarray analysis, followed by clustering to reveal the main patterns of gene expression, [23] was used to categorize and compare the expression levels of 40,430 genes in wild and domesticated cotton. The domestication process resulted in a simultaneous expression alteration of approximately a quarter of all genes in the genome, an extraordinary and massive 'rewiring' of the transcriptome. These results demonstrate the high degree of complexity associated with domestication at the gene expression level, even for a single cell.

## Results

### Determining the time of the onset of secondary wall deposition by microscopic observation

Plant cell walls with a sufficient quantity of organized, crystalline, cellulose exhibit a white birefringence against a black background in a polarizing (POL) microscope. Cotton fibre birefringence only becomes pronounced at the onset of wall thickening, due to an increased percentage of cellulose in the fibre 'winding' cell wall layer (analogous to the S1 layer in wood fibre). In the cotton fibre winding layer, the cellulose microfibrils also adopt an intermediary angle relative to the longitudinal fibre axis (~ 45°) compared to the primary wall (with transverse microfibrils) and the secondary wall (with more steeply angled microfibrils). The presence of cellulose microfibrils oriented at ~45° is detectable with differential interference contrast (DIC) optics and this, together with the increased cell wall birefringence (in POL optics), diagnoses the beginning of fibre wall thickening.

**Figure 2:**
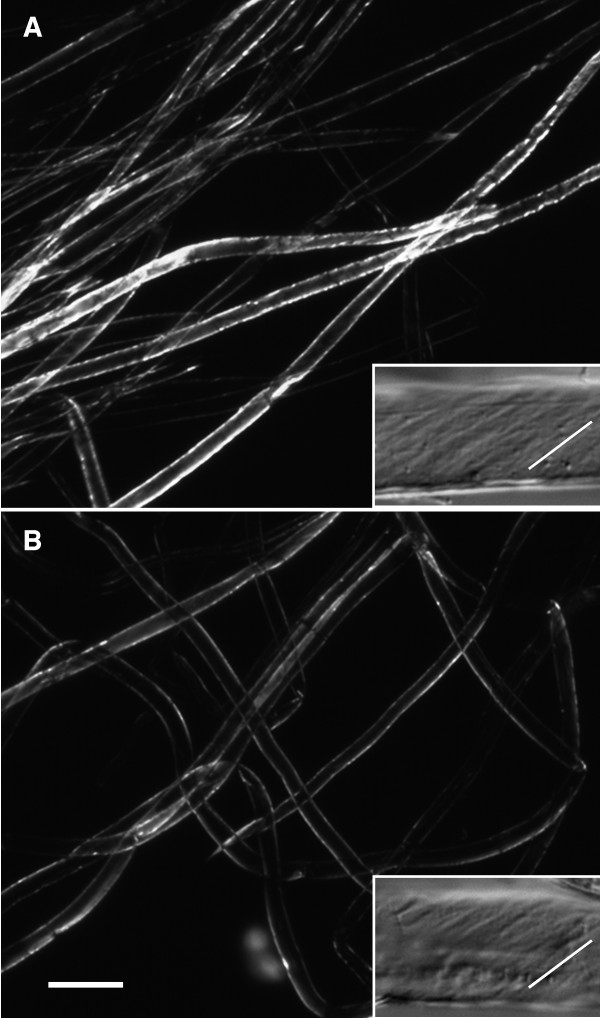
**Representative micrographs of cotton fibre during development, using polarizing (POL) and differential interference contrast (DIC) optics to reveal the onset of wall thickening near 20 days post anthesis in *G. hirsutum*: (A) domesticated TM-1; (B) wild var. *yucatanense***. (A, B) The main micrographs are POL images, showing white birefringence of cellulose against a black background, whereas the insets are DIC images revealing the angle of microfibrils in the winding layer (grey striations, with the angle emphasized by the white line). The magnification bar in the lower left corner of (B) applies to the whole figure and it represents 100 μm for the main POL images and 20 μm for the DIC images in the insets.

At 20 DPA, the walls of both the domesticated TM-1 and the wild *yucatanense *showed white birefringence along the fibre edges in POL optics (Figure 2A and 2B, main micrographs). Fibres of both accessions also had angled cellulose microfibrils as revealed by DIC optics (Figure 2A and 2B, insets). In the Figure 2 insets, the direction of microfibrils relative to the horizontal fibre axis is shown by grey striations and is paralleled by an angled white line. Although the immediately preceding days were not sampled, the micrographs indicate that 20 DPA was near the beginning of the wall thickening because: (a) strong birefringence is only evident along the fibre edges (not over the whole surface, as occurs later when cell walls become thicker, data not shown); and (b) many fibres had not yet begun the wall thickening (appearing to be black or with faint edges in the same view as brighter fibres; Figure 2A and 2B). As indicated by the brighter birefringence in TM-1 fibres (Figure 2A), domesticated cotton was slightly ahead of wild *yucatanense *in the onset of wall thickening. This inference was supported by the rarer detection of angled microfibrils in 20 DPA *yucatanense *fibre (Figure 2B, inset) compared to the frequent observation of this pattern in TM-1 (data not shown): increasing amounts of winding layer cellulose allow this pattern to be more clearly revealed by DIC optics. Prior experience of these optical techniques suggests that the difference in the time of the onset of the secondary wall deposition was less than 1 day between the two accessions (C Haigler, unpublished observations). In general, it is remarkable that the fibre of wild *G. hirsutum *var. *yucatanense *shares such similar timing and morphology of the early wall thickening as modern, domesticated cotton (cv. TM-1), at least when both are grown under relatively cool greenhouse conditions.

### Transcriptomic changes during fibre development within domesticated and wild G. hirsutum

**Figure 3:**
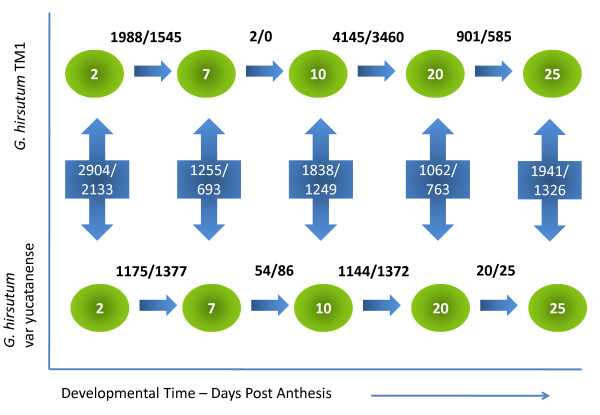
**Genes differentially expressed during fibre development in cotton**. Top row: domesticated TM-1. Bottom row: wild var. *yucatanense*. Green ellipses represent time points of RNA collection, from 2 to 25 days post anthesis (DPA). Arrows represent contrasts used in the statistical model. Numbers above or inside the arrows are the number of genes differentially diagnosed for the specified contrast. For example, for the two stages 2 and 7 DPA within domesticated *G. hirsutum*, 1988 genes were up-regulated at 2 DPA, whereas1545 were more highly expressed at 7 DPA. Contrasts are also shown between wild and domesticated *G. hirsutum*; for example between TM-1 and *yucatanense *at 2 DPA, 2904 genes were more highly expressed in the domesticated form, while for the same contrast 2133 are up-regulated in the wild cotton.

We explored the global transcriptional variation of 40,430 genes over a developmental time course of fibre differentiation (2, 7, 10, 20 and 25 DPA) in *G. hirsutum *cv. TM-1 (domesticated) versus *var. yucatanense *(wild). Figure 3 shows the number of unigenes up- and down-regulated at each stage. Overall, domesticated TM-1 displayed a much higher level of transcriptional variation between the sampled time points than did the wild *yucatanense *accession. When all the developmental transitions were included, 12,626 or 5273 genes experienced significant up- or down-regulation in domesticated or wild cotton, respectively (Figure 3). Between 2 and 7 DPA, 8.7% of assayed genes in TM-1 were differentially expressed [3533 unigenes; *P*-value ≤ 0.05; false discovery rate (FDR) ≤ 0.05], compared to 6.3% (2552 unigenes) in *yucatanense*. Notably, there was little change in the transcriptome between 7 and 10 DPA in TM-1, a period of active fibre elongation; only two unigenes were differentially expressed in TM-1, whereas 140 unigenes (0.35% of the total) had altered expression in *yucatanense*. This difference may relate to differences in the fibre elongation curves between 7 and 16 DPA; TM-1 was entering a sustained period of high rate elongation at 7 DPA whereas *yucatanense *elongation during this period is slower [5]. Far more genes showed altered expression between 10 DPA (during primary wall synthesis) and 20 DPA (when secondary wall thickening was beginning) in both genotypes (Figure 2) [24,25]. During this period, 18.8% (7575) and 6.2% (2516) of the unigenes were differentially expressed in TM-1 and in *yucatanense*, respectively. As secondary wall deposition continued between 20 to 25 DPA, only 1486 (3.7%) or 45 (0.1%) unigenes changed expression in TM-1 or *yucatanense*, respectively.

### Comparison of domesticated to wild *G. hirsutum*

In order to reveal the transcriptomic changes associated with cotton domestication, we compared the gene expression of TM-1 and *yucatanense *at each time point (2, 7, 10, 20, 25 DPA; Figure 3; Additional File 1: Table S1). These data reveal several quantitative perspectives on the pace and direction of gene expression evolution. First, at all time points more genes were up-regulated in TM-1 than in *yucatanense*, implying that human selection pressure led to biased upregulation of components of the transcriptional network that are important for agronomically advanced fibre development. Second, the greatest number of differentially expressed genes between the two accessions occurs early in development (2 DPA) when 12.46% (5037 unigenes) were differentially expressed between the two accessions. This observation implies that the developmental trajectories altered by human selection during domestication and subsequent crop improvement programmes operated strongly, though not exclusively, early in trichome initiation and primary wall synthesis. Third, although there is considerable variation across the fibre developmental profile, thousands of genes are differentially expressed at all stages: by 7 DPA, the fraction of differentially expressed genes is 4.8% of all genes assayed (1948 unigenes), whereas at 10 DPA, differential expression between the two accessions climbs to 7.6% (3078 unigenes), which correlates with the period in TM-1 during which rapid elongation begins [5]. By 20 DPA, the percentage of differentially expressed genes is 4.5% (1825 unigenes), an observation consistent with the shift to secondary wall deposition in both genotypes (Figure 2) and with our earlier study where we showed that the 10-20 DPA transition in TM-1 is associated with the up-regulation of the cotton homologs of many genes known to be essential for secondary wall cellulose synthesis in the xylem and/or interfascicular fibres of *Arabidopsis *and other species [26]. Inspection of the present data revealed that the orthologs of many of these genes are also up-regulated at 20 DPA versus 10 DPA in *yucatanense*. Finally, at 25 DPA, differential expression rises to 8.0% (3267 unigenes), an observation that may, in part, be explained by the fact that more genes in TM-1 showed changed expression between 20 and 25 DPA than in *yucatanense*.

### Gene ontology (GO) categories associated with differentially expressed genes, both within and between wild and modern cotton

In order to explore the functional associations of alterations in gene expression we compiled differentially expressed genes for various comparisons of the array data into GO categories and used Fisher's exact test to test for enrichment of GO terms. These analyses implicated numerous aspects of cellular activities that were differentially represented: (a) as the fibre of each accession progressed through development, 2 to 7 DPA, 10 to 20 DPA and 20 to 25 DPA; and (b) when wild and domesticated cotton fibre was compared at each DPA, from near initiation through secondary wall synthesis. At each time point Fisher's exact test reveals over 100 GO terms are enriched in the contrast between wild and domesticated fibre (Additional File 2: Table S2). Some of these differences are discussed below.

### Massive alteration of gene expression accompanies domestication

**Figure 4:**
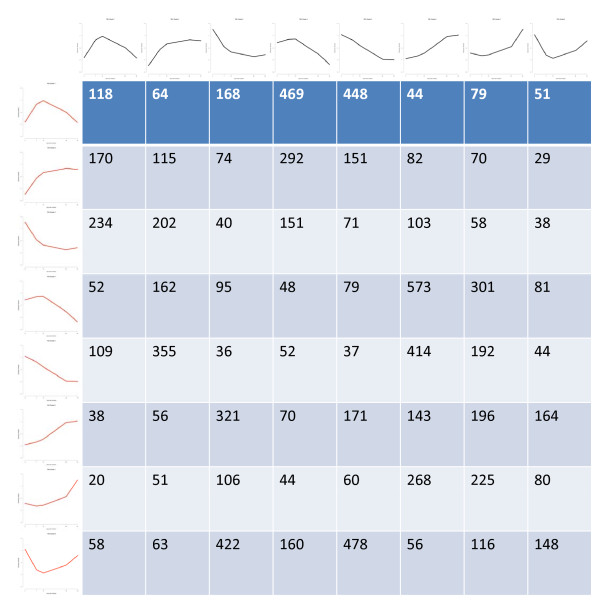
**Transition matrix of expression pattern changes associated with domestication**. The eight magnitude-independent expression patterns recovered by partitioning around medoids clustering found among differentially expressed genes in *yucatanense *are shown in red down the left side, with the same patterns found in domesticated TM-1 shown across the top in black. Lines represent the mean expression at each time point among all genes exhibiting each pattern. The blocks of the matrix contain the number of genes switching expression pattern during the transformation of wild into domesticated cotton. The diagonal represents genes that did not change expression pattern between TM-1 and *yucatanense*, but which experience a statistically significant shift in magnitude at one or more time points.

Across the developmental stages studied, a total of 9465 unigenes were differentially expressed between wild and domesticated cotton fibre. In order to discern the multivariate patterns of gene expression change accompanying the domestication process, gene expression patterns in wild cotton were clustered using partitioning around medoids (PAM) [23], followed by a reassessment of the resulting clustering patterns in domesticated cotton. A transition matrix was constructed in order to show the shifts of differentially expressed genes between patterns in the two accessions. The purpose was to conceptualize, in a multigenic sense, the effects of human selection and crop improvement on transforming ancestral (wild) networks of gene expression (Figure 4, column 1) into their domesticated counterparts (Figure 4, row 1). This analysis provides a quantitative visual depiction of the impact of domestication on gene expression profiles. Additional File 3: Table S3 provides lists of cotton genes in each block of the transition matrix in Figure 4, along with *Arabidopsis *homologs [basic local alignment search tool (BLAST) *e*-value ≤ 1*e*-3] and GO annotations.

As shown in Figure 4, domestication and crop enhancement over 5000 years has resulted in variable numbers of genes in eight expression profiles in wild cotton becoming distributed in domesticated cotton into the expression patterns defined by PAM clustering. However, the redistribution was uneven. For example, 1441 genes experience their lowest expression levels in wild *yucatanense *early in development (2 DPA) and peak in expression intensity by 10 DPA (Figure 4, row 1); when these genes are examined in the domesticated accession TM-1 917 (63.6%) were up-regulated at 2 and 7 DPA (Figure 4, row 1, columns 4 and 5), in contrast to six other expression possibilities. Similarly, genes in wild cotton that have low expression early and increasing expression during development (Figure 4, row 6) are radically altered by domestication in such a way that over half of these same genes in modern cultivated cotton are highly up-regulated early in development (Figure 4, columns 3, 5 and 8). The reciprocal case also appears to be true (Figure 4, row 5) where, in the wild state, the genes are expressed at a high level early and decline through development. Most genes with the high early expression pattern in the wild *yucatanense *experience down-regulation in the domesticated TM-1 (Figure 4, row 5, columns 2 and 6). In summary, genes that undergo expression alteration between wild and domesticated fibre do not do so randomly but, instead, they appear to have been shaped by human selection in a correlated fashion, forming clusters of genes displaying one to several of the possible expression patterns.

In order to aid the functional interpretation of these changes, the genes within blocks of the transition matrix (Figure 4) were assigned GO terms when possible and these were then analysed for significant enrichment of GO terms within blocks. Additional File 4: Table S4 shows lists of enriched GO terms, the corresponding cotton genes and *Arabidopsis *homologs (BLAST *e*-value ≤ 1*e*-10) for certain blocks of the transition matrix, where block IDs are defined by their column, row designators. These files provide a rich data set that can be mined in many different ways for functional implications (see the discussion below).

## Discussion

### Cotton domestication involved transcriptional alteration of thousands of genes

Considerable effort has been made to understand the genetic basis of phenotypes selected from wild populations during the domestication process. Quantitative trait locus (QTL) studies have been particularly fruitful, leading to insights into the mutations responsible for favourable phenotypes in a number of crops [9-11,13,15,16,27]. In cotton, none of the mutations responsible for the morphological transformations between wild and domesticated fibre phenotypes are known and it is equally unclear how many genes govern this phenotypic change. We focused not on the causative lesions *per se *but on the myriad downstream changes in gene expression associated with domestication. We compared patterns of global gene expression from wild and domesticated *G. hirsutum *across five developmental time points representing the key phases of fibre development encompassing initiation, elongation via primary cell wall synthesis and secondary wall synthesis.

Our results show that, at any given time point, more than 1500 genes, or about 3.8% of those assayed (Figure 2) are differentially expressed between wild and domesticated *G. hirsutum*, showing the genomic scale of gene expression change accompanying an altered developmental allometry within a single cell (increased fibre length in domesticated TM-1). The only previously described differences between the fibre of wild and domesticated *G. hirsutum *were in the patterns of initiation on the ovule, the timing of rapid elongation and mature length [5]. Our data show that these and other phenotypic differences involved differential expression of more than 9000 unigenes when summed across five stages of fibre development ranging from 2 - 25 DPA, a surprising number given that only one cell type from two accessions of the same species was analysed. Some perspective on this observation is offered by other plant systems, where comparative expression profiling of whole organs from different cultivars [28], races [29] and species [30-32] has revealed levels of differential expression similar in magnitude to those reported here. In addition, the large number of differentially expressed genes we observed may reflect the high sensitivity and repeatability of the microarray methodology employed, where small technical variances generated high power for the detection of differential expression. As a result, many of the differentially expressed genes in cotton showed only modest magnitudes of changes; for example, 78% and 38% of the differentially expressed genes showed a >1.5-fold and twofold difference, respectively, between wild and domesticated cotton (Additional File 2: Table S2). Nonetheless, the large numbers of differentially expressed genes highlight the complex alteration of global gene expression machinery that resulted from human selection for a longer, stronger and finer fibre, as well as other aspects of fibre physiology that were not consciously selected. Although we identify thousands of gene expression changes concomitant with domestication, it is likely that these changes result from a much smaller number of, as yet, unidentified genetic mutations. If one assumes that domestication involves a modest number of mutations, it seems reasonable to conclude that these mutations have had far reaching, though often relatively small, effects on multiple gene expression networks.

One caveat to our study is that we included only one wild and one domesticated accession of *G. hirsutum *and, therefore, some of the expression changes revealed might reflect the choice of accession rather than the domestication process *per se*. We note, however, that domesticated *G. hirsutum *has a remarkably narrow gene pool, having experienced a severe genetic bottleneck accompanying domestication and crop improvement [2,4]. The resulting high genetic identity among modern varieties suggests that they will show patterns similar to those revealed here and, therefore, that the overall quantitative picture will remain unchanged. Wild accessions are considerably more variable, but truly wild accessions (such as the *yucatanense *accession studied here) also share a high morphological and genetic identity. Ongoing studies employing a broader sampling of wild and cultivated accessions are expected to shed light on the veracity of our quantitative results and, more importantly, will provide additional clues about the specific targets of human selection.

In addition to revealing the power of human selection to cause large-scale shifts in gene expression patterns, these data facilitate the prediction of processes that underlie important phenotypic changes in the fibre of domesticated cotton. Resources that aid these interpretations include Additional File 1: Table S1, which includes lists of genes differentially expressed between TM-1 and *yucatanense *for each time point examined, and Additional Files 3 and 4: Tables S3 and S4, which show, respectively, genes corresponding to each block of the wild-to-domesticated transition matrix and GO categories that could be identified as statistically enriched within certain blocks of the transition matrix. Examples of how these data are useful for making functional interpretations and predictions are provided below.

### Functional analysis of gene expression differences in wild and domesticated *G. hirsutum*

A powerful framework for interpreting the genomic scale data presented here is provided by the ancestor-descendant perspective of domestication combined with an understanding of the homology of fibre development in wild and domesticated *G. hirsutum*. As shown previously [5], scanning electron microscopy showed that both accessions initiate fibre morphogenesis on the day of anthesis, as indicated by the bulging of selected epidermal cells above the ovule surface. In both modern TM-1 and the wild *yucatanense*, the polar growth profile that is characteristic of cotton fibre starts over the next 2 days, although the fibre distribution on the ovule occurs mainly on the chalazal end in wild *yucatanense *but is more evenly distributed in the domesticated TM-1 (Figure 1). Fibre growth curves showed that rapid fibre elongation began about 10 DPA in domesticated TM-1, whereas this phase was delayed until about 15 DPA in wild *yucatanense*. Fibre elongation also ended earlier in *yucatanense *(~20 DPA), whereas it persisted several days longer in TM-1 [5]. Despite these differences in elongation, both accessions begin secondary cell wall thickening at about 20 DPA (Figure 2).

In light of the above, it is notable that, in the present study, the highest percentage of differentially expressed genes (12.46%) between wild and domesticated cotton occurred at 2 DPA (Figure 3). This suggests that the morphological differences that are apparent at this early stage, though somewhat subtle, are accompanied and/or generated by a fairly radical alteration in the cellular transcriptional programme. Interestingly, at 20 and 25 DPA, during the onset and continuation of secondary wall synthesis, 4.5 - 8.0% differentially expressed genes continued to be observed between accessions (Figure 3). Perhaps the more canalized later fibre development reflects, in part, the recently discovered homology [26] between cotton fibre and xylem for genes involved in secondary wall synthesis. Xylem evolved at the base of the land plant lineage and its differentiation programme has been highly conserved for that purpose [33-35] and partly co-opted for cotton fibre wall thickening.

Many of the genes that are differentially expressed in domesticated and wild cotton are 'cotton-specific', as inferred from the lack of significant sequence similarity with other nucleic acid and protein sequences in the National Center for Biotechnology Information database. For example, compared to *yucatanense*, numerous genes which are among the top 25 up-regulated genes at all DPA in TM-1 have no annotation. Indeed, 1000 genes, or about a sixth of those that were differentially regulated between the accessions, had no homology-based annotation after BLAST analysis (data not shown). This highlights the current limited understanding of the mechanistic controls of cellular differentiation and growth and, especially, of specialized cell types such as cotton fibre.

Notwithstanding these limitations, many genes can be, at least tentatively, annotated after BLAST analysis in order to identify their closest homolog in the model plant *Arabidopsis *or other species. This allows the results of our experiments and analyses (Additional Files 1, 3 and 4: Tables S1, S3 and S4) to be mined in order to gain an insight into potential controls of differences in fibre growth between wild and domesticated cotton. For example, many genes known to be involved in cytoskeletal function [36,37] were revealed to be differentially regulated between wild and domesticated cotton. The profilin family of proteins are low molecular weight actin-binding monomers implicated in reorganizing actin filaments during growth [38,39] and are known to play a critical role in the formation of actin microfilaments during cotton fibre development [40,41]. A cotton homolog of *Arabidopsis *PROFILIN5 (AT2G19770) is up-regulated over 27-fold at 7-20 DPA in domesticated TM-1, suggesting selection of this aspect of the cytoskeletal developmental network. Similarly, we detected differential expression of genes encoding the two 55 kD subunits of the microtubule, β-tubulin (TUA) and β-tubulin (TUB) [42]. In our data various tubulin isoforms are over-expressed from 2-20 DPA of fibre development and these are over-expressed in TM-1 from 1.5- to sixfold over the levels in wild cotton, consistent with cotton fibre differentiation requiring dynamic cytoskeletal activity that can also impact upon fibre quality.

We also sought to understand the differences that could account for differences in the elongation rates of wild and domesticated cotton fibre after 10 DPA. Fibre elongation depends on the rapid synthesis of primary cell walls, which have substantial amounts of xyloglucan and pectin surrounding cellulose microfibrils [43]. The xyloglucan is directly associated with the cellulose by connections that can be broken and remade during cell growth by a family of cell wall enzymes called xyloglucan endotransglycosylase/hydrolases (XTH). XTH enzymes have two potential activities - degrading xyloglucan [via xyloglucan endohydrolase, xyloglucan endohydrolases (XEH), activity] and splitting the xyloglucan polymer and reconnecting the end to another xyloglucan molecule [via xyloglucan endotransglucosylase, xyloglucan endotransglycosylase (XET), activity] [44]. Several genes encoding XTH enzymes are strongly up-regulated in the microarray data at 2, 7 and 10 DPA in domesticated cotton fibre but not in wild cotton fibre. These genes include the cotton homologs of At5g65730 (AtXTH6), At4g37800 (AtXTH7) and At5g57560 (AtXTH22). Consistent with these results, quantitative reverse transcription polymerase chain reaction (RT-PCR) showed that the cotton homologs of AtXTH6 and AtXTH7 had peak expression during elongation in fibre of domesticated cotton [45,46].

*Arabidopsis *XTH genes are responsive to a wide variety of stimuli, consistent with roles in plant cell wall remodelling and growth regulation. In particular, AtXTH22 acts down-stream of CYP72C1, a cytochrome P(450) monooxygenase that is proposed to down-regulate brassinolide concentration and thereby reduce the elongation of hypocotyls, petioles, siliques and seeds. Dwarfing of *Arabidopsis *was caused by the activation or over-expression of the CYP72C1 gene, and over-expression caused down-regulation of *AtXTH22 *transcription [47]. CYP72C1 and the related protein CYP734A1 (AT2G26710) both inactivate brassinosteroids and block plant cell elongation [48]. Cotton homologs of CYP734A1 are down-regulated in TM-1 compared to *yucatanense *at 2 and 7 DPA and this could promote earlier fast elongation in domesticated cotton fibre through enhanced brassinosteroid activity. Supporting this possibility, fibre initiation, early elongation and expression of an *XTH *gene in fibre of cultured cotton ovules are promoted by brassinolide [49]. At 10, 20 and 25 DPA, different cotton homologs of CYP734A1 are mostly down-regulated but one is up-regulated, providing evidence that cotton has multiple CYP734A1-type genes that could facilitate the complex regulation of brassinolide responses by mechanisms such as those postulated for *Arabidopsis *[48]. We note that six cotton unigenes homologous to At2g26710 are found in six different blocks of the transition matrix (Figure 4: column 2, rows 2 and 8; column 4, row 7; column 5, rows 1 and 6; and column 7, row 5), consistent with differential selection during domestication acting on different members of the CYP734A1 gene family.

Other XTH genes, the cotton homologs of At4g25810 (AtXTH23), At1g14720 (AtXTH28) and At3g44990 (AtXTH31) are shown to play an important role in controlling early elongation in fibre by: (a) falling within a block of the transition matrix (Figure 4: column 4, row 1) representing 469 genes with earlier up-regulation in TM-1 as compared to *yucatanense*; and (b) having lower expression at 20 and 25 DPA than at 10 DPA. The *Arabidopsis *homolog of one of these, AtXTH28, helps to control elongation in siliques and stamens in a developmentally nuanced manner [50]. Similarly, leaf cell expansion and *AtXTH31 *expression were down-regulated in a *siz1 *mutant that led to salicylic acid accumulation, leading to the proposal that AtXTH31 was a positive effector of cell expansion [51].

The cotton homologs of AtXTH6, AtXTH23, AtXTH28 and AtXTH31 contributed to the enrichment of the GO term, 'cellular component: cell wall' in column 4, row 1 of the transition matrix (Figure 4, Supplemental Table S4). This same block also contains numerous cotton genes with homology to 27 *Arabidopsis *genes with the GO annotation of 'molecular function: oxidoreductase'. Given the previous evidence that modulation of cellular redox status has been important during both cotton fibre evolution and improvement [17,19,20], the biological relevance of the transition matrix and the analysis of enriched GO annotations within it is notable. Several genes deserve to be highlighted. The *Arabidopsis *aldehyde dehydrogenase (ALDH; At4g36250) gene, for example, encodes an enzyme that is important for the detoxification of aldehydes, which are generated during the metabolism of carbohydrates, amino acids and lipids and are chemically reactive and may become toxic at certain concentrations [52]. Over-expression of different aldehyde dehydrogenase genes in *Arabidopsis *confers protection against lipid peroxidation and oxidative stress [53]. Two other important genes are ascorbate peroxidase 3 (APX3; At4g35000 from *Arabidopsis*), which encode peroxisomal membrane-bound antioxidants and are part of the key family involved in cellular H_2_O_2 _metabolism [54,55] and a homolog of glutathione transferase 8 (At1g78380), part of a multi-functional enzyme family that plays a role in the protection of tissues against oxidative damage [56].

By analogy to the foregoing examples, it is possible to examine the genes and enriched GO categories of any block in the transition matrix for functional clues to the genes and physiology involved in cotton fibre development and domestication. (Figure 4: Column 2, row 5, for example, shows a change in expression pattern that could support the prolonged elongation in domesticated cotton via changes in the sucrose transport and cellular redox status. Cotton genes with enriched GO annotations related to sucrose or other sugar transport are homologous to At1g09960 (AtSUT4), At1g71880 (AtSUC1) and At3g19930 (AtSTP4). AtSUT4 is a sucrose transporter localized in the vacuolar membrane of *Arabidopsis *leaf mesophyll cells, whereas AtSUC1 is localized to the plasma membrane [57]. Hoth *et al. *[58] reviews additional data showing that AtSUC1 is a plasma membrane sucrose transporter mainly expressed in pollen, roots and trichomes. However, closely related SUT/SUC proteins in different species can localize to different membranes [57] and, indeed, they may change their location in order to drive particular cellular processes; direct testing of cellular location would therefore be required for the cotton fibre homologs. In any case, these genes encode sucrose transporters that provide the capacity for prolonged turgor-driven elongation [59] in the fibre of domesticated TM-1. AtSTP4 is a stress-regulated plasma membrane monosaccharide transporter. In *Arabidopsis*, it is normally expressed in sink organs, co-regulated with cell wall invertase during powdery mildew infection that causes glucose uptake into host tissues [60] and is quickly up-regulated by ozone along with an oxidative burst [61]. Since the relevant cellular mechanisms linking these phenomena are not fully clarified, the prolonged expression of the cotton homolog of AtSTP4 could sustain fibre elongation in domesticated TM-1 by regulating carbon partitioning and/or by moderating cellular redox status. This indicates the need for a careful analysis of gene function underlying GO annotations in formulating functional hypotheses for cotton genes. In enriched GO annotations related to redox status in Figure 4 column 2, row 5 of the transition matrix, the cotton genes are homologous to At5g23270 (*AtFER1*). As reviewed recently [62], wide expression of *AtFER1 *in *Arabidopsis *supports the regulation of iron concentration and the moderation of ROS levels in response to stress. The levels of ROS detoxifying enzyme activities are also linked into this regulatory circuit [63]. Among four *FER *genes in *Arabidopsis*, AtFER1 expression alone responds positively to H_2_O_2 _[62]. We predict similar functions for the cotton homologs of AtFER1 as a particular means of moderating ROS levels to support continued fibre elongation after H_2_O_2_-stimulated secondary wall deposition has begun [64].

Further emphasizing the importance of sucrose in cotton fibre development, the block Figure 4 column 6, row 4 in the transition matrix shows genes that were highly expressed in wild cotton fibre early in elongation, but whose expression level falls at 20 and 25 DPA. In contrast, these genes are strongly up-regulated in TM-1 at 20 and 25 DPA. Several enriched GO terms relate to sucrose and are associated with cotton sucrose synthase genes - homologs of AtSUS1, SUS3 and SUS5. Despite its name, sucrose synthase (SUS) most commonly degrades sucrose to release uridine diphosphate glucose (UDP)-glucose and fructose in heterotrophic cells [65]. This enzyme plays a key role in cotton fibre development through: (a) generating hexoses to help build the high turgor and/or promote primary wall synthesis required for cotton fibre initiation and elongation [59]; and (b) supplying UDP-glucose to secondary wall CESA proteins [66,67]. At 2, 7 and 10 DPA, the expression level of SUS genes did not vary significantly between TM-1 and wild cotton, which suggests that the role of SUS during primary wall synthesis was fixed early in cotton evolution and that the fast elongation beginning selectively in TM-1 at 10 DPA did not depend on transcriptional control of SUS. However, further experiments would be required in order to determine whether up-regulation of SUS in general, or particular isoforms, later in the development of TM-1 fibre contributed to prolonged elongation or more energetically efficient secondary wall cellulose synthesis or both. Research in cotton could be particularly valuable because *Arabidopsis *research has not revealed distinct cellular roles for SUS isoforms under normal growth conditions [68].

Finally, we provide two examples of how these data can be predictive of, as yet unknown, aspects of fibre development and/or evolution. As characterized in *Arabidopsis*, the highly conserved plant-specific protein impaired sucrose induction1 (ISI1, AT4G27750) is sugar inducible and regulates utilization of sugars for growth. The *isi1 *mutants display elevated chlorophyll levels and depleted starch, suggesting the inefficient use of carbohydrate resources and perturbation of sugar-responsive gene expression [69]. Domesticated TM-1 cotton fibre had higher transcript levels of *ISI1 *homologs at all five time points, compared to wild *yucatanense*, but the five cotton homologs are present in different blocks of the transition matrix. Pending further work, we speculate that finely controlled regulatory shifts for cotton fibre *ISI1 *homologs played an important role in optimizing sugar usage in order to support the development of the fibre quality attributes that humans found most useful. Another example concerns the cotton homolog of At5g54160 (AtCOMT1), which is a strongly down-regulated gene in TM-1 compared to *yucatanense *at all DPA tested in the microarray experiments. Recent data show that the encoded *Arabidopsis *protein can methylate caffeic acid *in vitro*, a process that is associated with the formation of lignin monomers. However, the lignin subunit composition and molecular structure were changed in the *Arabidopsis Atomt1 *mutant, not the quantity of lignin [70]. Although fibres of some cotton cultivars contain up to 1% phenolics [71], any minor true lignin component of the fibre secondary wall has not so far been characterized. This, together with the down-regulation of the cotton homolog of *AtCOMT1 *at all stages of fibre development, suggests that modulation of phenolic molecular structure was important for cotton fibre domestication in, as yet, undefined ways. This and many other aspects of the data could be a target for further experimentation.

## Conclusions

One implication of the present study is that artificial selection during domestication, and by extension the evolutionary process in general, may be manifested as an extraordinarily complex process at the level of gene expression, even in systems entailing the morphological transformation of a single celled structure. We demonstrate altered expression patterns for more than 9000 genes associated with the domestication of cotton fibre, without any knowledge of the causative genetic lesions. However, the large numbers of differentially expressed genes would seem to indicate that the effects of the lesions accompanying domestication have had multiple cascading downstream effects on the machinery of trichome initiation and subsequent primary and secondary wall synthesis.

It is notable that transcriptional alterations among the ~9000 significantly differentially regulated genes are not random with respect to developmental pattern of expression. Instead, and as illustrated in Figure 4, gene expression and gene expression alteration appears to be 'modular', in the sense that large, complex networks of genes seem to be simultaneously and similarly affected by selection under domestication. Other genes have retained a similar pattern of expression, but show either up- or down-regulation in domesticated versus wild cotton. In addition, some gene families did not change either their expression levels or patterns. One example is the cellulose synthase (CESA) gene family. These genes have highly conserved roles in primary and secondary wall synthesis and the timing of these two processes did not change as the wild-to-domesticated fibre transformation occurred.

Insights into the underlying mutational basis of the domestication process and the mechanisms of the action of the downstream genes may be derived from future experiments involving comparisons of near-isogenic introgression lines or other QTL-based methods of gene discovery, as well as the use of transgenic or virus-induced-gene-silencing technology [72] in order to manipulate the expression of putative regulators of the changed patterns in gene expression identified here. Such insight will lead to an enhanced appreciation of how the evolutionary process perturbs nodes of connectivity within gene expression organization, rewriting the timing and tempo of expression that ultimately give rises to novel and/or optimized phenotypes. To the extent that such progress is achieved, insight also will be gained into the basic biology and transcriptional network machinery of fibre biology and development. These insights will, in turn, suggest strategies for targeted genetic changes that may further improve the industrial usefulness of renewable cotton fibre.

## Methods

### Plant material and tissue collection

For elite modern domesticated cotton we selected the genetic and cytogenetic standard, Texas Marker Stock 1 (TM-1). For wild *G. hirsutum*, we used an accession of var. *yucatanense *(USDA GRIN accession PI 501501, also called Tx2094, collected by J McD Stewart), shown in Figure 1 and identified by RFLP analysis [2] and morphological evidence as an excellent exemplar of truly wild (as opposed to feral) *G. hirsutum*. We cold-treated *yucatanense *seeds for 1 week at 4°C and gently scarified the seed coat to break dormancy. After scarification, seeds of TM-1 and *yucatanense *were planted in a sterilized potting mix in the Iowa State University Horticultural Greenhouses. Plants were watered daily, fertilized twice weekly and kept at ambient air temperatures above 20°C. Three biological replicates were grown, with plants reaching reproductive maturity at 3-5 months; wild plants were short-day treated to induce flowering. Flowers were tagged on the day of anthesis and developing bolls were harvested at 2, 7, 10, 20 and 25 DPA. Bolls were dissected immediately after harvest and ovules were flash-frozen in liquid nitrogen and subsequently stored at -80°C. In order to ensure that the progression of fibre development aligned with previously published work [5], and in order to determine the time of onset of fibre wall thickening, fibres were analysed using polarized light microscopy (see below and Figure 2).

### Microscopic analysis

Seeds with attached fibre were removed from bolls at 2, 7, 10, 15 and 20 DPA for *G. hirsutum *cv. TM-1 or 2, 7, 10, 20 and 30 DPA for *G. hirsutum *var. *yucatanense*. Samples of fibre from three seeds at each sampling point were mounted in water and inspected for birefringence in a dedicated POL microscope with rotating stage. Samples with positive birefringence were examined for the presence of angled microfibrils using DIC optics (Olympus BH-2 light microscope platforms; Olympus America Inc, PA, USA). Micrographs were taken with a Q-5 digital camera (QImaging, BC, Canada). For comparison of birefringence intensity in POL images, all fibre samples were viewed at the same angle relative to the optical axis - the angle that maximized the intensity of birefringence in secondary wall stage fibres. All other optical conditions were held constant between samples, micrographs were recorded at the same exposure time (optimized for *G. hirsutum *cv. TM-1 on 20 DPA) and image processing was omitted except for conversion to grayscale. For both DIC images, the Levels function in Adobe Photoshop was adjusted linearly and equally to optimize the tonal range for viewing.

### Isolation of total RNA from fibres

In order to separate fibres from the ovules, cooled glass beads were combined with the ovules and mechanically agitated under liquid nitrogen, using a modification of a published procedure [73]. Samples were examined microscopically for ovular or other debris before total RNA was extracted from the sheared fibres using a hot borate/lithium chloride procedure [74]. Purified RNA samples were quantified using a NanoDrop Spectrophotometer (Thermo Fisher Scientific Inc, MA, USA) and checked for integrity on a BioAnalyzer chip (Agilent, CA, USA).

### Amplification of RNA and microarray hybridizations

We extracted RNA from 30 samples representing two accessions, five time points and three biological replicates. These samples were treated with DNAse according to the manufacture's protocol (New England Biolabs, MA, USA) and linearly amplified using the TargetAmp™1-Round aRNA Amplification kit from Epicentre Biotechnologies (WI, USA). Following amplification, the integrity of the RNA was checked on a BioAnalyzer for contamination and degradation. For each of the 30 amplified RNAs, a total of 12 μg was shipped to Nimblegen Systems, Inc (WI, USA) for cDNA synthesis, labelling and hybridization to a custom cotton microarray. The microarray probes were designed from a global assembly of ~270,000 Sanger-based ESTs derived from *G. arboreum*, *G. raimondii *and *G. hirsutum *[75]. The custom cotton microarray contains 283,000 features that interrogate the relative expression intensity of 42,430 unigenes using an average of seven distinct probes per unigene. Probes averaged 60 bp in length, and, whenever possible, were designed to avoid single nucleotide polymorphisms between the A and the D genomes of the allopolyploid *G. hirsutum*. This was unproblematic for nearly all oligonucleotides, as the two diploids differ by only about 1% in their exonic sequences [76]. Where possible, probes were designed to independently interrogate paralogs. Additional information about the specifics of the chip and its design can be found at http://www.cottonevolution.info.

The cotton microarray was validated using quantitative PCR (qPCR) and mass-spectrometry (Sequenom, CA, USA) estimates of gene expression [19,20,77-80]. In addition, in an analysis of the 10 vesus 20 DPA comparison in TM-1, many genes expected to be up-regulated for secondary wall deposition (as implicated by co-expression with secondary wall cellulose synthase genes in *Arabidopsis*) displayed the expected up-regulation [26]. In that study, six genes (100% of those tested) were validated by qPCR to be up-regulated from 20 - 30 DPA during secondary wall deposition. Additional genes having diverse expression profiles throughout fibre development, as judged by the TM-1 microarray data, have been shown by qPCR to have the same expression pattern in independent analyses [45,46]. Finally, as shown below, the microarray data are consistent with well-established features of cotton fibre development.

### Statistical analysis

Raw expression values for each unigene represented on the chip were obtained by median polishing the seven redundant probes using Tukey's Biweight estimator [81,82]. In R [83], polished values were natural log transformed, median centred and scale normalized. In order to assess differential expression we applied a standard mixed linear model to each gene in Ststistcal Analysis Software (SAS) [84] taking the form:

$$y_{ijk}=\mu +\delta_{i}+\tau_{j}+s_{k}+\delta \tau_{ij}+e_{ijkm}$$

where the response variable *y*_ijk _denotes signal intensity for genotype *i*, time-point *j *and biological replication *k *and is described by *μ*, intercept parameter, *δ_i _*, the fixed effect of genotype *I*, *τ*_*j *_the fixed effect of time-point *j*, *s*_*k *_the random effect of replication *k*, *δτ*_*ij *_the interaction between genotype *i *and time-point *j *and *e_ijk _*the random error. We estimated the difference between chronological time points within accessions, simultaneous time points between accessions and chronological time points between accessions. In R, we used the method of Storey and Tibshirani to control the FDR [85]. The resultant 40,430 *q*-values per contrast were then used to identify genes that were differentially expressed for a given contrast, with the criteria of significance being a *q*-value ≤ 0.05. All array data have been deposited in compliance with MIAME (minimum information about a microarray experiment) standards on the NCBI GEO (gene expression omnibus) website and can be found under the data set record GSE23517. Additional File 1: Table S1 lists the differentially expressed genes between the two accessions at 2, 7, 10, 20 and 25 DPA, along with annotations of the closest homolog in *Arabidopsis*.

### Functional analysis of microarray data using GO annotations

The comparisons included in this analysis were: (a) within accessions, 2 versus 7 DPA, 7 versus 10 DPA and 20 versus 25 DPA (TM-1 only); and (b) between accessions, 2, 7, 10, 20 and 25 DPA. Statistically significant differentially expressed genes were binned into up- and down-regulated classes based on an estimated expression level under the SAS model. This produced 20 lists of up- and down-regulated genes both within an individual accession and between accessions across developmental time. We retrieved GO http://www.geneontology.org/ annotations as assigned to the unigenes in the microarray probe data set and used Fisher's exact test as implemented by Blast2GO to check for enriched GO categories when the tests sets were compared to the total query [86]. GO terms were only included if they were significantly enriched after correcting for the false discovery rate, at a *q*-value ≤ 0.05. The lists, associated GO categories and probe annotations are reported in Additional File 2: Table S2.

### Medoids clustering

For all genes diagnosed as differentially expressed between wild and domesticated *G. hirsutum*, estimates of their expression intensities according to the SAS model described above were recovered using *lsmeans*. These values were standardized on a global basis to remove magnitude, from which we calculated a simple Euclidean distance matrix for each pairwise gene comparison. In order to determine the optimal number of clusters in the data, we explored the fit of varying numbers of PAM clusters from 1 to 15 and calculated *k*, the silhouette width, for each *n *number of clusters versus *n+1 *[23]. We used these standardized distances and the gap statistic to PAM cluster wild and domesticated values separately. A gene expression pattern transition matrix was created by tabulating the expression state (PAM cluster) in the wild fibre versus the domesticated fibre data set. The resulting gene clusters are tabulated in Additional File 3: Table S3, which will be explained below.

GO annotations were assigned to genes in each PAM cluster and the annotations in each group were statistically analysed for up- and down-regulated GO classes. This analysis implemented the statistical test of GO class abundance described above, using a significance threshold with a *q*-value ≤ 0.05 [85]. This test was automated using a custom script. The results are tabulated in Additional File 4: Table S4.

## Abbreviations

DIC: differential interference contrast; DPA: days post anthesis; FDR: false discovery rate; GO: gene ontology; ISI1: impaired sucrose induction 1; PAM: partitioning around medoids; POL: polarizing; qPCR: quantitative polymerase chain reaction; QTL: quantitative trait locus; RFLP: restriction fragment length polymorphism; ROS: reactive oxygen species; SUS: sucrose synthase; UDP: uridine diphosphate glucose.

## Authors' contributions

RR carried out the comparative gene expression studies, participated in data analysis and generated an early version of the manuscript. CH conducted the microscopy studies, participated in the data analysis and contributed to the manuscript preparation. LF and RH participated in data analysis and the manuscript preparation. JU designed the microarray used in the experiments and participated in the manuscript preparation. JW conceived the study, participated in its design and coordination and led the development of the final draft the manuscript. All authors read and approved the final manuscript.

## Supplementary Material

Additional file 1 **Table S1**. Differentially expressed genes between wild and cultivated cotton at 2, 7, 10, 20 and 25 days post anthesis along with gene ontology annotations. Click here for file

Additional file 2 **Table S2**. Enriched gene ontology annotations for multiple comparisons of differentially expressed genes. Within accessions, comparisons include 2 versus 7 days post anthesis (DPA), 7 versus 10 DPA, and 20 versus 25 DPA (TM-1 only). Between accessions, comparisons were made at 2, 7, 10, 20 and 25 DPA. Click here for file

Additional file 3 **Table S3**. Cotton genes in each block of the transition matrix of Figure 4, along with *Arabidopsis *homologs and gene ontology annotations. Click here for file

Additional file 4 **Table S4**. Over- and under-abundant gene ontology (GO) annotations, corresponding cotton genes and *Arabidopsis *homologs from blocks of the transition matrix that showed statistically significant differences in GO annotations compared to expected values (Figure 4). Click here for file
