# Supplementary material for: Gene expression in developing fibres of Upland cotton (Gossypium hirsutum L.) was massively altered by domestication
Source: BMC Biol. 2010 Nov 15;8:139. doi: 10.1186/1741-7007-8-139 (PMC2992495; doi:10.1186/1741-7007-8-139)
Supplement: Additional file 2 — Table S2. Enriched gene ontology annotations for multiple comparisons of differentially expressed genes. Within accessions, comparisons include 2 versus 7 days post anthesis (DPA), 7 versus 10 DPA, and 20 versus 25 DPA (TM-1 only). Between accessions, comparisons were made at 2, 7, 10, 20 and 25 DPA. [file 1741-7007-8-139-S2.PDF]

GOSSIP
Test-Set: tm1.2.7.up.txt
Tests for all terms in Gene Ontology whether it is enriched in a test group when compared to a reference group using Fisher's excact test with Multiple Testing.
Pub: Biological Profiling of Gene Groups utilizing Gene Ontology A Statistical Framework
Poster: GOSSIP: Biological Profiling of Gene Groups utilizing Gene Ontology
by Nils Blthgen, Karsten Brand, Hanspeter Herzel, Dieter Beule

GO Term	Name	FDR	FWER	single test p-Value	# in test group	# in reference group	# non annot test	# non annot reference group	Over/Under
GO:0030529	ribonucleoprotein complex	9.74709E-9	1.3424E-8	0.0	166	1600	1136	39633	over
GO:0009059	macromolecule biosynthetic process	9.74709E-9	1.3424E-8	0.0	204	3018	1098	38215	over
GO:0022613	ribonucleoprotein complex biogenesis and assembly	9.74709E-9	1.3424E-8	0.0	73	767	1229	40466	over
GO:0006412	translation	9.74709E-9	1.42731E-8	1.22489E-12	190	2052	1112	39181	over
GO:0042254	ribosome biogenesis and assembly	9.74709E-9	1.54338E-8	7.94329E-12	64	541	1238	40692	over
GO:0005739	mitochondrion	9.74709E-9	1.66555E-8	9.8455E-12	432	9587	870	31646	over
GO:0005198	structural molecule activity	9.74709E-9	2.0661E-8	2.62021E-11	146	1719	1156	39514	over
GO:0043232	intracellular non-membrane-bound organelle	9.74709E-9	2.19309E-8	3.18873E-11	178	2658	1124	38575	over
GO:0043228	non-membrane-bound organelle	9.74709E-9	2.19309E-8	3.18873E-	178	2658	1124	38575	over

				11					
GO:0005840	ribosome	9.80114E-9	2.53516E-8	4.18789E-11	150	1447	1152	39786	over
GO:0003735	structural constituent of ribosome	9.80114E-9	2.73984E-8	5.80868E-11	139	1356	1163	39877	over
GO:0044249	cellular biosynthetic process	9.80114E-9	3.10187E-8	6.69833E-11	281	4399	1021	36834	over
GO:0044444	cytoplasmic part	9.80114E-9	3.22729E-8	7.15215E-11	878	23977	424	17256	over
GO:0005737	cytoplasm	9.80114E-9	3.4304E-8	8.09068E-11	905	24673	397	16560	over
GO:0009058	biosynthetic process	1.22839E-8	5.35098E-8	1.12343E-10	304	5749	998	35484	over
GO:0033279	ribosomal subunit	1.22839E-8	5.3726E-8	1.15599E-10	87	538	1215	40695	over
GO:0015934	large ribosomal subunit	1.22839E-8	5.41778E-8	1.1668E-10	61	227	1241	41006	over
GO:0003723	RNA binding	1.22839E-8	5.52777E-8	1.20361E-10	93	1281	1209	39952	over
GO:0006396	RNA processing	1.15326E-6	5.47797E-6	1.75965E-8	44	517	1258	40716	over
GO:0005622	intracellular	2.19976E-6	1.09987E-5	3.38068E-8	983	28271	319	12962	over
GO:0044424	intracellular part	2.46687E-6	1.2951E-5	3.90342E-8	972	27912	330	13321	over

GO:0043229	intracellular organelle	6.7834E-6	3.7308E-5	1.28128E-7	934	26756	368	14477	over
GO:0043226	organelle	7.04066E-6	4.0483E-5	1.30559E-7	934	26758	368	14475	over
GO:0046835	carbohydrate phosphorylation	1.3241E-5	7.94429E-5	2.22568E-7	7	9	1295	41224	over
GO:0019843	rRNA binding	1.0081E-4	6.29864E-4	1.8181E-6	21	186	1281	41047	over
GO:0043231	intracellular membrane-bound organelle	1.1106E-4	7.4297E-4	2.0064E-6	895	25769	407	15464	over
GO:0043227	membrane-bound organelle	1.1106E-4	7.49371E-4	2.05798E-6	895	25772	407	15461	over
GO:0044267	cellular protein metabolic process	1.84495E-4	0.00132682	3.76406E-6	308	7653	994	33580	over
GO:0044260	cellular macromolecule metabolic process	1.84495E-4	0.0013367	3.80449E-6	308	7654	994	33579	over
GO:0044445	cytosolic part	2.96007E-4	0.00227052	5.97523E-6	29	346	1273	40887	over
GO:0006399	tRNA metabolic process	2.96007E-4	0.00229143	6.19123E-6	20	186	1282	41047	over
GO:0005830	cytosolic ribosome (sensu Eukaryota)	3.10094E-4	0.00247768	7.03554E-6	23	239	1279	40994	over
GO:0019538	protein metabolic process	3.16483E-4	0.00260758	7.45341E-6	310	7776	992	33457	over
GO:0004532	exoribonuclease activity	3.94076E-4	0.00363857	1.05628E-5	5	6	1297	41227	over
GO:0000175	3'-5'-exoribonuclease activity	3.94076E-4	0.00363857	1.05628E-5	5	6	1297	41227	over
GO:0016796	exonuclease activity, active with either ribo- or deoxyribonucleic acids and producing 5'-phosphomonoesters	3.94076E-4	0.00363857	1.05628E-5	5	6	1297	41227	over
GO:0016896	exoribonuclease activity, producing 5'-phosphomonoesters	3.94076E-4	0.00363857	1.05628E-5	5	6	1297	41227	over

GO:0015935	small ribosomal subunit	7.36617E-4	0.00697344	1.84621E-5	26	311	1276	40922	over
GO:0004527	exonuclease activity	0.00168186	0.0162644	4.10895E-5	9	46	1293	41187	over
GO:0032991	macromolecular complex	0.00181095	0.0179466	4.55637E-5	211	5111	1091	36122	over
GO:0004335	galactokinase activity	0.00259676	0.0262658	6.81471E-5	6	18	1296	41215	over
GO:0005730	nucleolus	0.00318217	0.032861	8.94393E-5	20	228	1282	41005	over
GO:0004518	nuclease activity	0.00447765	0.0473819	1.11996E-4	21	251	1281	40982	over
GO:0005759	mitochondrial matrix	0.00447765	0.0491263	1.20219E-4	12	96	1290	41137	over
GO:0031980	mitochondrial lumen	0.00447765	0.0491263	1.20219E-4	12	96	1290	41137	over
GO:0004239	methionyl aminopeptidase activity	0.0050293	0.0561967	1.38552E-4	6	21	1296	41212	over
GO:0009536	plastid	0.0052254	0.0595522	1.57303E-4	418	11326	884	29907	over
GO:0006414	translational elongation	0.00621972	0.0747082	1.97801E-4	19	224	1283	41009	over
GO:0000313	organellar ribosome	0.00621972	0.07511	1.99083E-4	9	58	1293	41175	over
GO:0005843	cytosolic small ribosomal subunit (sensu Eukaryota)	0.00621972	0.0769853	2.08466E-4	19	225	1283	41008	over
GO:0031974	membrane-enclosed lumen	0.00621972	0.077675	2.10985E-4	40	673	1262	40560	over
GO:0043233	organelle lumen	0.00621972	0.077675	2.10985E-4	40	673	1262	40560	over
GO:0044452	nucleolar part	0.00723833	0.0930965	2.49874E-4	9	60	1293	41173	over

GO:0005732	small nucleolar ribonucleoprotein complex	0.00723833	0.0930965	2.49874E-4	9	60	1293	41173	over
GO:0004019	adenylosuccinate synthase activity	0.00862113	0.111786	2.73219E-4	3	2	1299	41231	over
GO:0000315	organellar large ribosomal subunit	0.0116967	0.153144	4.06543E-4	7	38	1295	41195	over
GO:0003746	translation elongation factor activity	0.0116967	0.153532	4.09022E-4	15	163	1287	41070	over
GO:0006418	tRNA aminoacylation for protein translation	0.0122242	0.167539	4.59307E-4	14	147	1288	41086	over
GO:0043038	amino acid activation	0.0122242	0.167539	4.59307E-4	14	147	1288	41086	over
GO:0043039	tRNA aminoacylation	0.0122242	0.167539	4.59307E-4	14	147	1288	41086	over
GO:0003676	nucleic acid binding	0.0123204	0.172748	4.83792E-4	230	5889	1072	35344	over
GO:0006996	organelle organization and biogenesis	0.0123204	0.173845	4.93392E-4	88	1917	1214	39316	over
GO:0009451	RNA modification	0.0150284	0.210778	5.34315E-4	7	40	1295	41193	over
GO:0048653	anther development	0.0155892	0.220762	5.88851E-4	8	54	1294	41179	over
GO:0005761	mitochondrial ribosome	0.0155941	0.223855	6.09177E-4	7	41	1295	41192	over
GO:0031981	nuclear lumen	0.0190035	0.269172	7.7134E-4	27	422	1275	40811	over
GO:0031365	N-terminal protein amino acid modification	0.0221866	0.310406	9.10304E-4	4	11	1298	41222	over
GO:0003697	single-stranded DNA binding	0.0235806	0.330282	9.13126E-4	3	4	1299	41229	over
GO:0015462	protein-transmembrane transporting ATPase activity	0.0239763	0.365924	9.3628E-4	2	0	1300	41233	over

GO:0043543	protein amino acid acylation	0.0239763	0.365924	9.3628E-4	2	0	1300	41233	over
GO:0018409	peptide or protein amino-terminal blocking	0.0239763	0.365924	9.3628E-4	2	0	1300	41233	over
GO:0018004	N-terminal protein formylation	0.0239763	0.365924	9.3628E-4	2	0	1300	41233	over
GO:0018256	protein amino acid formylation	0.0239763	0.365924	9.3628E-4	2	0	1300	41233	over
GO:0019988	charged-tRNA modification	0.0239763	0.365924	9.3628E-4	2	0	1300	41233	over
GO:0004641	phosphoribosylformylglycinamidine cyclo-ligase activity	0.0239763	0.365924	9.3628E-4	2	0	1300	41233	over
GO:0001718	conversion of met-tRNAf to fmet-tRNA	0.0239763	0.365924	9.3628E-4	2	0	1300	41233	over
GO:0009282	cytosolic large ribosomal subunit (sensu Bacteria)	0.0240435	0.374301	9.7006E-4	6	32	1296	41201	over
GO:0009281	cytosolic ribosome (sensu Bacteria)	0.0240435	0.374301	9.7006E-4	6	32	1296	41201	over
GO:0016776	phosphotransferase activity, phosphate group as acceptor	0.029958	0.446632	0.00132664	8	62	1294	41171	over
GO:0018175	protein amino acid nucleotidylation	0.0321215	0.478232	0.00142774	3	5	1299	41228	over
GO:0018117	protein amino acid adenylylation	0.0321215	0.478232	0.00142774	3	5	1299	41228	over
GO:0009396	folic acid and derivative biosynthetic process	0.0329563	0.491191	0.00146163	6	35	1296	41198	over
GO:0048443	stamen development	0.0338427	0.508741	0.00159434	8	64	1294	41169	over
GO:0048466	androecium development	0.0338427	0.508741	0.00159434	8	64	1294	41169	over
GO:0008408	3'-5' exonuclease activity	0.0349191	0.528038	0.00171706	5	24	1297	41209	over

GO:0005762	mitochondrial large ribosomal subunit	0.0349191	0.528038	0.00171706	5	24	1297	41209	over
GO:0005842	cytosolic large ribosomal subunit (sensu Eukaryota)	0.0373293	0.556043	0.00189663	4	14	1298	41219	over
GO:0006760	folic acid and derivative metabolic process	0.0435168	0.61616	0.00212314	6	38	1296	41195	over
GO:0009585	red, far-red light phototransduction	0.0444581	0.629026	0.00232732	7	53	1295	41180	over
GO:0051087	chaperone binding	0.0444581	0.641777	0.00234462	4	15	1298	41218	over
GO:0016875	ligase activity, forming carbon-oxygen bonds	0.0444581	0.645874	0.00238393	14	177	1288	41056	over
GO:0004812	aminoacyl-tRNA ligase activity	0.0444581	0.645874	0.00238393	14	177	1288	41056	over
GO:0016876	ligase activity, forming aminoacyl-tRNA and related compounds	0.0444581	0.645874	0.00238393	14	177	1288	41056	over
GO:0008033	tRNA processing	0.0444581	0.648293	0.00238724	6	39	1296	41194	over
GO:0031202	RNA splicing factor activity, transesterification mechanism	0.0452744	0.658863	0.00256072	7	54	1295	41179	over

GOSSIP
Test-Set: tm1.2.7.down.txt
Tests for all terms in Gene Ontology whether it is enriched in a test group when compared to a reference group using Fisher's excact test with Multiple Testing.
Pub: Biological Profiling of Gene Groups utilizing Gene Ontology A Statistical Framework
Poster: GOSSIP: Biological Profiling of Gene Groups utilizing Gene Ontology
by Nils Blthgen, Karsten Brand, Hanspeter Herzel, Dieter Beule

GO Term	Name	FDR	FWER	single test p-Value	# in test group	# in reference group	# non annot test	# non annot reference group	Over/Under
GO:0015288	porin activity	1.73977E-8	2.22814E-8	0.0	29	120	1140	41246	over

GO:0016020	membrane	1.73977E-8	2.22814E-8	0.0	378	9714	791	31652	over
GO:0022829	wide pore channel activity	1.73977E-8	2.22814E-8	0.0	29	120	1140	41246	over
GO:0004351	glutamate decarboxylase activity	1.73977E-8	3.04192E-8	3.06728E-11	9	3	1160	41363	over
GO:0015250	water channel activity	1.73977E-8	3.21094E-8	4.1104E-11	19	42	1150	41324	over
GO:0005372	water transporter activity	1.73977E-8	3.21094E-8	4.1104E-11	19	42	1150	41324	over
GO:0022803	passive transmembrane transporter activity	1.73977E-8	3.47954E-8	4.71983E-11	30	240	1139	41126	over
GO:0015267	channel activity	1.73977E-8	3.47954E-8	4.71983E-11	30	240	1139	41126	over
GO:0051234	establishment of localization	1.98932E-8	5.0185E-8	9.12183E-11	253	5972	916	35394	over
GO:0051179	localization	1.98932E-8	5.34892E-8	1.13355E-10	253	5996	916	35370	over
GO:0019867	outer membrane	1.98932E-8	5.47064E-8	1.18835E-10	31	218	1138	41148	over
GO:0006810	transport	2.48899E-8	7.46698E-8	1.97734E-10	250	5955	919	35411	over
GO:0051258	protein polymerization	8.80492E-8	2.8616E-7	9.02779E-10	26	200	1143	41166	over
GO:0007155	cell adhesion	2.5927E-7	9.72261E-7	3.06509E-9	15	63	1154	41303	over
GO:0022610	biological adhesion	2.5927E-7	9.72261E-7	3.06509E-9	15	63	1154	41303	over
GO:0047461	(+)-delta-cadinene synthase activity	2.66059E-7	1.06423E-6	3.3296E-9	8	8	1161	41358	over
GO:0006950	response to stress	2.58097E-6	1.09691E-5	3.35341E-8	123	2586	1046	38780	over

GO:0004022	alcohol dehydrogenase activity	9.25266E-6	4.16361E-5	1.15548E-7	15	86	1154	41280	over
GO:0007018	microtubule-based movement	1.01936E-5	4.84187E-5	1.5265E-7	26	263	1143	41103	over
GO:0006536	glutamate metabolic process	1.49839E-5	7.49168E-5	2.4509E-7	9	25	1160	41341	over
GO:0030705	cytoskeleton-dependent intracellular transport	4.4741E-5	2.34863E-4	6.30886E-7	26	285	1143	41081	over
GO:0022838	substrate specific channel activity	4.81315E-5	2.64688E-4	7.17588E-7	20	179	1149	41187	over
GO:0000813	ESCRT I complex	5.28623E-5	3.03912E-4	8.12593E-7	5	3	1164	41363	over
GO:0048029	monosaccharide binding	5.77796E-5	3.61057E-4	9.65171E-7	7	14	1162	41352	over
GO:0048032	galacturonate binding	5.77796E-5	3.61057E-4	9.65171E-7	7	14	1162	41352	over
GO:0005874	microtubule	7.79515E-5	5.06557E-4	1.44393E-6	26	299	1143	41067	over
GO:0022857	transmembrane transporter activity	1.17725E-4	7.94327E-4	2.40241E-6	85	1752	1084	39614	over
GO:0000323	lytic vacuole	1.53163E-4	0.00114071	3.16224E-6	10	47	1159	41319	over
GO:0009415	response to water	1.53163E-4	0.00117243	3.35816E-6	31	418	1138	40948	over
GO:0031988	membrane-bound vesicle	1.53163E-4	0.00118631	3.46462E-6	194	4967	975	36399	over
GO:0031982	vesicle	1.53163E-4	0.00118631	3.46462E-6	194	4967	975	36399	over
GO:0031410	cytoplasmic vesicle	2.07827E-4	0.00171311	4.988E-6	193	4966	976	36400	over
GO:0016023	cytoplasmic membrane-bound vesicle	2.07827E-4	0.00171311	4.988E-6	193	4966	976	36400	over

GO:0050896	response to stimulus	3.63214E-4	0.00308256	8.1472E-6	211	5567	958	35799	over
GO:0009628	response to abiotic stimulus	3.73984E-4	0.00333061	8.89578E-6	99	2209	1070	39157	over
GO:0042221	response to chemical stimulus	3.73984E-4	0.0033602	9.15029E-6	127	3020	1042	38346	over
GO:0007154	cell communication	4.90616E-4	0.00452792	1.2115E-5	94	2085	1075	39281	over
GO:0004194	pepsin A activity	5.6598E-4	0.00536239	1.53946E-5	15	133	1154	41233	over
GO:0007017	microtubule-based process	6.67714E-4	0.00648908	1.78677E-5	26	348	1143	41018	over
GO:0009414	response to water deprivation	7.30786E-4	0.00738921	1.94025E-5	28	393	1141	40973	over
GO:0016838	carbon-oxygen lyase activity, acting on phosphates	7.30786E-4	0.00746258	1.96038E-5	8	35	1161	41331	over
GO:0004553	hydrolase activity, hydrolyzing O-glycosyl compounds	9.13881E-4	0.00954987	2.38183E-5	43	750	1126	40616	over
GO:0046423	allene-oxide cyclase activity	9.33532E-4	0.010309	2.53074E-5	5	9	1164	41357	over
GO:0010286	heat acclimation	9.33532E-4	0.010309	2.53074E-5	5	9	1164	41357	over
GO:0003824	catalytic activity	9.33532E-4	0.0104473	2.6484E-5	577	17932	592	23434	over
GO:0004096	catalase activity	0.00105799	0.0120931	3.26819E-5	7	27	1162	41339	over
GO:0009341	beta-galactosidase complex	0.00138091	0.0160948	4.1683E-5	9	52	1160	41314	over
GO:0003924	GTPase activity	0.00150311	0.0178757	4.89509E-5	26	371	1143	40995	over
GO:0031224	intrinsic to membrane	0.00172263	0.0208812	6.01075E-5	87	1979	1082	39387	over

GO:0005576	extracellular region	0.00180163	0.0225578	6.26814E-5	26	377	1143	40989	over
GO:0016798	hydrolase activity, acting on glycosyl bonds	0.00180163	0.022709	6.34832E-5	44	810	1125	40556	over
GO:0007267	cell-cell signaling	0.00202318	0.0259585	6.96726E-5	7	31	1162	41335	over
GO:0030170	pyridoxal phosphate binding	0.00223296	0.0291535	7.86267E-5	17	193	1152	41173	over
GO:0008629	induction of apoptosis by intracellular signals	0.00224842	0.0337074	8.11255E-5	3	1	1166	41365	over
GO:0009394	2'-deoxyribonucleotide metabolic process	0.00224842	0.0337074	8.11255E-5	3	1	1166	41365	over
GO:0008631	induction of apoptosis by oxidative stress	0.00224842	0.0337074	8.11255E-5	3	1	1166	41365	over
GO:0009903	chloroplast avoidance movement	0.00224842	0.0337074	8.11255E-5	3	1	1166	41365	over
GO:0047429	nucleoside-triphosphate diphosphatase activity	0.00224842	0.0337074	8.11255E-5	3	1	1166	41365	over
GO:0046080	dUTP metabolic process	0.00224842	0.0337074	8.11255E-5	3	1	1166	41365	over
GO:0004170	dUTP diphosphatase activity	0.00224842	0.0337074	8.11255E-5	3	1	1166	41365	over
GO:0009904	chloroplast accumulation movement	0.00224842	0.0337074	8.11255E-5	3	1	1166	41365	over
GO:0010039	response to iron ion	0.00284761	0.0431784	1.04405E-4	4	6	1165	41360	over
GO:0000302	response to reactive oxygen species	0.00295747	0.0455124	1.17329E-4	18	220	1151	41146	over
GO:0010193	response to ozone	0.00324509	0.0508299	1.35369E-4	7	35	1162	41331	over
GO:0042542	response to hydrogen peroxide	0.00324509	0.0513669	1.38627E-4	17	203	1152	41163	over

GO:0016762	xyloglucan:xyloglucosyl transferase activity	0.00345271	0.0559954	1.53468E-4	10	78	1159	41288	over
GO:0004190	aspartic-type endopeptidase activity	0.00345271	0.0561929	1.54558E-4	17	205	1152	41161	over
GO:0030246	carbohydrate binding	0.00350499	0.0578451	1.60224E-4	18	226	1151	41140	over
GO:0009975	cyclase activity	0.00390392	0.065126	1.70833E-4	5	15	1164	41351	over
GO:0005764	lysosome	0.00408358	0.0689698	1.83323E-4	7	37	1162	41329	over
GO:0001676	long-chain fatty acid metabolic process	0.00465926	0.0793756	1.98649E-4	3	2	1166	41364	over
GO:0004696	glycogen synthase kinase 3 activity	0.00551926	0.0945729	2.35493E-4	4	8	1165	41358	over
GO:0005215	transporter activity	0.00615946	0.106893	2.88051E-4	103	2553	1066	38813	over
GO:0004565	beta-galactosidase activity	0.00615946	0.1077	2.88519E-4	8	54	1161	41312	over
GO:0005773	vacuole	0.00709252	0.124525	3.45158E-4	24	376	1145	40990	over
GO:0016021	integral to membrane	0.00727943	0.129174	3.55489E-4	82	1947	1087	39419	over
GO:0006665	sphingolipid metabolic process	0.00736639	0.132211	3.64812E-4	7	42	1162	41324	over
GO:0000169	activation of MAPK activity during osmolarity sensing	0.00768527	0.151363	3.89152E-4	3	3	1166	41363	over
GO:0043406	positive regulation of MAPK activity	0.00768527	0.151363	3.89152E-4	3	3	1166	41363	over
GO:0000161	MAPKKK cascade during osmolarity sensing	0.00768527	0.151363	3.89152E-4	3	3	1166	41363	over
GO:0043405	regulation of MAPK activity	0.00768527	0.151363	3.89152E-4	3	3	1166	41363	over

GO:0000187	activation of MAPK activity	0.00768527	0.151363	3.89152E-4	3	3	1166	41363	over
GO:0007231	osmosensory signaling pathway	0.00768527	0.151363	3.89152E-4	3	3	1166	41363	over
GO:0009898	internal side of plasma membrane	0.00768527	0.151363	3.89152E-4	3	3	1166	41363	over
GO:0007568	aging	0.00768527	0.15148	3.89526E-4	14	162	1155	41204	over
GO:0015630	microtubule cytoskeleton	0.00768527	0.152308	3.95066E-4	27	451	1142	40915	over
GO:0015925	galactosidase activity	0.00835111	0.166101	4.45048E-4	8	58	1161	41308	over
GO:0045735	nutrient reservoir activity	0.0087063	0.174318	4.55777E-4	11	108	1158	41258	over
GO:0006643	membrane lipid metabolic process	0.00924841	0.185992	5.21291E-4	18	251	1151	41115	over
GO:0030414	protease inhibitor activity	0.00931221	0.190923	5.29063E-4	7	45	1162	41321	over
GO:0004866	endopeptidase inhibitor activity	0.00931221	0.190923	5.29063E-4	7	45	1162	41321	over
GO:0006917	induction of apoptosis	0.0103067	0.217135	6.07969E-4	4	11	1165	41355	over
GO:0043065	positive regulation of apoptosis	0.0103067	0.217135	6.07969E-4	4	11	1165	41355	over
GO:0043068	positive regulation of programmed cell death	0.0103067	0.217135	6.07969E-4	4	11	1165	41355	over
GO:0012502	induction of programmed cell death	0.0103067	0.217135	6.07969E-4	4	11	1165	41355	over
GO:0006944	membrane fusion	0.0107256	0.228092	6.38839E-4	6	33	1163	41333	over
GO:0022892	substrate-specific transporter activity	0.0107256	0.229033	6.42241E-4	72	1695	1097	39671	over

GO:0016787	hydrolase activity	0.0108007	0.232511	6.62255E-4	207	5890	962	35476	over
GO:0048046	apoplast	0.012224	0.26249	7.42042E-4	12	134	1157	41232	over
GO:0009914	hormone transport	0.012224	0.26558	7.48837E-4	10	97	1159	41269	over
GO:0009926	auxin polar transport	0.012224	0.26558	7.48837E-4	10	97	1159	41269	over
GO:0048226	Casparian strip	0.0133097	0.29489	7.54701E-4	2	0	1167	41366	over
GO:0044426	cell wall part	0.0133097	0.29489	7.54701E-4	2	0	1167	41366	over
GO:0015708	silicate transport	0.0133097	0.29489	7.54701E-4	2	0	1167	41366	over
GO:0015115	silicate transmembrane transporter activity	0.0133097	0.29489	7.54701E-4	2	0	1167	41366	over
GO:0004091	carboxylesterase activity	0.0152269	0.334555	9.19912E-4	22	357	1147	41009	over
GO:0009631	cold acclimation	0.0152269	0.33609	9.28573E-4	7	50	1162	41316	over
GO:0009753	response to jasmonic acid stimulus	0.0152269	0.337116	9.37308E-4	18	265	1151	41101	over
GO:0042631	cellular response to water deprivation	0.0160979	0.355126	0.00101459	4	13	1165	41353	over
GO:0010101	post-embryonic root morphogenesis	0.0170846	0.378698	0.0010528	8	67	1161	41299	over
GO:0010102	lateral root morphogenesis	0.0170846	0.378698	0.0010528	8	67	1161	41299	over
GO:0009605	response to external stimulus	0.0170846	0.380231	0.00106555	36	716	1133	40650	over
GO:0044425	membrane part	0.0184523	0.406267	0.00124947	116	3072	1053	38294	over

GO:0003863	3-methyl-2-oxobutanoate dehydrogenase (2-methylpropanoyl-transferring) activity	0.0226882	0.485061	0.00153622	3	6	1166	41360	over
GO:0009211	pyrimidine deoxyribonucleoside triphosphate metabolic process	0.0226882	0.485061	0.00153622	3	6	1166	41360	over
GO:0046125	pyrimidine deoxyribonucleoside metabolic process	0.0226882	0.485061	0.00153622	3	6	1166	41360	over
GO:0009120	deoxyribonucleoside metabolic process	0.0226882	0.485061	0.00153622	3	6	1166	41360	over
GO:0004673	protein histidine kinase activity	0.0240262	0.510744	0.00168597	7	56	1162	41310	over
GO:0016775	phosphotransferase activity, nitrogenous group as acceptor	0.0240262	0.510744	0.00168597	7	56	1162	41310	over
GO:0000155	two-component sensor activity	0.0243185	0.521385	0.00174547	6	41	1163	41325	over
GO:0016831	carboxy-lyase activity	0.0243185	0.52343	0.00176902	19	305	1150	41061	over
GO:0043167	ion binding	0.0243185	0.52375	0.00177472	168	4751	1001	36615	over
GO:0051649	establishment of cellular localization	0.0247723	0.533199	0.00186732	120	3235	1049	38131	over
GO:0009719	response to endogenous stimulus	0.0248121	0.536655	0.00189406	87	2220	1082	39146	over
GO:0016843	amine-lyase activity	0.0255338	0.552657	0.0019343	4	16	1165	41350	over
GO:0016844	strictosidine synthase activity	0.0255338	0.552657	0.0019343	4	16	1165	41350	over
GO:0051641	cellular localization	0.0261092	0.563554	0.00200333	120	3242	1049	38124	over
GO:0010015	root morphogenesis	0.0263436	0.571704	0.00211518	13	174	1156	41192	over
GO:0044430	cytoskeletal part	0.0263436	0.572462	0.00212457	29	561	1140	40805	over

GO:0004322	ferroxidase activity	0.0272379	0.595785	0.00214985	3	7	1166	41359	over
GO:0009200	deoxyribonucleoside triphosphate metabolic process	0.0272379	0.595785	0.00214985	3	7	1166	41359	over
GO:0009875	pollen-pistil interaction	0.0272379	0.595785	0.00214985	3	7	1166	41359	over
GO:0016724	oxidoreductase activity, oxidizing metal ions, oxygen as acceptor	0.0272379	0.595785	0.00214985	3	7	1166	41359	over
GO:0042335	cuticle development	0.0273985	0.601312	0.00217014	6	43	1163	41323	over
GO:0009886	post-embryonic morphogenesis	0.0273985	0.603411	0.0021926	8	76	1161	41290	over
GO:0016328	lateral plasma membrane	0.0298776	0.645992	0.00222269	2	1	1167	41365	over
GO:0010082	regulation of root meristem size	0.0298776	0.645992	0.00222269	2	1	1167	41365	over
GO:0010329	auxin efflux transmembrane transporter activity	0.0298776	0.645992	0.00222269	2	1	1167	41365	over
GO:0003785	actin monomer binding	0.0298776	0.645992	0.00222269	2	1	1167	41365	over
GO:0009395	phospholipid catabolic process	0.0306949	0.664065	0.00233787	4	17	1165	41349	over
GO:0046466	membrane lipid catabolic process	0.0306949	0.664065	0.00233787	4	17	1165	41349	over
GO:0005529	sugar binding	0.0306949	0.6646	0.00234805	12	155	1157	41211	over
GO:0019842	vitamin binding	0.0306949	0.667172	0.00240441	19	314	1150	41052	over
GO:0000038	very-long-chain fatty acid metabolic process	0.0306949	0.671396	0.00240958	6	44	1163	41322	over
GO:0004427	inorganic diphosphatase activity	0.0306949	0.671396	0.00240958	6	44	1163	41322	over

GO:0019740	nitrogen utilization	0.0313179	0.683731	0.00254392	5	30	1164	41336	over
GO:0006808	regulation of nitrogen utilization	0.0313179	0.683731	0.00254392	5	30	1164	41336	over
GO:0009734	auxin mediated signaling pathway	0.0327067	0.701924	0.00278474	13	180	1156	41186	over
GO:0004186	carboxypeptidase C activity	0.0332213	0.709967	0.00279579	4	18	1165	41348	over
GO:0000165	MAPKKK cascade	0.0355519	0.736448	0.00289587	3	8	1166	41358	over
GO:0030599	pectinesterase activity	0.0361731	0.744838	0.00306121	10	119	1159	41247	over
GO:0005886	plasma membrane	0.0365345	0.750588	0.00316658	38	822	1131	40544	over
GO:0009269	response to desiccation	0.0368196	0.755538	0.00324765	6	47	1163	41319	over
GO:0051171	regulation of nitrogen metabolic process	0.0369561	0.759049	0.00326502	5	32	1164	41334	over
GO:0004857	enzyme inhibitor activity	0.0384723	0.776601	0.00359917	9	102	1160	41264	over
GO:0007010	cytoskeleton organization and biogenesis	0.0384723	0.777053	0.00360615	31	637	1138	40729	over
GO:0031225	anchored to membrane	0.0392506	0.786639	0.00367589	5	33	1164	41333	over
GO:0015293	symporter activity	0.0392506	0.787928	0.00371123	15	232	1154	41134	over
GO:0042178	xenobiotic catabolic process	0.0411306	0.808972	0.00378266	3	9	1166	41357	over
GO:0003958	NADPH-hemoprotein reductase activity	0.0411306	0.808972	0.00378266	3	9	1166	41357	over
GO:0009266	response to temperature stimulus	0.0411306	0.812173	0.00386612	36	777	1133	40589	over

GO:0015833	peptide transport	0.0411306	0.812985	0.00386725	8	84	1161	41282	over
GO:0006857	oligopeptide transport	0.0411306	0.812985	0.00386725	8	84	1161	41282	over
GO:0004024	alcohol dehydrogenase activity, zinc-dependent	0.0422444	0.823167	0.00391577	6	49	1163	41317	over
GO:0007165	signal transduction	0.0422823	0.825299	0.00393603	78	2008	1091	39358	over
GO:0005941	unlocalized protein complex	0.0445263	0.842519	0.00429478	9	105	1160	41261	over

GOSSIP
Test-Set: tm1.10.20.up.txt
Tests for all terms in Gene Ontology whether it is enriched in a test group when compared to a reference group using Fisher's excact test with Multiple Testing.
Pub: Biological Profiling of Gene Groups utilizing Gene Ontology A Statistical Framework
Poster: GOSSIP: Biological Profiling of Gene Groups utilizing Gene Ontology
by Nils Blthgen, Karsten Brand, Hanspeter Herzel, Dieter Beule

GO Term	Name	FDR	FWER	single test p-Value	# in test group	# in reference group	# non annot test	# non annot reference group	Over/Under
GO:0016747	transferase activity, transferring groups other than amino-acyl groups	2.28542E-8	4.22375E-8	0.0	89	507	2968	38971	over
GO:0005576	extracellular region	2.28542E-8	4.22375E-8	0.0	69	334	2988	39144	over
GO:0031982	vesicle	2.28542E-8	4.22375E-8	0.0	514	4647	2543	34831	over
GO:0031988	membrane-bound vesicle	2.28542E-8	4.22375E-8	0.0	514	4647	2543	34831	over
GO:0016023	cytoplasmic membrane-bound vesicle	2.28542E-8	4.22375E-8	0.0	514	4645	2543	34833	over

GO:0031410	cytoplasmic vesicle	2.28542E-8	4.22375E-8	0.0	514	4645	2543	34833	over
GO:0016746	transferase activity, transferring acyl groups	2.28542E-8	4.40985E-8	1.1271E-11	96	563	2961	38915	over
GO:0008415	acyltransferase activity	2.28542E-8	4.57083E-8	2.2311E-11	87	499	2970	38979	over
GO:0016798	hydrolase activity, acting on glycosyl bonds	8.33262E-8	1.87484E-7	4.61607E-10	112	742	2945	38736	over
GO:0003824	catalytic activity	1.19125E-7	2.97813E-7	6.86187E-10	1491	17018	1566	22460	over
GO:0004553	hydrolase activity, hydrolyzing O-glycosyl compounds	1.31778E-7	3.62389E-7	1.01838E-9	105	688	2952	38790	over
GO:0004650	polygalacturonase activity	1.08739E-6	3.26216E-6	9.57758E-9	23	59	3034	39419	over
GO:0016210	naringenin-chalcone synthase activity	5.29405E-6	1.72055E-5	4.9719E-8	13	17	3044	39461	over
GO:0006629	lipid metabolic process	2.11226E-5	7.39264E-5	1.94276E-7	155	1274	2902	38204	over
GO:0016020	membrane	6.3518E-5	2.38164E-4	6.62007E-7	837	9255	2220	30223	over
GO:0019748	secondary metabolic process	7.31669E-5	2.92625E-4	8.3199E-7	100	749	2957	38729	over
GO:0008610	lipid biosynthetic process	4.47482E-4	0.00189999	5.14531E-6	100	785	2957	38693	over
GO:0047461	(+)-delta-cadinene synthase activity	4.52338E-4	0.00203345	5.37257E-6	8	8	3049	39470	over
GO:0009698	phenylpropanoid metabolic process	5.20629E-4	0.00246993	6.30427E-6	54	346	3003	39132	over
GO:0016211	ammonia ligase activity	7.67776E-4	0.00402272	1.04617E-5	15	43	3042	39435	over
GO:0016880	acid-ammonia (or amide) ligase activity	7.67776E-4	0.00402272	1.04617E-5	15	43	3042	39435	over

GO:0045552	dihydrokaempferol 4-reductase activity	8.37767E-4	0.00469694	1.07844E-5	5	1	3052	39477	over
GO:0012505	endomembrane system	8.37767E-4	0.00480558	1.11426E-5	227	2171	2830	37307	over
GO:0003838	sterol 24-C-methyltransferase activity	8.57134E-4	0.00512961	1.14597E-5	7	6	3050	39472	over
GO:0006725	aromatic compound metabolic process	9.96306E-4	0.00659692	1.61107E-5	85	658	2972	38820	over
GO:0009812	flavonoid metabolic process	9.96306E-4	0.00684364	1.71141E-5	31	160	3026	39318	over
GO:0006575	amino acid derivative metabolic process	9.96306E-4	0.00694346	1.78382E-5	75	561	2982	38917	over
GO:0007167	enzyme linked receptor protein signaling pathway	9.96306E-4	0.00719721	1.86224E-5	40	236	3017	39242	over
GO:0007169	transmembrane receptor protein tyrosine kinase signaling pathway	9.96306E-4	0.00719721	1.86224E-5	40	236	3017	39242	over
GO:0009813	flavonoid biosynthetic process	0.00106136	0.00792861	2.11222E-5	29	146	3028	39332	over
GO:0004489	methylenetetrahydrofolate reductase (NADPH) activity	0.0010976	0.00847037	2.14905E-5	7	7	3050	39471	over
GO:0001932	regulation of protein amino acid phosphorylation	0.00110063	0.0120339	2.66321E-5	4	0	3053	39478	over
GO:0018108	peptidyl-tyrosine phosphorylation	0.00110063	0.0120339	2.66321E-5	4	0	3053	39478	over
GO:0048640	negative regulation of developmental growth	0.00110063	0.0120339	2.66321E-5	4	0	3053	39478	over
GO:0050730	regulation of peptidyl-tyrosine phosphorylation	0.00110063	0.0120339	2.66321E-5	4	0	3053	39478	over
GO:0001933	negative regulation of protein amino acid phosphorylation	0.00110063	0.0120339	2.66321E-5	4	0	3053	39478	over
GO:0045936	negative regulation of phosphate metabolic process	0.00110063	0.0120339	2.66321E-5	4	0	3053	39478	over

GO:0048638	regulation of developmental growth	0.00110063	0.0120339	2.66321E-5	4	0	3053	39478	over
GO:0045763	negative regulation of amino acid metabolic process	0.00110063	0.0120339	2.66321E-5	4	0	3053	39478	over
GO:0033239	negative regulation of amine metabolic process	0.00110063	0.0120339	2.66321E-5	4	0	3053	39478	over
GO:0050732	negative regulation of peptidyl-tyrosine phosphorylation	0.00110063	0.0120339	2.66321E-5	4	0	3053	39478	over
GO:0042326	negative regulation of phosphorylation	0.00110063	0.0120339	2.66321E-5	4	0	3053	39478	over
GO:0018212	peptidyl-tyrosine modification	0.00110063	0.0120339	2.66321E-5	4	0	3053	39478	over
GO:0030497	fatty acid elongation	0.00110063	0.0120339	2.66321E-5	4	0	3053	39478	over
GO:0006631	fatty acid metabolic process	0.00114161	0.0127611	2.8565E-5	69	511	2988	38967	over
GO:0004725	protein tyrosine phosphatase activity	0.00135515	0.0154635	3.54922E-5	5	2	3052	39476	over
GO:0006882	cellular zinc ion homeostasis	0.00139233	0.0169115	3.77852E-5	7	8	3050	39470	over
GO:0055069	zinc ion homeostasis	0.00139233	0.0169115	3.77852E-5	7	8	3050	39470	over
GO:0051592	response to calcium ion	0.00139233	0.0169115	3.77852E-5	7	8	3050	39470	over
GO:0008886	glyceraldehyde-3-phosphate dehydrogenase (NADP+) activity	0.00147634	0.0184937	4.05005E-5	8	12	3049	39466	over
GO:0006519	amino acid and derivative metabolic process	0.00147634	0.0188763	4.36452E-5	164	1519	2893	37959	over
GO:0044255	cellular lipid metabolic process	0.00147634	0.0190094	4.4331E-5	127	1118	2930	38360	over
GO:0008810	cellulase activity	0.00167322	0.0219263	5.06109E-5	11	27	3046	39451	over

GO:0008169	C-methyltransferase activity	0.00203808	0.0271391	6.2995E-5	7	9	3050	39469	over
GO:0006082	organic acid metabolic process	0.00239875	0.0332777	8.55644E-5	200	1944	2857	37534	over
GO:0008943	glyceraldehyde-3-phosphate dehydrogenase activity	0.00239875	0.0335621	8.57284E-5	17	67	3040	39411	over
GO:0007166	cell surface receptor linked signal transduction	0.00239875	0.0336048	8.6018E-5	49	338	3008	39140	over
GO:0015198	oligopeptide transporter activity	0.00269327	0.0383	8.90067E-5	5	3	3052	39475	over
GO:0019752	carboxylic acid metabolic process	0.00326806	0.0470609	1.14331E-4	199	1944	2858	37534	over
GO:0019438	aromatic compound biosynthetic process	0.00387641	0.0564885	1.33147E-4	51	364	3006	39114	over
GO:0042335	cuticle development	0.00398963	0.0590286	1.39688E-4	12	37	3045	39441	over
GO:0042325	regulation of phosphorylation	0.0045765	0.0684791	1.62221E-4	10	26	3047	39452	over
GO:0000178	exosome (RNase complex)	0.00511803	0.0774468	1.83002E-4	23	118	3034	39360	over
GO:0019187	beta-1,4-mannosyltransferase activity	0.00656698	0.102693	2.48586E-4	6	8	3051	39470	over
GO:0000030	mannosyltransferase activity	0.00656698	0.102693	2.48586E-4	6	8	3051	39470	over
GO:0051753	mannan synthase activity	0.00656698	0.102693	2.48586E-4	6	8	3051	39470	over
GO:0004930	G-protein coupled receptor activity	0.00683404	0.108475	2.64295E-4	10	28	3047	39450	over
GO:0008757	S-adenosylmethionine-dependent methyltransferase activity	0.00683404	0.109686	2.68844E-4	27	155	3030	39323	over
GO:0032787	monocarboxylic acid metabolic process	0.00692369	0.112579	2.83115E-4	88	754	2969	38724	over

GO:0048046	apoplast	0.00705324	0.11612	3.08794E-4	23	123	3034	39355	over
GO:0015368	calcium:cation antiporter activity	0.00736455	0.125765	3.30413E-4	7	13	3050	39465	over
GO:0015369	calcium:hydrogen antiporter activity	0.00736455	0.125765	3.30413E-4	7	13	3050	39465	over
GO:0051139	metal ion:hydrogen antiporter activity	0.00736455	0.125765	3.30413E-4	7	13	3050	39465	over
GO:0006542	glutamine biosynthetic process	0.00766893	0.132274	3.40144E-4	8	18	3049	39460	over
GO:0017077	oxidative phosphorylation uncoupler activity	0.00884992	0.156644	3.54971E-4	4	2	3053	39476	over
GO:0008934	inositol-1(or 4)-monophosphatase activity	0.00884992	0.156644	3.54971E-4	4	2	3053	39476	over
GO:0031403	lithium ion binding	0.00884992	0.156644	3.54971E-4	4	2	3053	39476	over
GO:0016711	flavonoid 3'-monooxygenase activity	0.00969523	0.17427	3.70895E-4	3	0	3054	39478	over
GO:0010241	ent-kaurene oxidase activity	0.00969523	0.17427	3.70895E-4	3	0	3054	39478	over
GO:0015995	chlorophyll biosynthetic process	0.00976156	0.177361	3.86586E-4	19	93	3038	39385	over
GO:0030675	Rac GTPase activator activity	0.0100158	0.18358	3.89119E-4	6	9	3051	39469	over
GO:0004185	serine carboxypeptidase activity	0.0100931	0.186911	3.99285E-4	14	56	3043	39422	over
GO:0051174	regulation of phosphorus metabolic process	0.0101739	0.192376	4.15241E-4	10	30	3047	39448	over
GO:0019220	regulation of phosphate metabolic process	0.0101739	0.192376	4.15241E-4	10	30	3047	39448	over
GO:0019751	polyol metabolic process	0.0104775	0.199608	4.52866E-4	15	64	3042	39414	over

GO:0006633	fatty acid biosynthetic process	0.0111133	0.212542	4.81477E-4	50	377	3007	39101	over
GO:0016853	isomerase activity	0.0112505	0.217068	5.0429E-4	61	488	2996	38990	over
GO:0016840	carbon-nitrogen lyase activity	0.0117653	0.22806	5.43933E-4	13	51	3044	39427	over
GO:0006261	DNA-dependent DNA replication	0.0127935	0.247738	6.0278E-4	18	89	3039	39389	over
GO:0008825	cyclopropane-fatty-acyl-phospholipid synthase activity	0.0131022	0.257766	6.11145E-4	5	6	3052	39472	over
GO:0051302	regulation of cell division	0.0131022	0.257766	6.11145E-4	5	6	3052	39472	over
GO:0015925	galactosidase activity	0.0147572	0.287827	7.40219E-4	13	53	3044	39425	over
GO:0004356	glutamate-ammonia ligase activity	0.0149402	0.29473	7.69718E-4	8	21	3049	39457	over
GO:0016838	carbon-oxygen lyase activity, acting on phosphates	0.0149402	0.296098	7.7065E-4	10	33	3047	39445	over
GO:0005677	chromatin silencing complex	0.0156809	0.314038	7.80943E-4	4	3	3053	39475	over
GO:0009699	phenylpropanoid biosynthetic process	0.0156809	0.315528	7.96016E-4	39	279	3018	39199	over
GO:0006270	DNA replication initiation	0.0156809	0.31774	8.01394E-4	9	27	3048	39451	over
GO:0016740	transferase activity	0.0156809	0.319185	8.15856E-4	527	5948	2530	33530	over
GO:0006071	glycerol metabolic process	0.0156809	0.321675	8.26444E-4	14	61	3043	39417	over
GO:0016053	organic acid biosynthetic process	0.0193807	0.387008	0.00103548	51	402	3006	39076	over
GO:0046394	carboxylic acid biosynthetic process	0.0193807	0.387008	0.00103548	51	402	3006	39076	over

GO:0016620	oxidoreductase activity, acting on the aldehyde or oxo group of donors, NAD or NADP as acceptor	0.0196005	0.393382	0.00109716	26	163	3031	39315	over
GO:0000904	cellular morphogenesis during differentiation	0.0205804	0.411391	0.00117414	17	87	3040	39391	over
GO:0009341	beta-galactosidase complex	0.0206023	0.414747	0.00118156	12	49	3045	39429	over
GO:0042398	amino acid derivative biosynthetic process	0.0219915	0.438608	0.00126931	55	447	3002	39031	over
GO:0005941	unlocalized protein complex	0.0220653	0.442778	0.00128938	18	96	3039	39382	over
GO:0004565	beta-galactosidase activity	0.0225908	0.453581	0.00137068	12	50	3045	39428	over
GO:0009394	2'-deoxyribonucleotide metabolic process	0.0242516	0.499051	0.00140369	3	1	3054	39477	over
GO:0046080	dUTP metabolic process	0.0242516	0.499051	0.00140369	3	1	3054	39477	over
GO:0008631	induction of apoptosis by oxidative stress	0.0242516	0.499051	0.00140369	3	1	3054	39477	over
GO:0004170	dUTP diphosphatase activity	0.0242516	0.499051	0.00140369	3	1	3054	39477	over
GO:0000340	RNA 7-methylguanosine cap binding	0.0242516	0.499051	0.00140369	3	1	3054	39477	over
GO:0008629	induction of apoptosis by intracellular signals	0.0242516	0.499051	0.00140369	3	1	3054	39477	over
GO:0047429	nucleoside-triphosphate diphosphatase activity	0.0242516	0.499051	0.00140369	3	1	3054	39477	over
GO:0016903	oxidoreductase activity, acting on the aldehyde or oxo group of donors	0.0258894	0.524987	0.00147829	31	213	3026	39265	over
GO:0016126	sterol biosynthetic process	0.0284276	0.567568	0.00162371	11	44	3046	39434	over
GO:0016125	sterol metabolic process	0.0284276	0.567568	0.00162371	11	44	3046	39434	over

GO:0006807	nitrogen compound metabolic process	0.0284276	0.567741	0.00162682	136	1337	2921	38141	over
GO:0005100	Rho GTPase activator activity	0.0288969	0.576754	0.00164293	6	13	3051	39465	over
GO:0001584	rhodopsin-like receptor activity	0.0315997	0.612539	0.00192431	8	25	3049	39453	over
GO:0009835	ripening	0.0325886	0.626923	0.00209323	12	53	3045	39425	over
GO:0004040	amidase activity	0.0328272	0.632718	0.00215362	9	32	3048	39446	over
GO:0009631	cold acclimation	0.0328272	0.635639	0.00219381	11	46	3046	39432	over
GO:0004764	shikimate 5-dehydrogenase activity	0.0346847	0.658845	0.00220653	5	9	3052	39469	over
GO:0016829	lyase activity	0.0414501	0.726265	0.00267195	113	1097	2944	38381	over
GO:0019740	nitrogen utilization	0.0424103	0.740901	0.00286896	8	27	3049	39451	over
GO:0006808	regulation of nitrogen utilization	0.0424103	0.740901	0.00286896	8	27	3049	39451	over
GO:0030247	polysaccharide binding	0.0424103	0.751013	0.00290162	6	15	3051	39463	over
GO:0001871	pattern binding	0.0424103	0.751013	0.00290162	6	15	3051	39463	over
GO:0006779	porphyrin biosynthetic process	0.0424103	0.755637	0.002943	20	122	3037	39356	over
GO:0015994	chlorophyll metabolic process	0.0424103	0.755637	0.002943	20	122	3037	39356	over
GO:0033014	tetrapyrrole biosynthetic process	0.0424103	0.755637	0.002943	20	122	3037	39356	over
GO:0043572	plastid fission	0.0424103	0.762203	0.00303551	7	21	3050	39457	over

GO:0009399	nitrogen fixation	0.0424103	0.762203	0.00303551	7	21	3050	39457	over
GO:0010020	chloroplast fission	0.0424103	0.762203	0.00303551	7	21	3050	39457	over
GO:0005884	actin filament	0.0424103	0.764309	0.00303768	10	41	3047	39437	over
GO:0015491	cation:cation antiporter activity	0.0424103	0.766112	0.00304463	9	34	3048	39444	over
GO:0006694	steroid biosynthetic process	0.0447183	0.7863	0.00318542	13	64	3044	39414	over

GOSSIP
Test-Set: tm1.10.20.down.txt
Tests for all terms in Gene Ontology whether it is enriched in a test group when compared to a reference group using Fisher's excact test with Multiple Testing.
Pub: Biological Profiling of Gene Groups utilizing Gene Ontology A Statistical Framework
Poster: GOSSIP: Biological Profiling of Gene Groups utilizing Gene Ontology
by Nils Blthgen, Karsten Brand, Hanspeter Herzel, Dieter Beule

GO Term	Name	FDR	FWER	single test p-Value	# in test group	# in reference group	# non annot test	# non annot reference group	Over/Under
GO:0016798	hydrolase activity, acting on glycosyl bonds	1.98928E-8	2.08808E-8	0.0	102	752	2279	39402	over
GO:0019222	regulation of metabolic process	1.98928E-8	2.16223E-8	2.49465E-12	247	2668	2134	37486	over
GO:0005976	polysaccharide metabolic process	1.98928E-8	2.71849E-8	2.65493E-11	30	86	2351	40068	over
GO:0000272	polysaccharide catabolic process	1.98928E-8	2.71849E-8	2.65493E-11	30	86	2351	40068	over

GO:0004553	hydrolase activity, hydrolyzing O-glycosyl compounds	1.98928E-8	3.71434E-8	5.14524E-11	101	692	2280	39462	over
GO:0009834	cellulose and pectin-containing secondary cell wall biogenesis	1.98928E-8	4.15992E-8	6.91051E-11	21	21	2360	40133	over
GO:0044247	cellular polysaccharide catabolic process	1.98928E-8	4.97282E-8	8.7714E-11	28	68	2353	40086	over
GO:0031323	regulation of cellular metabolic process	1.98928E-8	5.21244E-8	9.58512E-11	244	2620	2137	37534	over
GO:0050896	response to stimulus	1.98928E-8	5.45608E-8	1.07859E-10	433	5345	1948	34809	over
GO:0050794	regulation of cellular process	1.98928E-8	5.63504E-8	1.21544E-10	279	3043	2102	37111	over
GO:0045449	regulation of transcription	1.98928E-8	6.3579E-8	1.43059E-10	224	2402	2157	37752	over
GO:0016758	transferase activity, transferring hexosyl groups	1.98928E-8	6.45741E-8	1.52281E-10	95	757	2286	39397	over
GO:0050789	regulation of biological process	1.98928E-8	6.46517E-8	1.54227E-10	304	3521	2077	36633	over
GO:0065007	biological regulation	2.18136E-8	7.63478E-8	2.06243E-10	358	4289	2023	35865	over
GO:0019219	regulation of nucleobase, nucleoside, nucleotide and nucleic acid metabolic process	2.56347E-8	9.61299E-8	2.56101E-10	226	2438	2155	37716	over
GO:0016413	O-acetyltransferase activity	3.94584E-8	1.57834E-7	4.90825E-10	12	10	2369	40144	over
GO:0043169	cation binding	7.03462E-8	2.98971E-7	8.85469E-10	299	3506	2082	36648	over
GO:0043167	ion binding	9.0875E-8	4.08938E-7	1.28128E-9	370	4549	2011	35605	over
GO:0006350	transcription	1.00044E-7	4.75209E-7	1.49253E-9	234	2607	2147	37547	over
GO:0046914	transition metal ion binding	1.74282E-7	8.71407E-7	2.69932E-9	232	2600	2149	37554	over

GO:0007047	cell wall organization and biogenesis	2.31091E-7	1.21323E-6	3.72362E-9	63	446	2318	39708	over
GO:0045229	external encapsulating structure organization and biogenesis	2.87039E-7	1.57871E-6	4.66319E-9	63	449	2318	39705	over
GO:0042221	response to chemical stimulus	3.10759E-7	1.92569E-6	6.3989E-9	251	2896	2130	37258	over
GO:0006040	amino sugar metabolic process	3.10759E-7	2.40838E-6	7.77022E-9	14	24	2367	40130	over
GO:0006041	glucosamine metabolic process	3.10759E-7	2.40838E-6	7.77022E-9	14	24	2367	40130	over
GO:0006043	glucosamine catabolic process	3.10759E-7	2.40838E-6	7.77022E-9	14	24	2367	40130	over
GO:0046348	amino sugar catabolic process	3.10759E-7	2.40838E-6	7.77022E-9	14	24	2367	40130	over
GO:0006032	chitin catabolic process	3.10759E-7	2.40838E-6	7.77022E-9	14	24	2367	40130	over
GO:0006044	N-acetylglucosamine metabolic process	3.10759E-7	2.40838E-6	7.77022E-9	14	24	2367	40130	over
GO:0006030	chitin metabolic process	3.10759E-7	2.40838E-6	7.77022E-9	14	24	2367	40130	over
GO:0006046	N-acetylglucosamine catabolic process	3.10759E-7	2.40838E-6	7.77022E-9	14	24	2367	40130	over
GO:0047763	caffeate O-methyltransferase activity	3.36997E-7	2.69597E-6	8.22865E-9	9	5	2372	40149	over
GO:0016757	transferase activity, transferring glycosyl groups	3.82521E-7	3.1558E-6	1.01257E-8	109	1001	2272	39153	over
GO:0004568	chitinase activity	4.139E-7	3.51814E-6	1.14352E-8	14	25	2367	40129	over
GO:0009832	cellulose and pectin-containing cell wall biogenesis	5.90955E-7	5.29261E-6	1.62882E-8	32	155	2349	39999	over
GO:0009809	lignin biosynthetic process	5.90955E-7	5.31858E-6	1.64089E-8	24	90	2357	40064	over

GO:0008194	UDP-glycosyltransferase activity	8.65374E-7	8.00468E-6	2.43088E-8	65	494	2316	39660	over
GO:0020037	heme binding	1.88025E-6	1.78622E-5	5.16704E-8	43	269	2338	39885	over
GO:0006073	glucan metabolic process	3.1096E-6	3.03181E-5	9.05337E-8	55	402	2326	39752	over
GO:0046872	metal ion binding	3.60526E-6	3.6052E-5	1.02059E-7	346	4391	2035	35763	over
GO:0031226	intrinsic to plasma membrane	4.37991E-6	4.4893E-5	1.40046E-7	25	111	2356	40043	over
GO:0046527	glucosyltransferase activity	6.10933E-6	6.41459E-5	1.91453E-7	44	294	2337	39860	over
GO:0004497	monooxygenase activity	9.87111E-6	1.06109E-4	3.0119E-7	45	310	2336	39844	over
GO:0042546	cell wall biogenesis	1.095E-5	1.20443E-4	3.43307E-7	33	190	2348	39964	over
GO:0005506	iron ion binding	1.19011E-5	1.33878E-4	4.10672E-7	81	731	2300	39423	over
GO:0005983	starch catabolic process	2.22466E-5	2.55803E-4	6.87705E-7	10	16	2371	40138	over
GO:0030528	transcription regulator activity	2.5613E-5	3.00907E-4	7.94815E-7	146	1597	2235	38557	over
GO:0010044	response to aluminum ion	2.67321E-5	3.20734E-4	8.17557E-7	8	8	2373	40146	over
GO:0046906	tetrapyrrole binding	2.76285E-5	3.38392E-4	8.6278E-7	43	302	2338	39852	over
GO:0031988	membrane-bound vesicle	3.39063E-5	4.32212E-4	1.09288E-6	365	4796	2016	35358	over
GO:0031982	vesicle	3.39063E-5	4.32212E-4	1.09288E-6	365	4796	2016	35358	over
GO:0009664	cellulose and pectin-containing cell wall organization and biogenesis	3.56788E-5	4.63717E-4	1.21427E-6	41	285	2340	39869	over

GO:0035251	UDP-glucosyltransferase activity	3.77837E-5	5.09454E-4	1.38174E-6	40	276	2341	39878	over
GO:0031410	cytoplasmic vesicle	3.77837E-5	5.19391E-4	1.41869E-6	364	4795	2017	35359	over
GO:0016023	cytoplasmic membrane-bound vesicle	3.77837E-5	5.19391E-4	1.41869E-6	364	4795	2017	35359	over
GO:0016491	oxidoreductase activity	3.89936E-5	5.46826E-4	1.48494E-6	283	3583	2098	36571	over
GO:0008171	O-methyltransferase activity	3.89936E-5	5.56996E-4	1.50604E-6	15	48	2366	40106	over
GO:0009251	glucan catabolic process	3.89936E-5	5.71976E-4	1.53232E-6	10	18	2371	40136	over
GO:0008374	O-acyltransferase activity	3.89936E-5	5.74991E-4	1.53421E-6	18	70	2363	40084	over
GO:0005576	extracellular region	4.19726E-5	6.29391E-4	1.83059E-6	47	356	2334	39798	over
GO:0009830	cell wall modification during abscission	5.02253E-5	7.65643E-4	2.17154E-6	7	6	2374	40148	over
GO:0009725	response to hormone stimulus	5.58143E-5	8.64747E-4	2.2767E-6	134	1466	2247	38688	over
GO:0009986	cell surface	6.67799E-5	0.0010646	3.00877E-6	18	74	2363	40080	over
GO:0043565	sequence-specific DNA binding	6.67799E-5	0.00106791	3.0305E-6	45	341	2336	39813	over
GO:0010075	regulation of meristem size	7.73385E-5	0.00127527	3.16445E-6	10	20	2371	40134	over
GO:0035266	meristem growth	7.73385E-5	0.00127527	3.16445E-6	10	20	2371	40134	over
GO:0030151	molybdenum ion binding	9.72045E-5	0.00162685	4.13201E-6	7	7	2374	40147	over
GO:0010087	vascular tissue development (sensu Tracheophyta)	9.94298E-5	0.00168888	4.32389E-6	14	46	2367	40108	over

GO:0009607	response to biotic stimulus	1.09966E-4	0.00192205	5.20149E-6	107	1126	2274	39028	over
GO:0003700	transcription factor activity	1.09966E-4	0.00192256	5.20865E-6	112	1193	2269	38961	over
GO:0005984	disaccharide metabolic process	1.24774E-4	0.00221229	6.0468E-6	21	104	2360	40050	over
GO:0009531	secondary cell wall	1.59836E-4	0.00287292	7.37124E-6	7	8	2374	40146	over
GO:0009719	response to endogenous stimulus	1.75251E-4	0.00319322	8.56631E-6	178	2129	2203	38025	over
GO:0003796	lysozyme activity	2.53599E-4	0.00468059	1.04516E-5	5	2	2376	40152	over
GO:0048833	regulation of floral organ number	4.60475E-4	0.00871088	2.10714E-5	6	6	2375	40148	over
GO:0048832	regulation of organ number	4.60475E-4	0.00871088	2.10714E-5	6	6	2375	40148	over
GO:0003677	DNA binding	4.63606E-4	0.00892337	2.21835E-5	257	3331	2124	36823	over
GO:0010073	meristem maintenance	4.63606E-4	0.00908641	2.27302E-5	11	33	2370	40121	over
GO:0009699	phenylpropanoid biosynthetic process	4.63606E-4	0.00911444	2.28739E-5	37	281	2344	39873	over
GO:0035250	UDP-galactosyltransferase activity	5.0916E-4	0.0101315	2.59624E-5	9	21	2372	40133	over
GO:0046857	oxidoreductase activity, acting on other nitrogenous compounds as donors, with NAD or NADP as acceptor	5.17519E-4	0.0109657	2.6577E-5	5	3	2376	40151	over
GO:0004008	copper-exporting ATPase activity	5.17519E-4	0.0109657	2.6577E-5	5	3	2376	40151	over
GO:0009703	nitrate reductase (NADH) activity	5.17519E-4	0.0109657	2.6577E-5	5	3	2376	40151	over
GO:0043682	copper-transporting ATPase activity	5.17519E-4	0.0109657	2.6577E-5	5	3	2376	40151	over

GO:0005372	water transporter activity	5.17519E-4	0.011065	2.68454E-5	13	48	2368	40106	over
GO:0015250	water channel activity	5.17519E-4	0.011065	2.68454E-5	13	48	2368	40106	over
GO:0010382	cell wall metabolic process	5.28328E-4	0.0114254	2.85355E-5	15	64	2366	40090	over
GO:0046658	anchored to plasma membrane	5.83869E-4	0.012763	3.14048E-5	7	11	2374	40143	over
GO:0045551	cinnamyl-alcohol dehydrogenase activity	6.8333E-4	0.0150891	3.72712E-5	6	7	2375	40147	over
GO:0016157	sucrose synthase activity	7.6186E-4	0.0169958	4.42037E-5	11	36	2370	40118	over
GO:0048480	stigma development	8.7115E-4	0.0198371	4.67869E-5	4	1	2377	40153	over
GO:0048479	style development	8.7115E-4	0.0198371	4.67869E-5	4	1	2377	40153	over
GO:0015833	peptide transport	8.84234E-4	0.0205652	4.843E-5	16	76	2365	40078	over
GO:0006857	oligopeptide transport	8.84234E-4	0.0205652	4.843E-5	16	76	2365	40078	over
GO:0016760	cellulose synthase (UDP-forming) activity	8.87563E-4	0.0208591	4.99984E-5	17	85	2364	40069	over
GO:0016208	AMP binding	0.00111924	0.0265043	6.21256E-5	6	8	2375	40146	over
GO:0009415	response to water	0.0011327	0.0270942	6.44005E-5	46	403	2335	39751	over
GO:0009698	phenylpropanoid metabolic process	0.00126575	0.0305351	7.56948E-5	42	358	2339	39796	over
GO:0050879	multicellular organismal movement	0.00132469	0.032575	8.09913E-5	8	19	2373	40135	over
GO:0010031	circumnutation	0.00132469	0.032575	8.09913E-5	8	19	2373	40135	over

GO:0007389	pattern specification process	0.00134413	0.0333699	8.29726E-5	27	189	2354	39965	over
GO:0008378	galactosyltransferase activity	0.00139746	0.035008	8.89705E-5	13	55	2368	40099	over
GO:0016759	cellulose synthase activity	0.00140547	0.0356917	9.27928E-5	17	90	2364	40064	over
GO:0006950	response to stress	0.00140547	0.0358829	9.34821E-5	197	2512	2184	37642	over
GO:0032350	regulation of hormone metabolic process	0.00149241	0.0387774	9.86286E-5	6	9	2375	40145	over
GO:0010337	regulation of salicylic acid metabolic process	0.00149241	0.0387774	9.86286E-5	6	9	2375	40145	over
GO:0009897	external side of plasma membrane	0.00152251	0.0399575	9.88942E-5	7	14	2374	40140	over
GO:0005887	integral to plasma membrane	0.00152251	0.0409399	1.0058E-4	18	100	2363	40054	over
GO:0016684	oxidoreductase activity, acting on peroxide as acceptor	0.00152251	0.041005	1.01175E-4	33	258	2348	39896	over
GO:0004601	peroxidase activity	0.00152251	0.041005	1.01175E-4	33	258	2348	39896	over
GO:0016762	xyloglucan:xyloglucosyl transferase activity	0.00154972	0.0420934	1.04545E-4	15	73	2366	40081	over
GO:0048040	UDP-glucuronate decarboxylase activity	0.00158138	0.0433131	1.07837E-4	8	20	2373	40134	over
GO:0048582	positive regulation of post-embryonic development	0.00163948	0.0460417	1.08771E-4	5	5	2376	40149	over
GO:0048578	positive regulation of long-day photoperiodism, flowering	0.00163948	0.0460417	1.08771E-4	5	5	2376	40149	over
GO:0048584	positive regulation of response to stimulus	0.00163948	0.0460417	1.08771E-4	5	5	2376	40149	over
GO:0009414	response to water deprivation	0.00166323	0.0470893	1.17119E-4	43	378	2338	39776	over

GO:0046351	disaccharide biosynthetic process	0.00167616	0.0478456	1.19296E-4	15	74	2366	40080	over
GO:0019748	secondary metabolic process	0.00168934	0.0486147	1.22619E-4	74	775	2307	39379	over
GO:0009735	response to cytokinin stimulus	0.00176603	0.0511835	1.32519E-4	20	122	2361	40032	over
GO:0009934	regulation of meristem organization	0.002033	0.059168	1.38044E-4	7	15	2374	40139	over
GO:0008430	selenium binding	0.00221285	0.0647483	1.50325E-4	6	10	2375	40144	over
GO:0030794	(S)-coclaurine-N-methyltransferase activity	0.00265207	0.0789266	1.75195E-4	3	0	2378	40154	over
GO:0033273	response to vitamin	0.00265207	0.0789266	1.75195E-4	3	0	2378	40154	over
GO:0010266	response to vitamin B1	0.00265207	0.0789266	1.75195E-4	3	0	2378	40154	over
GO:0004029	aldehyde dehydrogenase (NAD) activity	0.0026775	0.0821262	1.83694E-4	8	22	2373	40132	over
GO:0009838	abscission	0.0026775	0.0821262	1.83694E-4	8	22	2373	40132	over
GO:0009933	meristem organization	0.0026775	0.0832889	1.8856E-4	13	60	2368	40094	over
GO:0043067	regulation of programmed cell death	0.0026775	0.0832889	1.8856E-4	13	60	2368	40094	over
GO:0048532	organization of an anatomical structure	0.0026775	0.0832889	1.8856E-4	13	60	2368	40094	over
GO:0009739	response to gibberellin stimulus	0.0026775	0.0833413	1.88647E-4	25	178	2356	39976	over
GO:0048046	apoplast	0.0028234	0.0888326	1.94325E-4	20	126	2361	40028	over
GO:0005982	starch metabolic process	0.0028234	0.0889648	1.94684E-4	19	116	2362	40038	over

GO:0016998	cell wall catabolic process	0.0029142	0.092352	2.01029E-4	11	44	2370	40110	over
GO:0005975	carbohydrate metabolic process	0.0029616	0.0944524	2.1636E-4	173	2199	2208	37955	over
GO:0046556	alpha-N-arabinofuranosidase activity	0.00334085	0.107177	2.53791E-4	7	17	2374	40137	over
GO:0006118	electron transport	0.00334085	0.107377	2.53997E-4	123	1479	2258	38675	over
GO:0005886	plasma membrane	0.00359161	0.11575	2.96841E-4	73	787	2308	39367	over
GO:0042732	D-xylose metabolic process	0.00363978	0.118011	2.98746E-4	9	31	2372	40123	over
GO:0008506	sucrose:hydrogen symporter activity	0.0038855	0.126305	2.98957E-4	4	3	2377	40151	over
GO:0040008	regulation of growth	0.00436966	0.141821	3.2669E-4	10	39	2371	40115	over
GO:0016101	diterpenoid metabolic process	0.0044332	0.145623	3.35521E-4	7	18	2374	40136	over
GO:0009685	gibberellin metabolic process	0.0044332	0.145623	3.35521E-4	7	18	2374	40136	over
GO:0019825	oxygen binding	0.00473126	0.155616	3.75015E-4	8	25	2373	40129	over
GO:0006979	response to oxidative stress	0.00473302	0.156667	3.82187E-4	54	542	2327	39612	over
GO:0048509	regulation of meristem development	0.00479997	0.159707	3.88063E-4	10	40	2371	40114	over
GO:0010080	regulation of floral meristem size	0.00583656	0.191878	4.82077E-4	5	8	2376	40146	over
GO:0008270	zinc ion binding	0.00589067	0.19466	5.02342E-4	126	1551	2255	38603	over
GO:0032502	developmental process	0.00615628	0.20371	5.46186E-4	210	2802	2171	37352	over

GO:0016661	oxidoreductase activity, acting on other nitrogenous compounds as donors	0.00632043	0.209782	5.61677E-4	7	20	2374	40134	over
GO:0048038	quinone binding	0.00717079	0.235794	5.99468E-4	6	14	2375	40140	over
GO:0009733	response to auxin stimulus	0.00750888	0.246838	6.50925E-4	62	662	2319	39492	over
GO:0010327	acetyl CoA:(Z)-3-hexen-1-ol acetyltransferase activity	0.00851154	0.276357	6.71395E-4	3	1	2378	40153	over
GO:0009011	starch synthase activity	0.00872895	0.288549	7.12957E-4	7	21	2374	40133	over
GO:0010215	cellulose microfibril organization	0.00872895	0.288549	7.12957E-4	7	21	2374	40133	over
GO:0009827	cellulose and pectin-containing cell wall modification	0.00872895	0.288549	7.12957E-4	7	21	2374	40133	over
GO:0048196	middle lamella-containing extracellular matrix	0.00872895	0.288549	7.12957E-4	7	21	2374	40133	over
GO:0009653	anatomical structure morphogenesis	0.00985514	0.3208	8.06779E-4	84	971	2297	39183	over
GO:0006833	water transport	0.0100238	0.328655	8.5414E-4	8	29	2373	40125	over
GO:0042044	fluid transport	0.0100238	0.328655	8.5414E-4	8	29	2373	40125	over
GO:0005985	sucrose metabolic process	0.0107052	0.350086	9.68651E-4	13	73	2368	40081	over
GO:0042545	cell wall modification	0.0107052	0.350086	9.68651E-4	13	73	2368	40081	over
GO:0009044	xylan 1,4-beta-xylosidase activity	0.0112075	0.366655	9.82786E-4	4	5	2377	40149	over
GO:0016559	peroxisome fission	0.0112075	0.366655	9.82786E-4	4	5	2377	40149	over
GO:0009501	amyloplast	0.0125173	0.401457	0.00108138	13	74	2368	40080	over

GO:0019438	aromatic compound biosynthetic process	0.0132436	0.420941	0.00118475	39	376	2342	39778	over
GO:0005991	trehalose metabolic process	0.0136747	0.434753	0.00123215	8	31	2373	40123	over
GO:0031224	intrinsic to membrane	0.0136747	0.436346	0.00125273	148	1918	2233	38236	over
GO:0009734	auxin mediated signaling pathway	0.0136747	0.436953	0.00125703	22	171	2359	39983	over
GO:0051704	multi-organism process	0.0139146	0.444537	0.00131494	84	989	2297	39165	over
GO:0042398	amino acid derivative biosynthetic process	0.0143439	0.456475	0.00137741	45	457	2336	39697	over
GO:0009931	calcium-dependent protein serine/threonine kinase activity	0.0149408	0.47207	0.00142078	5	11	2376	40143	over
GO:0030244	cellulose biosynthetic process	0.0154616	0.485688	0.00149641	20	151	2361	40003	over
GO:0015827	tryptophan transport	0.0165332	0.512899	0.00156546	4	6	2377	40148	over
GO:0015801	aromatic amino acid transport	0.0165332	0.512899	0.00156546	4	6	2377	40148	over
GO:0032441	pheophorbide a oxygenase activity	0.0181017	0.547086	0.00160831	3	2	2378	40152	over
GO:0016131	brassinosteroid metabolic process	0.018553	0.560045	0.00166555	7	25	2374	40129	over
GO:0016128	phytosteroid metabolic process	0.018553	0.560045	0.00166555	7	25	2374	40129	over
GO:0009696	salicylic acid metabolic process	0.0189054	0.572129	0.0017167	6	18	2375	40136	over
GO:0030243	cellulose metabolic process	0.0189054	0.572666	0.00172239	20	153	2361	40001	over
GO:0048507	meristem development	0.0189054	0.573399	0.00173229	17	120	2364	40034	over

GO:0022829	wide pore channel activity	0.0189054	0.577107	0.00174978	18	131	2363	40023	over
GO:0015288	porin activity	0.0189054	0.577107	0.00174978	18	131	2363	40023	over
GO:0016209	antioxidant activity	0.0189054	0.578969	0.00176727	33	309	2348	39845	over
GO:0006725	aromatic compound metabolic process	0.0200286	0.602063	0.00195299	61	682	2320	39472	over
GO:0016020	membrane	0.0200647	0.60585	0.00200629	624	9468	1757	30686	over
GO:0005750	mitochondrial respiratory chain complex III	0.0200647	0.610618	0.00201301	7	26	2374	40128	over
GO:0006787	porphyrin catabolic process	0.0200647	0.610618	0.00201301	7	26	2374	40128	over
GO:0033015	tetrapyrrole catabolic process	0.0200647	0.610618	0.00201301	7	26	2374	40128	over
GO:0048856	anatomical structure development	0.0215081	0.638118	0.00224627	158	2100	2223	38054	over
GO:0005385	zinc ion transmembrane transporter activity	0.0223181	0.659401	0.00235133	4	7	2377	40147	over
GO:0045487	gibberellin catabolic process	0.0223181	0.659401	0.00235133	4	7	2377	40147	over
GO:0016103	diterpenoid catabolic process	0.0223181	0.659401	0.00235133	4	7	2377	40147	over
GO:0006829	zinc ion transport	0.0223181	0.659401	0.00235133	4	7	2377	40147	over
GO:0009269	response to desiccation	0.0233281	0.67859	0.00251665	9	44	2372	40110	over
GO:0003002	regionalization	0.0233281	0.679371	0.00252654	18	136	2363	40018	over
GO:0030258	lipid modification	0.0237755	0.689999	0.00253758	5	13	2376	40141	over

GO:0030259	lipid glycosylation	0.0237755	0.689999	0.00253758	5	13	2376	40141	over
GO:0005992	trehalose biosynthetic process	0.0261939	0.726621	0.00287362	7	28	2374	40126	over
GO:0004411	homogentisate 1,2-dioxygenase activity	0.0333023	0.809335	0.00313223	2	0	2379	40154	over
GO:0009755	hormone-mediated signaling	0.0336351	0.814039	0.00321595	43	453	2338	39701	over
GO:0004301	epoxide hydrolase activity	0.0337124	0.823896	0.0032693	6	21	2375	40133	over
GO:0046149	pigment catabolic process	0.0337124	0.823896	0.0032693	6	21	2375	40133	over
GO:0016803	ether hydrolase activity	0.0337124	0.823896	0.0032693	6	21	2375	40133	over
GO:0005615	extracellular space	0.0337124	0.823896	0.0032693	6	21	2375	40133	over
GO:0004463	leukotriene-A4 hydrolase activity	0.0337124	0.823896	0.0032693	6	21	2375	40133	over
GO:0015996	chlorophyll catabolic process	0.0337124	0.823896	0.0032693	6	21	2375	40133	over
GO:0051707	response to other organism	0.0374589	0.856166	0.00363864	72	858	2309	39296	over
GO:0048571	long-day photoperiodism	0.0374802	0.858994	0.00369348	8	38	2373	40116	over
GO:0048574	long-day photoperiodism, flowering	0.0374802	0.858994	0.00369348	8	38	2373	40116	over
GO:0009888	tissue development	0.0374875	0.860363	0.00374155	39	404	2342	39750	over
GO:0010118	stomatal movement	0.0376426	0.864583	0.00393612	19	154	2362	40000	over
GO:0048438	floral whorl development	0.0376426	0.864583	0.00393612	19	154	2362	40000	over

GO:0006664	glycolipid metabolic process	0.0376426	0.869106	0.00396649	6	22	2375	40132	over
GO:0009247	glycolipid biosynthetic process	0.0376426	0.869106	0.00396649	6	22	2375	40132	over
GO:0009687	abscisic acid metabolic process	0.0376426	0.869106	0.00396649	6	22	2375	40132	over
GO:0043288	apocarotenoid metabolic process	0.0376426	0.869106	0.00396649	6	22	2375	40132	over
GO:0015175	neutral amino acid transmembrane transporter activity	0.0378996	0.872129	0.00399082	7	30	2374	40124	over
GO:0015020	glucuronosyltransferase activity	0.0383541	0.882297	0.00418228	5	15	2376	40139	over
GO:0050734	hydroxycinnamoyltransferase activity	0.0383541	0.882297	0.00418228	5	15	2376	40139	over
GO:0001509	legumain activity	0.0383541	0.882297	0.00418228	5	15	2376	40139	over
GO:0047205	quinate O-hydroxycinnamoyltransferase activity	0.0383541	0.882297	0.00418228	5	15	2376	40139	over
GO:0008422	beta-glucosidase activity	0.0383541	0.882297	0.00418228	5	15	2376	40139	over
GO:0050737	O-hydroxycinnamoyltransferase activity	0.0383541	0.882297	0.00418228	5	15	2376	40139	over
GO:0004022	alcohol dehydrogenase activity	0.0383541	0.883349	0.00418932	13	88	2368	40066	over
GO:0044459	plasma membrane part	0.0386785	0.887201	0.00430506	34	342	2347	39812	over
GO:0006575	amino acid derivative metabolic process	0.0386785	0.887646	0.00433353	52	584	2329	39570	over
GO:0009250	glucan biosynthetic process	0.0388097	0.889554	0.00444846	31	304	2350	39850	over
GO:0004672	protein kinase activity	0.0391987	0.893022	0.0046418	153	2068	2228	38086	over

GO:0004373	glycogen (starch) synthase activity	0.0403495	0.902807	0.00465568	4	9	2377	40145	over
GO:0016174	NAD(P)H oxidase activity	0.0403495	0.902807	0.00465568	4	9	2377	40145	over
GO:0005403	hydrogen:sugar symporter activity	0.0403495	0.902807	0.00465568	4	9	2377	40145	over
GO:0031225	anchored to membrane	0.0405375	0.905788	0.00465954	7	31	2374	40123	over
GO:0015926	glucosidase activity	0.0405375	0.905788	0.00465954	7	31	2374	40123	over
GO:0016021	integral to membrane	0.0413012	0.911511	0.00477989	141	1888	2240	38266	over
GO:0005778	peroxisomal membrane	0.0413012	0.913538	0.00483909	8	40	2373	40114	over
GO:0031903	microbody membrane	0.0413012	0.913538	0.00483909	8	40	2373	40114	over
GO:0009877	nodulation	0.0413012	0.913538	0.00483909	8	40	2373	40114	over
GO:0003993	acid phosphatase activity	0.0414247	0.915055	0.00490591	11	69	2370	40085	over
GO:0016311	dephosphorylation	0.0418267	0.917928	0.00507442	27	256	2354	39898	over
GO:0004295	trypsin activity	0.0457219	0.93572	0.00523933	5	16	2376	40138	over
GO:0031012	extracellular matrix	0.0469533	0.941163	0.00540928	7	32	2374	40122	over
GO:0051789	response to protein stimulus	0.0469533	0.94237	0.00547101	26	245	2355	39909	over
GO:0006986	response to unfolded protein	0.0469533	0.94237	0.00547101	26	245	2355	39909	over
GO:0006855	multidrug transport	0.0483343	0.948278	0.00568198	6	24	2375	40130	over

GO:0046915	transition metal ion transmembrane transporter activity	0.0483343	0.948278	0.00568198	6	24	2375	40130	over

GOSSIP
Test-Set: tm1.20.25.up.txt
Tests for all terms in Gene Ontology whether it is enriched in a test group when compared to a reference group using Fisher's excact test with Multiple Testing.
Pub: Biological Profiling of Gene Groups utilizing Gene Ontology A Statistical Framework
Poster: GOSSIP: Biological Profiling of Gene Groups utilizing Gene Ontology
by Nils Blthgen, Karsten Brand, Hanspeter Herzel, Dieter Beule

GO Term	Name	FDR	FWER	single test p-Value	# in test group	# in reference group	# non annot test	# non annot reference group	Over/Under
GO:0006575	amino acid derivative metabolic process	6.99946E-9	3.52028E-8	0.0	50	586	570	41329	over
GO:0000038	very-long-chain fatty acid metabolic process	6.99946E-9	3.52028E-8	0.0	15	35	605	41880	over
GO:0005576	extracellular region	6.99946E-9	3.52028E-8	0.0	47	356	573	41559	over
GO:0008415	acyltransferase activity	6.99946E-9	3.52028E-8	0.0	40	546	580	41369	over
GO:0009813	flavonoid biosynthetic process	6.99946E-9	3.52028E-8	0.0	29	146	591	41769	over
GO:0009812	flavonoid metabolic process	6.99946E-9	3.52028E-8	0.0	30	161	590	41754	over
GO:0019438	aromatic compound biosynthetic process	6.99946E-9	3.52028E-8	0.0	38	377	582	41538	over
GO:0019208	phosphatase regulator activity	6.99946E-9	3.52028E-8	0.0	15	66	605	41849	over

GO:0030570	pectate lyase activity	6.99946E-9	3.52028E-8	0.0	15	47	605	41868	over
GO:0009699	phenylpropanoid biosynthetic process	6.99946E-9	3.52028E-8	0.0	37	281	583	41634	over
GO:0019748	secondary metabolic process	6.99946E-9	3.52028E-8	0.0	53	796	567	41119	over
GO:0008601	protein phosphatase type 2A regulator activity	6.99946E-9	3.52028E-8	0.0	15	64	605	41851	over
GO:0016746	transferase activity, transferring acyl groups	6.99946E-9	3.52028E-8	0.0	40	619	580	41296	over
GO:0042335	cuticle development	6.99946E-9	3.52028E-8	0.0	13	36	607	41879	over
GO:0019888	protein phosphatase regulator activity	6.99946E-9	3.52028E-8	0.0	15	66	605	41849	over
GO:0009698	phenylpropanoid metabolic process	6.99946E-9	3.52028E-8	0.0	48	352	572	41563	over
GO:0016837	carbon-oxygen lyase activity, acting on polysaccharides	6.99946E-9	3.52028E-8	0.0	15	47	605	41868	over
GO:0016705	oxidoreductase activity, acting on paired donors, with incorporation or reduction of molecular oxygen	6.99946E-9	3.52028E-8	0.0	28	321	592	41594	over
GO:0042398	amino acid derivative biosynthetic process	6.99946E-9	3.84501E-8	2.04344E- 11	38	464	582	41451	over
GO:0016747	transferase activity, transferring groups other than amino-acyl groups	6.99946E-9	3.89917E-8	2.50049E- 11	40	556	580	41359	over
GO:0012505	endomembrane system	6.99946E-9	3.98636E-8	2.81299E- 11	80	2318	540	39597	over
GO:0006725	aromatic compound metabolic process	6.99946E-9	4.02477E-8	3.07474E- 11	50	693	570	41222	over

GO:0031418	L-ascorbic acid binding	6.99946E-9	4.14813E-8	3.96724E-11	13	40	607	41875	over
GO:0045486	naringenin 3-dioxygenase activity	6.99946E-9	4.34717E-8	4.60621E-11	9	15	611	41900	over
GO:0006631	fatty acid metabolic process	6.99946E-9	4.37466E-8	4.8877E-11	33	547	587	41368	over
GO:0045552	dihydrokaempferol 4-reductase activity	1.84644E-7	1.20018E-6	3.82057E-9	5	1	615	41914	over
GO:0045548	phenylalanine ammonia-lyase activity	2.58773E-7	1.91753E-6	6.40035E-9	8	21	612	41894	over
GO:0031410	cytoplasmic vesicle	2.58773E-7	1.98976E-6	6.78396E-9	125	5034	495	36881	over
GO:0016023	cytoplasmic membrane-bound vesicle	2.58773E-7	1.98976E-6	6.78396E-9	125	5034	495	36881	over
GO:0031982	vesicle	2.58773E-7	2.00549E-6	6.9835E-9	125	5036	495	36879	over
GO:0031988	membrane-bound vesicle	2.58773E-7	2.00549E-6	6.9835E-9	125	5036	495	36879	over
GO:0030234	enzyme regulator activity	7.8147E-7	6.25174E-6	2.28067E-8	24	400	596	41515	over
GO:0006559	L-phenylalanine catabolic process	8.27352E-7	6.82563E-6	2.5352E-8	8	26	612	41889	over
GO:0006519	amino acid and derivative metabolic process	9.36033E-7	7.95625E-6	2.85823E-8	55	1628	565	40287	over
GO:0009288	flagellin-based flagellum	1.41681E-6	1.27512E-5	4.19114E-8	6	9	614	41906	over
GO:0001539	ciliary or flagellar motility	1.41681E-6	1.27512E-5	4.19114E-8	6	9	614	41906	over
GO:0003824	catalytic activity	1.74339E-6	1.61262E-5	4.79935E-8	336	18173	284	23742	over

GO:0016798	hydrolase activity, acting on glycosyl bonds	1.9694E-6	1.87092E-5	5.98934E-8	35	819	585	41096	over
GO:0009074	aromatic amino acid family catabolic process	1.9797E-6	1.97968E-5	6.47855E-8	8	30	612	41885	over
GO:0007267	cell-cell signaling	1.9797E-6	1.97968E-5	6.47855E-8	8	30	612	41885	over
GO:0016841	ammonia-lyase activity	2.60179E-6	2.6668E-5	8.04607E-8	8	31	612	41884	over
GO:0006928	cell motility	2.9019E-6	3.24945E-5	1.01054E-7	6	11	614	41904	over
GO:0051674	localization of cell	2.9019E-6	3.24945E-5	1.01054E-7	6	11	614	41904	over
GO:0004553	hydrolase activity, hydrolyzing O-glycosyl compounds	2.9019E-6	3.25118E-5	1.0114E-7	33	760	587	41155	over
GO:0016706	oxidoreductase activity, acting on paired donors, with incorporation or reduction of molecular oxygen, 2-oxoglutarate as one donor, and incorporation of one atom each of oxygen into both donors	2.9019E-6	3.26458E-5	1.0216E-7	13	122	607	41793	over
GO:0032787	monocarboxylic acid metabolic process	3.25751E-6	3.74607E-5	1.31157E-7	34	808	586	41107	over
GO:0042995	cell projection	3.6058E-6	4.32687E-5	1.49726E-7	6	12	614	41903	over
GO:0019861	flagellum	3.6058E-6	4.32687E-5	1.49726E-7	6	12	614	41903	over
GO:0016210	naringenin-chalcone synthase activity	4.90756E-6	6.01158E-5	2.05638E-7	7	23	613	41892	over
GO:0019439	aromatic compound catabolic process	5.24585E-6	6.55709E-5	2.17455E-7	8	36	612	41879	over
GO:0016840	carbon-nitrogen lyase activity	8.99519E-6	1.14682E-4	3.78332E-7	9	55	611	41860	over
GO:0005506	iron ion binding	1.23539E-5	1.60588E-4	5.21848E-7	32	780	588	41135	over

GO:0006558	L-phenylalanine metabolic process	2.57203E-5	3.40736E-4	1.12361E-6	8	46	612	41869	over
GO:0010025	wax biosynthetic process	4.38083E-5	6.24073E-4	1.96686E-6	8	50	612	41865	over
GO:0010166	wax metabolic process	4.38083E-5	6.24073E-4	1.96686E-6	8	50	612	41865	over
GO:0016211	ammonia ligase activity	4.38083E-5	6.24073E-4	1.96686E-6	8	50	612	41865	over
GO:0016880	acid-ammonia (or amide) ligase activity	4.38083E-5	6.24073E-4	1.96686E-6	8	50	612	41865	over
GO:0051213	dioxygenase activity	6.42786E-5	9.63714E-4	2.98743E-6	4	4	616	41911	over
GO:0050589	leucocyanidin oxygenase activity	6.42786E-5	9.63714E-4	2.98743E-6	4	4	616	41911	over
GO:0009924	octadecanal decarbonylase activity	6.42786E-5	9.63714E-4	2.98743E-6	4	4	616	41911	over
GO:0030599	pectinesterase activity	7.69904E-5	0.00117341	3.25656E-6	11	118	609	41797	over
GO:0016717	oxidoreductase activity, acting on paired donors, with oxidation of a pair of donors resulting in the reduction of molecular oxygen to two molecules of water	8.45519E-5	0.00132515	3.72819E-6	8	55	612	41860	over
GO:0016491	oxidoreductase activity	8.45519E-5	0.00133081	3.77414E-6	91	3775	529	38140	over
GO:0016829	lyase activity	9.39614E-5	0.00150225	4.35722E-6	39	1171	581	40744	over
GO:0010039	response to iron ion	1.82261E-4	0.00295736	8.75621E-6	4	6	616	41909	over
GO:0048029	monosaccharide binding	2.12177E-4	0.00354766	1.08596E-5	5	16	615	41899	over
GO:0048032	galacturonate binding	2.12177E-4	0.00354766	1.08596E-5	5	16	615	41899	over
GO:0004650	polygalacturonase activity	5.13616E-4	0.00869348	2.69297E-5	8	74	612	41841	over

GO:0051049	regulation of transport	5.59502E-4	0.00974353	2.87919E-5	4	9	616	41906	over
GO:0043269	regulation of ion transport	5.59502E-4	0.00974353	2.87919E-5	4	9	616	41906	over
GO:0016020	membrane	5.59538E-4	0.00988265	2.97391E-5	191	9901	429	32014	over
GO:0019842	vitamin binding	7.09386E-4	0.0128579	3.72398E-5	16	317	604	41598	over
GO:0006629	lipid metabolic process	7.09386E-4	0.0128629	3.7282E-5	41	1388	579	40527	over
GO:0004091	carboxylesterase activity	9.14302E-4	0.0167724	5.07776E-5	17	362	603	41553	over
GO:0035173	histone kinase activity	0.00116335	0.0224301	5.96554E-5	3	3	617	41912	over
GO:0016572	histone phosphorylation	0.00116335	0.0224301	5.96554E-5	3	3	617	41912	over
GO:0035174	histone serine kinase activity	0.00116335	0.0224301	5.96554E-5	3	3	617	41912	over
GO:0035175	histone serine kinase activity (H3-S10 specific)	0.00116335	0.0224301	5.96554E-5	3	3	617	41912	over
GO:0006633	fatty acid biosynthetic process	0.00129225	0.025199	6.84595E-5	18	409	602	41506	over
GO:0009411	response to UV	0.00135459	0.0268087	7.18373E-5	10	139	610	41776	over
GO:0007047	cell wall organization and biogenesis	0.00135459	0.0270578	7.27795E-5	20	489	600	41426	over
GO:0045229	external encapsulating structure organization and biogenesis	0.00139177	0.0281283	7.88096E-5	20	492	600	41423	over
GO:0005199	structural constituent of cell wall	0.00147625	0.030168	8.62919E-5	6	44	614	41871	over
GO:0016053	organic acid biosynthetic process	0.00224958	0.0466794	1.43243E-4	18	435	602	41480	over

GO:0046394	carboxylic acid biosynthetic process	0.00224958	0.0466794	1.43243E-4	18	435	602	41480	over
GO:0000254	C-4 methylsterol oxidase activity	0.00271835	0.0567701	1.63429E-4	3	5	617	41910	over
GO:0050734	hydroxycinnamoyltransferase activity	0.00288142	0.0621005	1.799E-4	4	16	616	41899	over
GO:0050737	O-hydroxycinnamoyltransferase activity	0.00288142	0.0621005	1.799E-4	4	16	616	41899	over
GO:0047205	quinate O-hydroxycinnamoyltransferase activity	0.00288142	0.0621005	1.799E-4	4	16	616	41899	over
GO:0044426	cell wall part	0.00349759	0.0781017	2.12129E-4	2	0	618	41915	over
GO:0048226	Casparian strip	0.00349759	0.0781017	2.12129E-4	2	0	618	41915	over
GO:0015115	silicate transmembrane transporter activity	0.00349759	0.0781017	2.12129E-4	2	0	618	41915	over
GO:0015708	silicate transport	0.00349759	0.0781017	2.12129E-4	2	0	618	41915	over
GO:0010224	response to UV-B	0.00381375	0.0857259	2.32119E-4	7	77	613	41838	over
GO:0048046	apoplast	0.00486203	0.109058	3.09189E-4	9	137	611	41778	over
GO:0016724	oxidoreductase activity, oxidizing metal ions, oxygen as acceptor	0.00546191	0.124057	3.42656E-4	3	7	617	41908	over
GO:0004322	ferroxidase activity	0.00546191	0.124057	3.42656E-4	3	7	617	41908	over
GO:0006869	lipid transport	0.00729386	0.163648	4.9415E-4	10	179	610	41736	over
GO:0008393	fatty acid (omega-1)-hydroxylase activity	0.0091769	0.205016	6.1468E-4	3	9	617	41906	over
GO:0004351	glutamate decarboxylase activity	0.0091769	0.205016	6.1468E-4	3	9	617	41906	over

GO:0045430	chalcone isomerase activity	0.0104006	0.230973	6.30222E-4	2	1	618	41914	over
GO:0009505	cellulose and pectin-containing cell wall	0.0105338	0.237068	6.67047E-4	13	295	607	41620	over
GO:0009072	aromatic amino acid family metabolic process	0.0105338	0.237581	6.67927E-4	8	122	612	41793	over
GO:0008199	ferric iron binding	0.0119889	0.269726	7.90446E-4	3	10	617	41905	over
GO:0016835	carbon-oxygen lyase activity	0.0119889	0.270012	7.92882E-4	15	380	605	41535	over
GO:0004497	monooxygenase activity	0.012239	0.280551	8.16522E-4	14	341	606	41574	over
GO:0005529	sugar binding	0.012239	0.280792	8.18675E-4	9	158	611	41757	over
GO:0044255	cellular lipid metabolic process	0.012239	0.281416	8.29004E-4	33	1212	587	40703	over
GO:0030246	carbohydrate binding	0.0143296	0.323288	9.97666E-4	11	233	609	41682	over
GO:0009664	cellulose and pectin-containing cell wall organization and biogenesis	0.0152283	0.342172	0.00111926	13	313	607	41602	over
GO:0006826	iron ion transport	0.0167176	0.373831	0.00123052	3	12	617	41903	over
GO:0000775	chromosome, pericentric region	0.0167176	0.373831	0.00123052	3	12	617	41903	over
GO:0010321	regulation of vegetative phase change	0.0184599	0.419939	0.00124825	2	2	618	41913	over
GO:0033559	unsaturated fatty acid metabolic process	0.0184599	0.419939	0.00124825	2	2	618	41913	over
GO:0030337	DNA polymerase processivity factor activity	0.0184599	0.419939	0.00124825	2	2	618	41913	over
GO:0006636	unsaturated fatty acid biosynthetic process	0.0184599	0.419939	0.00124825	2	2	618	41913	over

GO:0042389	omega-3 fatty acid desaturase activity	0.0184599	0.419939	0.00124825	2	2	618	41913	over
GO:0043626	PCNA complex	0.0184599	0.419939	0.00124825	2	2	618	41913	over
GO:0019752	carboxylic acid metabolic process	0.0191184	0.436563	0.0013817	49	2094	571	39821	over
GO:0006082	organic acid metabolic process	0.0191184	0.437868	0.00139496	49	2095	571	39820	over
GO:0005618	cell wall	0.0191184	0.439201	0.00142265	15	404	605	41511	over
GO:0005507	copper ion binding	0.0243075	0.523592	0.00191248	10	216	610	41699	over
GO:0006275	regulation of DNA replication	0.0279238	0.576326	0.00206031	2	3	618	41912	over
GO:0030312	external encapsulating structure	0.0282204	0.583126	0.00213315	15	422	605	41493	over
GO:0009734	auxin mediated signaling pathway	0.0298812	0.607	0.00222948	9	184	611	41731	over
GO:0004675	transmembrane receptor protein serine/threonine kinase activity	0.0328836	0.645139	0.00250948	3	16	617	41899	over
GO:0008289	lipid binding	0.0343743	0.664323	0.00281198	15	435	605	41480	over
GO:0043169	cation binding	0.0423035	0.741805	0.00320637	76	3729	544	38186	over
GO:0016722	oxidoreductase activity, oxidizing metal ions	0.049396	0.796783	0.00386105	3	19	617	41896	over

GOSSIP
Test-Set: tm1.20.25.down.txt
Tests for all terms in Gene Ontology whether it is enriched in a test group when compared to a reference group using Fisher's excact test with Multiple Testing.
Pub: Biological Profiling of Gene Groups utilizing Gene Ontology A Statistical Framework
Poster: GOSSIP: Biological Profiling of Gene Groups utilizing Gene Ontology

by Nils Blthgen, Karsten Brand, Hanspeter Herzel, Dieter Beule									
GO Term	Name	FDR	FWER	single test p-Value	# in test group	# in reference group	# non annot test	# non annot reference group	Over/Under
GO:0020037	heme binding	8.0732E-7	5.24747E-7	1.9506E-9	18	294	391	41832	over
GO:0045735	nutrient reservoir activity	8.0732E-7	5.27037E-7	2.01138E-9	12	107	397	42019	over
GO:0009620	response to fungus	8.0732E-7	6.0549E-7	2.14377E-9	16	225	393	41901	over
GO:0009703	nitrate reductase (NADH) activity	9.98239E-7	1.2478E-6	4.4358E-9	5	3	404	42123	over
GO:0046857	oxidoreductase activity, acting on other nitrogenous compounds as donors, with NAD or NADP as acceptor	9.98239E-7	1.2478E-6	4.4358E-9	5	3	404	42123	over
GO:0050832	defense response to fungus	1.05668E-6	1.58502E-6	5.59615E-9	13	146	396	41980	over
GO:0046906	tetrapyrrole binding	1.56376E-6	2.73658E-6	9.3189E-9	18	327	391	41799	over
GO:0030151	molybdenum ion binding	2.38002E-5	4.75993E-5	1.49575E-7	5	9	404	42117	over
GO:0016661	oxidoreductase activity, acting on other nitrogenous compounds as donors	2.55016E-5	5.7377E-5	1.90171E-7	6	21	403	42105	over
GO:0016491	oxidoreductase activity	9.41473E-5	2.35341E-4	8.62753E-7	68	3798	341	38328	over
GO:0006118	electron transport	1.25666E-4	3.45522E-4	1.0292E-6	37	1565	372	40561	over
GO:0031347	regulation of defense response	1.28116E-4	3.84273E-4	1.16301E-6	6	30	403	42096	over

GO:0048583	regulation of response to stimulus	1.31541E-4	4.27415E-4	1.42336E-6	7	51	402	42075	over
GO:0009751	response to salicylic acid stimulus	3.95877E-4	0.00138461	4.18752E-6	13	269	396	41857	over
GO:0009617	response to bacterium	5.28414E-4	0.00197959	6.37408E-6	14	325	395	41801	over
GO:0051704	multi-organism process	5.32556E-4	0.00212796	6.88152E-6	27	1046	382	41080	over
GO:0004497	monooxygenase activity	8.72627E-4	0.0037018	1.07204E-5	14	341	395	41785	over
GO:0003959	NADPH dehydrogenase activity	9.08511E-4	0.00454042	1.39915E-5	4	12	405	42114	over
GO:0008430	selenium binding	9.08511E-4	0.00454042	1.39915E-5	4	12	405	42114	over
GO:0051707	response to other organism	9.08511E-4	0.0047163	1.489E-5	24	906	385	41220	over
GO:0042742	defense response to bacterium	9.08511E-4	0.00490818	1.56203E-5	11	218	398	41908	over
GO:0005506	iron ion binding	9.08511E-4	0.00498435	1.62292E-5	22	790	387	41336	over
GO:0004324	ferredoxin-NADP+ reductase activity	0.00110905	0.00635676	1.81577E-5	4	13	405	42113	over
GO:0050896	response to stimulus	0.00115883	0.0069289	2.13197E-5	86	5692	323	36434	over
GO:0030528	transcription regulator activity	0.00204798	0.0127183	3.92011E-5	35	1708	374	40418	over
GO:0050660	FAD binding	0.00206126	0.0133089	4.24202E-5	10	201	399	41925	over
GO:0030007	cellular potassium ion homeostasis	0.0023116	0.016051	4.76836E-5	3	5	406	42121	over
GO:0055075	potassium ion homeostasis	0.0023116	0.016051	4.76836E-5	3	5	406	42121	over

GO:0016731	oxidoreductase activity, acting on iron-sulfur proteins as donors, NAD or NADP as acceptor	0.00265027	0.0196808	6.45417E-5	4	19	405	42107	over
GO:0008937	ferredoxin reductase activity	0.00265027	0.0196808	6.45417E-5	4	19	405	42107	over
GO:0055067	monovalent inorganic cation homeostasis	0.00281962	0.0223046	7.10147E-5	3	6	406	42120	over
GO:0030004	cellular monovalent inorganic cation homeostasis	0.00281962	0.0223046	7.10147E-5	3	6	406	42120	over
GO:0003700	transcription factor activity	0.00793752	0.0633873	1.88833E-4	27	1278	382	40848	over
GO:0004672	protein kinase activity	0.00951896	0.0777257	2.38658E-4	39	2182	370	39944	over
GO:0010286	heat acclimation	0.012535	0.106686	2.96911E-4	3	11	406	42115	over
GO:0000234	phosphoethanolamine N-methyltransferase activity	0.012535	0.106686	2.96911E-4	3	11	406	42115	over
GO:0016730	oxidoreductase activity, acting on iron-sulfur proteins as donors	0.014949	0.131722	3.89011E-4	4	32	405	42094	over
GO:0006952	defense response	0.014949	0.132396	3.93913E-4	20	859	389	41267	over
GO:0045449	regulation of transcription	0.017579	0.157516	4.96539E-4	43	2583	366	39543	over
GO:0042132	fructose-bisphosphatase activity	0.0187364	0.170864	5.42906E-4	3	14	406	42112	over
GO:0009970	cellular response to sulfate starvation	0.021264	0.195847	5.46381E-4	2	2	407	42124	over
GO:0019219	regulation of nucleobase, nucleoside, nucleotide and nucleic acid metabolic process	0.0239233	0.222139	6.63176E-4	43	2621	366	39505	over
GO:0019953	sexual reproduction	0.0271408	0.253068	8.43874E-4	4	40	405	42086	over

GO:0004331	fructose-2,6-bisphosphate 2-phosphatase activity	0.0284828	0.268991	8.90892E-4	3	17	406	42109	over
GO:0051410	detoxification of nitrogen compound	0.0294699	0.297889	9.04835E-4	2	3	407	42123	over
GO:0047427	cyanoalanine nitrilase activity	0.0294699	0.297889	9.04835E-4	2	3	407	42123	over
GO:0051409	response to nitrosative stress	0.0294699	0.297889	9.04835E-4	2	3	407	42123	over
GO:0047558	3-cyanoalanine hydratase activity	0.0294699	0.297889	9.04835E-4	2	3	407	42123	over
GO:0009607	response to biotic stimulus	0.0299966	0.307526	9.87397E-4	24	1209	385	40917	over
GO:0016722	oxidoreductase activity, oxidizing metal ions	0.0342852	0.34905	0.00118646	3	19	406	42107	over
GO:0019222	regulation of metabolic process	0.0342852	0.354141	0.00122791	45	2870	364	39256	over
GO:0006350	transcription	0.0347547	0.363552	0.00130697	44	2797	365	39329	over
GO:0008281	sulfonylurea receptor activity	0.0387641	0.407478	0.00134861	2	4	407	42122	over
GO:0019499	cyanide metabolic process	0.0387641	0.407478	0.00134861	2	4	407	42122	over
GO:0043565	sequence-specific DNA binding	0.0397242	0.42251	0.00142237	11	375	398	41751	over
GO:0050661	NADP binding	0.0397242	0.427922	0.00147355	4	47	405	42079	over
GO:0031323	regulation of cellular metabolic process	0.0397242	0.432283	0.00152701	44	2820	365	39306	over
GO:0006000	fructose metabolic process	0.0401355	0.441237	0.0015373	3	21	406	42105	over
GO:0005507	copper ion binding	0.0402954	0.454286	0.00165433	8	218	401	41908	over

GO:0009685	gibberellin metabolic process	0.0402954	0.469922	0.00173455	3	22	406	42104	over
GO:0043161	proteasomal ubiquitin-dependent protein catabolic process	0.0402954	0.469922	0.00173455	3	22	406	42104	over
GO:0016101	diterpenoid metabolic process	0.0402954	0.469922	0.00173455	3	22	406	42104	over
GO:0031146	SCF-dependent proteasomal ubiquitin-dependent protein catabolic process	0.0402954	0.469922	0.00173455	3	22	406	42104	over
GO:0051791	medium-chain fatty acid metabolic process	0.044385	0.515018	0.00187605	2	5	407	42121	over
GO:0005773	vacuole	0.044385	0.515817	0.00187998	11	389	398	41737	over
GO:0006091	generation of precursor metabolites and energy	0.044385	0.519273	0.00190603	37	2281	372	39845	over
GO:0016706	oxidoreductase activity, acting on paired donors, with incorporation or reduction of molecular oxygen, 2-oxoglutarate as one donor, and incorporation of one atom each of oxygen into both donors	0.0457039	0.540162	0.0020213	6	129	403	41997	over
GO:0003677	DNA binding	0.0457039	0.540255	0.00202252	52	3536	357	38590	over

GOSSIP
Test-Set: tm1.yuc.2.up.txt
Tests for all terms in Gene Ontology whether it is enriched in a test group when compared to a reference group using Fisher's excact test with Multiple Testing.
Pub: Biological Profiling of Gene Groups utilizing Gene Ontology A Statistical Framework
Poster: GOSSIP: Biological Profiling of Gene Groups utilizing Gene Ontology
by Nils Blthgen, Karsten Brand, Hanspeter Herzel, Dieter Beule

GO Term	Name	FDR	FWER	single test p-Value	# in test group	# in reference group	# non annot test	# non annot reference group	Over/Under

GO:0009108	coenzyme biosynthetic process	2.51792E-7	7.52109E-7	0.0	50	345	1862	40278	over
GO:0015078	hydrogen ion transmembrane transporter activity	2.51792E-7	7.58652E-7	2.53814E-11	56	418	1856	40205	over
GO:0006119	oxidative phosphorylation	2.51792E-7	7.77648E-7	4.89868E-11	51	354	1861	40269	over
GO:0031966	mitochondrial membrane	2.51792E-7	8.52011E-7	3.0085E-10	66	572	1846	40051	over
GO:0019866	organelle inner membrane	2.51792E-7	9.5321E-7	5.71115E-10	63	538	1849	40085	over
GO:0009260	ribonucleotide biosynthetic process	2.51792E-7	1.02003E-6	8.83637E-10	43	294	1869	40329	over
GO:0009259	ribonucleotide metabolic process	2.51792E-7	1.06573E-6	1.01448E-9	43	295	1869	40328	over
GO:0044455	mitochondrial membrane part	2.51792E-7	1.06631E-6	1.02107E-9	40	261	1872	40362	over
GO:0015077	monovalent inorganic cation transmembrane transporter activity	2.51792E-7	1.08168E-6	1.10029E-9	56	457	1856	40166	over
GO:0009165	nucleotide biosynthetic process	2.51792E-7	1.10663E-6	1.13624E-9	50	381	1862	40242	over
GO:0051188	cofactor biosynthetic process	2.51792E-7	1.13044E-6	1.26085E-9	58	485	1854	40138	over
GO:0005743	mitochondrial inner membrane	2.51792E-7	1.23156E-6	1.65305E-9	58	488	1854	40135	over
GO:0009141	nucleoside triphosphate metabolic process	2.51792E-7	1.36733E-6	2.14491E-9	39	257	1873	40366	over
GO:0005740	mitochondrial envelope	2.51792E-7	1.44831E-6	2.37846E-9	67	615	1845	40008	over
GO:0006163	purine nucleotide metabolic process	2.51792E-7	1.44831E-6	2.37993E-9	43	305	1869	40318	over
GO:0009117	nucleotide metabolic process	2.51792E-7	1.46086E-6	2.40458E-9	65	588	1847	40035	over

GO:0006753	nucleoside phosphate metabolic process	2.51792E-7	1.46924E-6	2.43969E-9	37	236	1875	40387	over
GO:0046034	ATP metabolic process	2.51792E-7	1.46924E-6	2.43969E-9	37	236	1875	40387	over
GO:0016469	proton-transporting two-sector ATPase complex	2.51792E-7	1.46924E-6	2.43969E-9	37	236	1875	40387	over
GO:0006754	ATP biosynthetic process	2.51792E-7	1.46924E-6	2.43969E-9	37	236	1875	40387	over
GO:0009205	purine ribonucleoside triphosphate metabolic process	2.51792E-7	1.47595E-6	2.48151E-9	38	247	1874	40376	over
GO:0009206	purine ribonucleoside triphosphate biosynthetic process	2.51792E-7	1.47595E-6	2.48151E-9	38	247	1874	40376	over
GO:0009144	purine nucleoside triphosphate metabolic process	2.51792E-7	1.63665E-6	2.65582E-9	38	248	1874	40375	over
GO:0009145	purine nucleoside triphosphate biosynthetic process	2.51792E-7	1.63665E-6	2.65582E-9	38	248	1874	40375	over
GO:0009199	ribonucleoside triphosphate metabolic process	2.51792E-7	1.63665E-6	2.65582E-9	38	248	1874	40375	over
GO:0009201	ribonucleoside triphosphate biosynthetic process	2.51792E-7	1.63665E-6	2.65582E-9	38	248	1874	40375	over
GO:0009142	nucleoside triphosphate biosynthetic process	3.21304E-7	2.20742E-6	4.86698E-9	38	254	1874	40369	over
GO:0015075	ion transmembrane transporter activity	3.21304E-7	2.24912E-6	5.24364E-9	85	888	1827	39735	over
GO:0006164	purine nucleotide biosynthetic process	4.11011E-7	2.97983E-6	6.85173E-9	41	293	1871	40330	over
GO:0046933	hydrogen ion transporting ATP synthase activity, rotational mechanism	5.04866E-7	3.78649E-6	8.11919E-9	35	225	1877	40398	over
GO:0022890	inorganic cation transmembrane transporter activity	5.21979E-7	4.04533E-6	9.47366E-9	60	542	1852	40081	over
GO:0044429	mitochondrial part	5.52625E-7	4.42099E-6	1.05847E-8	71	698	1841	39925	over

GO:0006752	group transfer coenzyme metabolic process	6.1701E-7	5.09032E-6	1.25398E-8	41	300	1871	40323	over
GO:0015986	ATP synthesis coupled proton transport	7.16135E-7	6.26616E-6	1.60373E-8	35	232	1877	40391	over
GO:0015985	energy coupled proton transport, down electrochemical gradient	7.16135E-7	6.26616E-6	1.60373E-8	35	232	1877	40391	over
GO:0009152	purine ribonucleotide biosynthetic process	7.94784E-7	7.15303E-6	1.93007E-8	39	281	1873	40342	over
GO:0009150	purine ribonucleotide metabolic process	8.26657E-7	7.64655E-6	2.11087E-8	39	282	1873	40341	over
GO:0008324	cation transmembrane transporter activity	3.23374E-6	3.07201E-5	8.91193E-8	69	711	1843	39912	over
GO:0046961	hydrogen ion transporting ATPase activity, rotational mechanism	3.79345E-6	3.69854E-5	1.03539E-7	32	217	1880	40406	over
GO:0005753	mitochondrial proton-transporting ATP synthase complex	4.71792E-6	4.71781E-5	1.4083E-7	17	66	1895	40557	over
GO:0051179	localization	5.60559E-6	5.74556E-5	1.7517E-7	361	5888	1551	34735	over
GO:0006810	transport	8.27025E-6	8.68339E-5	2.2772E-7	358	5847	1554	34776	over
GO:0006563	L-serine metabolic process	9.36991E-6	1.00721E-4	2.67247E-7	20	97	1892	40526	over
GO:0051234	establishment of localization	1.05943E-5	1.16531E-4	3.14243E-7	358	5867	1554	34756	over
GO:0045259	proton-transporting ATP synthase complex	1.23934E-5	1.39416E-4	4.14445E-7	21	110	1891	40513	over
GO:0006886	intracellular protein transport	2.0516E-5	2.35906E-4	6.72936E-7	73	816	1839	39807	over
GO:0005739	mitochondrion	2.05892E-5	2.41894E-4	7.08218E-7	540	9479	1372	31144	over
GO:0051649	establishment of cellular localization	2.51859E-5	3.02185E-4	8.82216E-7	209	3146	1703	37477	over

GO:0051641	cellular localization	2.75819E-5	3.49812E-4	1.01819E-6	209	3153	1703	37470	over
GO:0016023	cytoplasmic membrane-bound vesicle	2.75819E-5	3.51607E-4	1.02568E-6	301	4858	1611	35765	over
GO:0031410	cytoplasmic vesicle	2.75819E-5	3.51607E-4	1.02568E-6	301	4858	1611	35765	over
GO:0031982	vesicle	3.08259E-5	4.0836E-4	1.06034E-6	301	4860	1611	35763	over
GO:0031988	membrane-bound vesicle	3.08259E-5	4.0836E-4	1.06034E-6	301	4860	1611	35763	over
GO:0019829	cation-transporting ATPase activity	3.10091E-5	4.22418E-4	1.14353E-6	34	270	1878	40353	over
GO:0032196	transposition	3.10091E-5	4.34033E-4	1.14599E-6	9	17	1903	40606	over
GO:0006313	transposition, DNA-mediated	3.10091E-5	4.34033E-4	1.14599E-6	9	17	1903	40606	over
GO:0004124	cysteine synthase activity	3.12965E-5	4.45875E-4	1.17766E-6	10	23	1902	40600	over
GO:0004803	transposase activity	3.24064E-5	4.75006E-4	1.27372E-6	8	12	1904	40611	over
GO:0042625	ATPase activity, coupled to transmembrane movement of ions	3.24064E-5	4.7788E-4	1.28392E-6	40	350	1872	40273	over
GO:0022891	substrate-specific transmembrane transporter activity	3.29774E-5	4.95185E-4	1.3434E-6	102	1298	1810	39325	over
GO:0009069	serine family amino acid metabolic process	3.29774E-5	5.02779E-4	1.38292E-6	26	175	1886	40448	over
GO:0016820	hydrolase activity, acting on acid anhydrides, catalyzing transmembrane movement of substances	4.48773E-5	6.95356E-4	1.78595E-6	51	509	1861	40114	over
GO:0005215	transporter activity	4.95818E-5	7.80608E-4	2.10108E-6	170	2486	1742	38137	over
GO:0015992	proton transport	5.37783E-5	8.60083E-4	2.41083E-6	40	360	1872	40263	over

GO:0006818	hydrogen transport	5.39435E-5	8.92045E-4	2.56374E-6	40	361	1872	40262	over
GO:0043492	ATPase activity, coupled to movement of substances	5.39435E-5	9.03145E-4	2.61598E-6	50	502	1862	40121	over
GO:0042626	ATPase activity, coupled to transmembrane movement of substances	5.39435E-5	9.03145E-4	2.61598E-6	50	502	1862	40121	over
GO:0031090	organelle membrane	8.272E-5	0.00140525	3.69448E-6	115	1550	1797	39073	over
GO:0042631	cellular response to water deprivation	1.16414E-4	0.00200613	4.79034E-6	7	10	1905	40613	over
GO:0006535	cysteine biosynthetic process from serine	1.16938E-4	0.00205677	4.89682E-6	10	28	1902	40595	over
GO:0022892	substrate-specific transporter activity	1.16938E-4	0.00208069	5.04748E-6	120	1647	1792	38976	over
GO:0031975	envelope	1.16938E-4	0.00210266	5.20302E-6	75	900	1837	39723	over
GO:0006811	ion transport	1.44669E-4	0.00263673	6.44646E-6	76	922	1836	39701	over
GO:0031967	organelle envelope	1.52221E-4	0.00281213	7.43701E-6	74	894	1838	39729	over
GO:0048046	apoplast	1.94603E-4	0.00367392	9.26926E-6	20	126	1892	40497	over
GO:0045045	secretory pathway	1.94603E-4	0.00369063	9.42051E-6	139	2003	1773	38620	over
GO:0016020	membrane	1.97084E-4	0.00378668	9.94637E-6	533	9559	1379	31064	over
GO:0006123	mitochondrial electron transport, cytochrome c to oxygen	2.65484E-4	0.00520821	1.38868E-5	9	25	1903	40598	over
GO:0046903	secretion	2.65484E-4	0.00529562	1.4532E-5	139	2022	1773	38601	over
GO:0032940	secretion by cell	2.65484E-4	0.00529562	1.4532E-5	139	2022	1773	38601	over

GO:0009854	oxidative photosynthetic carbon pathway	3.08716E-4	0.00623201	1.69509E-5	7	13	1905	40610	over
GO:0045277	respiratory chain complex IV	3.15696E-4	0.00652929	1.79491E-5	9	26	1903	40597	over
GO:0005751	mitochondrial respiratory chain complex IV	3.15696E-4	0.00652929	1.79491E-5	9	26	1903	40597	over
GO:0043094	metabolic compound salvage	4.62794E-4	0.00967163	2.39338E-5	26	209	1886	40414	over
GO:0047458	beta-pyrazolylalanine synthase activity	4.70519E-4	0.0100652	2.44382E-5	7	14	1905	40609	over
GO:0016778	diphosphotransferase activity	4.70519E-4	0.0100652	2.44382E-5	7	14	1905	40609	over
GO:0005746	mitochondrial respiratory chain	4.98306E-4	0.0107797	2.78576E-5	21	149	1891	40474	over
GO:0009070	serine family amino acid biosynthetic process	6.17414E-4	0.0134913	3.58272E-5	15	84	1897	40539	over
GO:0015031	protein transport	6.30374E-4	0.013928	3.67417E-5	77	991	1835	39632	over
GO:0006812	cation transport	6.8591E-4	0.0153145	3.94511E-5	64	781	1848	39842	over
GO:0022857	transmembrane transporter activity	7.73613E-4	0.0174458	4.45376E-5	119	1718	1793	38905	over
GO:0005754	mitochondrial proton-transporting ATP synthase, catalytic core	7.92871E-4	0.0182654	4.6127E-5	12	56	1900	40567	over
GO:0045267	proton-transporting ATP synthase, catalytic core	7.92871E-4	0.0182654	4.6127E-5	12	56	1900	40567	over
GO:0046907	intracellular transport	8.86963E-4	0.020628	5.53936E-5	102	1430	1810	39193	over
GO:0045184	establishment of protein localization	8.91899E-4	0.0209599	5.62278E-5	77	1005	1835	39618	over
GO:0008104	protein localization	0.00101238	0.0240044	5.79322E-5	77	1006	1835	39617	over

GO:0000275	mitochondrial proton-transporting ATP synthase complex, catalytic core F(1)	0.0010299	0.0246659	6.203E-5	12	58	1900	40565	over
GO:0000158	protein phosphatase type 2A activity	0.00109469	0.0264635	6.56309E-5	8	24	1904	40599	over
GO:0051186	cofactor metabolic process	0.00124163	0.0302631	7.50137E-5	73	948	1839	39675	over
GO:0006534	cysteine metabolic process	0.00124772	0.0310141	7.76964E-5	10	41	1902	40582	over
GO:0019344	cysteine biosynthetic process	0.00124772	0.0310141	7.76964E-5	10	41	1902	40582	over
GO:0033036	macromolecule localization	0.00125211	0.0314247	8.00919E-5	77	1017	1835	39606	over
GO:0019860	uracil metabolic process	0.00149974	0.0389641	9.06929E-5	3	0	1909	40623	over
GO:0043100	pyrimidine base salvage	0.00149974	0.0389641	9.06929E-5	3	0	1909	40623	over
GO:0006223	uracil salvage	0.00149974	0.0389641	9.06929E-5	3	0	1909	40623	over
GO:0008655	pyrimidine salvage	0.00149974	0.0389641	9.06929E-5	3	0	1909	40623	over
GO:0016192	vesicle-mediated transport	0.00161048	0.0421658	1.02427E-4	42	461	1870	40162	over
GO:0006732	coenzyme metabolic process	0.00165551	0.0437149	1.06732E-4	62	778	1850	39845	over
GO:0015405	P-P-bond-hydrolysis-driven transmembrane transporter activity	0.00167462	0.0446082	1.10503E-4	53	634	1859	39989	over
GO:0045261	proton-transporting ATP synthase complex, catalytic core F(1)	0.00177121	0.0475912	1.11016E-4	15	94	1897	40529	over
GO:0009116	nucleoside metabolic process	0.00177121	0.0479631	1.13355E-4	18	129	1894	40494	over
GO:0006206	pyrimidine base metabolic process	0.00177831	0.0489966	1.13442E-4	7	19	1905	40604	over

GO:0006122	mitochondrial electron transport, ubiquinol to cytochrome c	0.00177831	0.0489966	1.13442E-4	7	19	1905	40604	over
GO:0033178	proton-transporting two-sector ATPase complex, catalytic domain	0.00178537	0.0497423	1.18927E-4	16	106	1896	40517	over
GO:0007264	small GTPase mediated signal transduction	0.00178537	0.0500347	1.20708E-4	33	331	1879	40292	over
GO:0008168	methyltransferase activity	0.00238509	0.0678922	1.66031E-4	41	457	1871	40166	over
GO:0044444	cytoplasmic part	0.00238509	0.0678922	1.6615E-4	1193	23662	719	16961	over
GO:0006091	generation of precursor metabolites and energy	0.00238509	0.0683791	1.68543E-4	141	2177	1771	38446	over
GO:0015399	primary active transmembrane transporter activity	0.00238509	0.0684983	1.69882E-4	53	646	1859	39977	over
GO:0009853	photorespiration	0.00256174	0.0739742	1.82453E-4	19	147	1893	40476	over
GO:0016741	transferase activity, transferring one-carbon groups	0.00274719	0.0797442	1.96426E-4	41	461	1871	40162	over
GO:0016491	oxidoreductase activity	0.00282215	0.0824763	2.08273E-4	219	3647	1693	36976	over
GO:0016869	intramolecular transferase activity, transferring amino groups	0.00333987	0.0983583	2.46274E-4	4	4	1908	40619	over
GO:0042286	glutamate-1-semialdehyde 2,1-aminomutase activity	0.00333987	0.0983583	2.46274E-4	4	4	1908	40619	over
GO:0042775	organelle ATP synthesis coupled electron transport	0.00342835	0.101598	2.50753E-4	16	114	1896	40509	over
GO:0005544	calcium-dependent phospholipid binding	0.00385727	0.114415	2.90136E-4	8	31	1904	40592	over
GO:0016004	phospholipase activator activity	0.00447759	0.132524	3.47008E-4	9	41	1903	40582	over
GO:0015431	glutathione S-conjugate-exporting ATPase activity	0.00555659	0.162904	3.75447E-4	5	10	1907	40613	over

GO:0015672	monovalent inorganic cation transport	0.00587769	0.172678	4.20559E-4	41	480	1871	40143	over
GO:0042773	ATP synthesis coupled electron transport	0.00675215	0.197258	4.93939E-4	16	122	1896	40501	over
GO:0012505	endomembrane system	0.00675215	0.198397	4.98489E-4	142	2256	1770	38367	over
GO:0016676	oxidoreductase activity, acting on heme group of donors, oxygen as acceptor	0.00711005	0.213352	5.43905E-4	9	44	1903	40579	over
GO:0004129	cytochrome-c oxidase activity	0.00711005	0.213352	5.43905E-4	9	44	1903	40579	over
GO:0015002	heme-copper terminal oxidase activity	0.00711005	0.213352	5.43905E-4	9	44	1903	40579	over
GO:0016675	oxidoreductase activity, acting on heme group of donors	0.00711005	0.213352	5.43905E-4	9	44	1903	40579	over
GO:0004365	glyceraldehyde-3-phosphate dehydrogenase (phosphorylating) activity	0.00901291	0.263949	6.96479E-4	10	56	1902	40567	over
GO:0006471	protein amino acid ADP-ribosylation	0.00984439	0.286227	8.00403E-4	8	37	1904	40586	over
GO:0006888	ER to Golgi vesicle-mediated transport	0.011642	0.330804	8.98452E-4	18	156	1894	40467	over
GO:0005262	calcium channel activity	0.0122456	0.3466	9.56944E-4	5	13	1907	40610	over
GO:0007030	Golgi organization and biogenesis	0.0132482	0.371064	0.00104134	4	7	1908	40616	over
GO:0009112	nucleobase metabolic process	0.013384	0.376137	0.00106898	9	49	1903	40574	over
GO:0016899	oxidoreductase activity, acting on the CH-OH group of donors, oxygen as acceptor	0.0134229	0.383233	0.00107239	6	21	1906	40602	over
GO:0003973	(S)-2-hydroxy-acid oxidase activity	0.0134229	0.383233	0.00107239	6	21	1906	40602	over

GO:0008891	glycolate oxidase activity	0.0134229	0.383233	0.00107239	6	21	1906	40602	over
GO:0006546	glycine catabolic process	0.0144951	0.410877	0.00124468	10	61	1902	40562	over
GO:0009071	serine family amino acid catabolic process	0.0144951	0.410877	0.00124468	10	61	1902	40562	over
GO:0005737	cytoplasm	0.0150029	0.423864	0.00130013	1213	24365	699	16258	over
GO:0005216	ion channel activity	0.0160804	0.448458	0.00141801	15	123	1897	40500	over
GO:0000814	ESCRT II complex	0.0175727	0.480382	0.00150657	4	8	1908	40615	over
GO:0016979	lipoate-protein ligase activity	0.0191413	0.51222	0.00160655	5	15	1907	40608	over
GO:0000049	tRNA binding	0.0212408	0.556286	0.00163717	3	3	1909	40620	over
GO:0008934	inositol-1(or 4)-monophosphatase activity	0.0212408	0.556286	0.00163717	3	3	1909	40620	over
GO:0031403	lithium ion binding	0.0212408	0.556286	0.00163717	3	3	1909	40620	over
GO:0042623	ATPase activity, coupled	0.0221694	0.574142	0.00184806	54	740	1858	39883	over
GO:0010008	endosome membrane	0.0226046	0.585928	0.00190082	10	65	1902	40558	over
GO:0044440	endosomal part	0.0226046	0.585928	0.00190082	10	65	1902	40558	over
GO:0006118	electron transport	0.0231278	0.596634	0.00193103	97	1505	1815	39118	over
GO:0000160	two-component signal transduction system (phosphorelay)	0.0233834	0.603332	0.0019955	22	226	1890	40397	over
GO:0000097	sulfur amino acid biosynthetic process	0.0233834	0.605305	0.00201875	13	102	1899	40521	over

GO:0044426	cell wall part	0.0249766	0.64538	0.0020196	2	0	1910	40623	over
GO:0015708	silicate transport	0.0249766	0.64538	0.0020196	2	0	1910	40623	over
GO:0048226	Casparian strip	0.0249766	0.64538	0.0020196	2	0	1910	40623	over
GO:0030785	[ribulose-bisphosphate carboxylase]-lysine N-methyltransferase activity	0.0249766	0.64538	0.0020196	2	0	1910	40623	over
GO:0015115	silicate transmembrane transporter activity	0.0249766	0.64538	0.0020196	2	0	1910	40623	over
GO:0004151	dihydroorotase activity	0.0249766	0.64538	0.0020196	2	0	1910	40623	over
GO:0010211	IAA-Leu conjugate hydrolase activity	0.0249766	0.64538	0.0020196	2	0	1910	40623	over
GO:0042558	pteridine and derivative metabolic process	0.0264483	0.67078	0.00209904	4	9	1908	40614	over
GO:0042559	pteridine and derivative biosynthetic process	0.0264483	0.67078	0.00209904	4	9	1908	40614	over
GO:0000815	ESCRT III complex	0.0276323	0.688914	0.0022878	6	25	1906	40598	over
GO:0044425	membrane part	0.0297556	0.717726	0.00266327	176	3012	1736	37611	over
GO:0005576	extracellular region	0.0327378	0.753368	0.00279746	31	372	1881	40251	over
GO:0000234	phosphoethanolamine N-methyltransferase activity	0.0338325	0.766631	0.00283469	4	10	1908	40613	over
GO:0016978	lipoate-protein ligase B activity	0.0411929	0.831718	0.00372896	4	11	1908	40612	over
GO:0006897	endocytosis	0.0416054	0.8381	0.00379327	5	19	1907	40604	over
GO:0010324	membrane invagination	0.0416054	0.8381	0.00379327	5	19	1907	40604	over

GO:0022838	substrate specific channel activity	0.0422076	0.84397	0.0039695	18	181	1894	40442	over
GO:0010181	FMN binding	0.0422813	0.846112	0.00404027	10	73	1902	40550	over
GO:0005253	anion channel activity	0.0432897	0.854417	0.00425465	8	50	1904	40573	over
GO:0004385	guanylate kinase activity	0.0452451	0.871011	0.00428308	3	5	1909	40618	over
GO:0004045	aminoacyl-tRNA hydrolase activity	0.0452451	0.871011	0.00428308	3	5	1909	40618	over
GO:0005245	voltage-gated calcium channel activity	0.0452451	0.871011	0.00428308	3	5	1909	40618	over
GO:0008943	glyceraldehyde-3-phosphate dehydrogenase activity	0.0459385	0.876427	0.00440551	10	74	1902	40549	over
GO:0004462	lactoylglutathione lyase activity	0.0481251	0.889477	0.00476594	7	40	1905	40583	over
GO:0016763	transferase activity, transferring pentosyl groups	0.048133	0.89084	0.00479626	10	75	1902	40548	over
GO:0004749	ribose phosphate diphosphokinase activity	0.0493904	0.898922	0.00479664	4	12	1908	40611	over
GO:0016681	oxidoreductase activity, acting on diphenols and related substances as donors, cytochrome as acceptor	0.0493904	0.900728	0.00488203	9	63	1903	40560	over
GO:0008121	ubiquinol-cytochrome-c reductase activity	0.0493904	0.900728	0.00488203	9	63	1903	40560	over

GOSSIP
Test-Set: tm1.yuc.2.down.txt
Tests for all terms in Gene Ontology whether it is enriched in a test group when compared to a reference group using Fisher's excact test with Multiple Testing.
Pub: Biological Profiling of Gene Groups utilizing Gene Ontology A Statistical Framework
Poster: GOSSIP: Biological Profiling of Gene Groups utilizing Gene Ontology

by Nils Blthgen, Karsten Brand, Hanspeter Herzel, Dieter Beule									
**GO Term**	**Name**	**FDR**	**FWER**	**single test p-Value**	**# in test group**	**# in reference group**	**# non annot test**	**# non annot reference group**	**Over/Under**
GO:0009698	phenylpropanoid metabolic process	7.51569E-9	1.11862E-8	0.0	58	342	1391	40744	over
GO:0019748	secondary metabolic process	7.51569E-9	1.16688E-8	3.4373E-12	74	775	1375	40311	over
GO:0009699	phenylpropanoid biosynthetic process	7.51569E-9	1.57043E-8	2.05294E-11	41	277	1408	40809	over
GO:0006725	aromatic compound metabolic process	7.51569E-9	1.58075E-8	2.11513E-11	67	676	1382	40410	over
GO:0045449	regulation of transcription	7.51569E-9	1.67025E-8	2.34275E-11	180	2446	1269	38640	over
GO:0003676	nucleic acid binding	7.51569E-9	1.84792E-8	2.75725E-11	319	5800	1130	35286	over
GO:0019222	regulation of metabolic process	7.51569E-9	2.04223E-8	3.27458E-11	195	2720	1254	38366	over
GO:0009813	flavonoid biosynthetic process	7.51569E-9	2.05026E-8	3.3712E-11	34	141	1415	40945	over
GO:0003700	transcription factor activity	7.51569E-9	2.84435E-8	5.16562E-11	93	1212	1356	39874	over
GO:0050794	regulation of cellular process	7.51569E-9	3.10527E-8	6.57529E-11	219	3103	1230	37983	over
GO:0006575	amino acid derivative metabolic process	7.51569E-9	3.16559E-8	7.20185E-11	71	565	1378	40521	over
GO:0009812	flavonoid metabolic process	7.51569E-9	3.1968E-8	7.5361E-11	35	156	1414	40930	over
GO:0006351	transcription, DNA-dependent	7.51569E-9	3.1968E-8	7.54794E-11	104	1441	1345	39645	over

GO:0032774	RNA biosynthetic process	7.51569E-9	3.1968E-8	7.54794E-11	104	1441	1345	39645	over
GO:0003677	DNA binding	7.51569E-9	3.23145E-8	7.5767E-11	208	3380	1241	37706	over
GO:0030528	transcription regulator activity	7.51569E-9	3.40468E-8	7.70124E-11	122	1621	1327	39465	over
GO:0065007	biological regulation	7.51569E-9	3.71303E-8	8.56666E-11	271	4376	1178	36710	over
GO:0009753	response to jasmonic acid stimulus	7.51569E-9	3.8123E-8	8.80779E-11	36	247	1413	40839	over
GO:0050789	regulation of biological process	7.51569E-9	3.83965E-8	9.00263E-11	242	3583	1207	37503	over
GO:0031323	regulation of cellular metabolic process	7.51569E-9	3.84566E-8	9.08529E-11	191	2673	1258	38413	over
GO:0019219	regulation of nucleobase, nucleoside, nucleotide and nucleic acid metabolic process	7.51569E-9	4.11354E-8	9.65475E-11	183	2481	1266	38605	over
GO:0016210	naringenin-chalcone synthase activity	7.51569E-9	4.13363E-8	9.8215E-11	16	14	1433	41072	over
GO:0006355	regulation of transcription, DNA-dependent	7.66635E-9	4.40815E-8	1.11055E-10	100	1385	1349	39701	over
GO:0006350	transcription	7.91838E-9	4.76419E-8	1.32787E-10	188	2653	1261	38433	over
GO:0042398	amino acid derivative biosynthetic process	7.91838E-9	4.94898E-8	1.43521E-10	51	451	1398	40635	over
GO:0005634	nucleus	2.60385E-8	1.75536E-7	5.15696E-10	208	3793	1241	37293	over
GO:0019438	aromatic compound biosynthetic process	2.60385E-8	1.7576E-7	5.18175E-10	42	373	1407	40713	over
GO:0032501	multicellular organismal process	2.86393E-8	2.00475E-7	6.2646E-10	141	2292	1308	38794	over
GO:0006559	L-phenylalanine catabolic process	2.88856E-8	2.09421E-7	7.06208E-10	12	22	1437	41064	over

GO:0048856	anatomical structure development	4.79945E-8	3.59959E-7	1.26174E-9	132	2126	1317	38960	over
GO:0043565	sequence-specific DNA binding	8.88782E-8	6.88806E-7	2.02206E-9	39	347	1410	40739	over
GO:0007275	multicellular organismal development	9.76334E-8	7.81067E-7	2.19821E-9	131	2127	1318	38959	over
GO:0009074	aromatic amino acid family catabolic process	1.1326E-7	9.34396E-7	2.82548E-9	12	26	1437	41060	over
GO:0032502	developmental process	1.81518E-7	1.5429E-6	5.217E-9	162	2850	1287	38236	over
GO:0048731	system development	2.9771E-7	2.67938E-6	9.21787E-9	76	1034	1373	40052	over
GO:0048513	organ development	2.9771E-7	2.67938E-6	9.21787E-9	76	1034	1373	40052	over
GO:0019439	aromatic compound catabolic process	6.18792E-7	5.8767E-6	1.79795E-8	12	32	1437	41054	over
GO:0047461	(+)-delta-cadinene synthase activity	6.18792E-7	5.87851E-6	1.79875E-8	8	8	1441	41078	over
GO:0045548	phenylalanine ammonia-lyase activity	7.36225E-7	7.17817E-6	2.27195E-8	10	19	1439	41067	over
GO:0006558	L-phenylalanine metabolic process	7.48663E-7	7.4866E-6	2.38432E-8	13	41	1436	41045	over
GO:0016070	RNA metabolic process	9.90345E-7	1.0151E-5	3.34224E-8	128	2168	1321	38918	over
GO:0016880	acid-ammonia (or amide) ligase activity	1.94634E-6	2.0923E-5	5.98071E-8	13	45	1436	41041	over
GO:0016211	ammonia ligase activity	1.94634E-6	2.0923E-5	5.98071E-8	13	45	1436	41041	over
GO:0006305	DNA alkylation	5.98058E-6	6.72793E-5	2.12852E-7	12	42	1437	41044	over
GO:0006306	DNA methylation	5.98058E-6	6.72793E-5	2.12852E-7	12	42	1437	41044	over

GO:0009310	amine catabolic process	6.88976E-6	8.09513E-5	2.57321E-7	24	186	1425	40900	over
GO:0009063	amino acid catabolic process	6.88976E-6	8.09513E-5	2.57321E-7	24	186	1425	40900	over
GO:0009791	post-embryonic development	8.67366E-6	1.04079E-4	2.87088E-7	61	832	1388	40254	over
GO:0044270	nitrogen compound catabolic process	1.34954E-5	1.65306E-4	4.7157E-7	24	193	1425	40893	over
GO:0016841	ammonia-lyase activity	1.44779E-5	1.80957E-4	5.26469E-7	10	29	1439	41057	over
GO:0006139	nucleobase, nucleoside, nucleotide and nucleic acid metabolic process	2.68862E-5	3.4274E-4	1.01592E-6	253	5329	1196	35757	over
GO:0006346	methylation-dependent chromatin silencing	2.8579E-5	3.71457E-4	1.11033E-6	10	32	1439	41054	over
GO:0006519	amino acid and derivative metabolic process	3.41501E-5	4.52386E-4	1.24617E-6	95	1588	1354	39498	over
GO:0009725	response to hormone stimulus	1.19852E-4	0.00161669	4.81851E-6	89	1511	1360	39575	over
GO:0009733	response to auxin stimulus	1.37693E-4	0.00189149	5.31149E-6	49	675	1400	40411	over
GO:0043414	biopolymer methylation	1.64987E-4	0.0023483	6.28636E-6	12	61	1437	41025	over
GO:0032259	methylation	1.64987E-4	0.0023483	6.28636E-6	12	61	1437	41025	over
GO:0040029	regulation of gene expression, epigenetic	2.05828E-4	0.00298007	7.85493E-6	22	199	1427	40887	over
GO:0009719	response to endogenous stimulus	3.09575E-4	0.00455582	1.24644E-5	117	2190	1332	38896	over
GO:0009653	anatomical structure morphogenesis	3.51103E-4	0.00528274	1.47305E-5	63	992	1386	40094	over
GO:0006342	chromatin silencing	3.51103E-4	0.00534002	1.47635E-5	10	45	1439	41041	over

GO:0030155	regulation of cell adhesion	4.82282E-4	0.00744751	1.90423E-5	4	2	1445	41084	over
GO:0032200	telomere organization and biogenesis	4.94529E-4	0.00801638	2.01823E-5	6	12	1443	41074	over
GO:0000723	telomere maintenance	4.94529E-4	0.00801638	2.01823E-5	6	12	1443	41074	over
GO:0045814	negative regulation of gene expression, epigenetic	4.94529E-4	0.00809839	2.04925E-5	10	47	1439	41039	over
GO:0006338	chromatin remodeling	4.94529E-4	0.00812654	2.05336E-5	11	58	1438	41028	over
GO:0050896	response to stimulus	7.5316E-4	0.0125362	3.3927E-5	250	5528	1199	35558	over
GO:0016711	flavonoid 3'-monooxygenase activity	9.42506E-4	0.015895	3.94545E-5	3	0	1446	41086	over
GO:0009975	cyclase activity	9.5734E-4	0.0164685	3.97421E-5	6	14	1443	41072	over
GO:0004497	monooxygenase activity	9.5734E-4	0.0166139	4.03177E-5	28	327	1421	40759	over
GO:0031507	heterochromatin formation	0.00108853	0.019307	4.37364E-5	10	52	1439	41034	over
GO:0046686	response to cadmium ion	0.00108853	0.0194029	4.39388E-5	12	76	1437	41010	over
GO:0010374	stomatal complex development	0.00124403	0.0236631	5.40349E-5	6	15	1443	41071	over
GO:0010103	stomatal complex morphogenesis	0.00124403	0.0236631	5.40349E-5	6	15	1443	41071	over
GO:0006529	asparagine biosynthetic process	0.00124403	0.0236631	5.40349E-5	6	15	1443	41071	over
GO:0006528	asparagine metabolic process	0.00124403	0.0236631	5.40349E-5	6	15	1443	41071	over
GO:0004066	asparagine synthase (glutamine-hydrolyzing) activity	0.00124403	0.0236631	5.40349E-5	6	15	1443	41071	over

GO:0016458	gene silencing	0.0012598	0.0242668	5.71819E-5	16	134	1433	40952	over
GO:0016840	carbon-nitrogen lyase activity	0.00126076	0.0245927	5.79367E-5	10	54	1439	41032	over
GO:0009888	tissue development	0.00132113	0.0260768	6.51233E-5	32	411	1417	40675	over
GO:0046423	allene-oxide cyclase activity	0.00142647	0.028473	7.05287E-5	5	9	1444	41077	over
GO:0010193	response to ozone	0.00149028	0.0300889	7.47844E-5	8	34	1441	41052	over
GO:0000080	G1 phase of mitotic cell cycle	0.00161803	0.0341898	8.41126E-5	4	4	1445	41082	over
GO:0051213	dioxygenase activity	0.00161803	0.0341898	8.41126E-5	4	4	1445	41082	over
GO:0051318	G1 phase	0.00161803	0.0341898	8.41126E-5	4	4	1445	41082	over
GO:0050589	leucocyanidin oxygenase activity	0.00161803	0.0341898	8.41126E-5	4	4	1445	41082	over
GO:0016838	carbon-oxygen lyase activity, acting on phosphates	0.001648	0.0352095	8.91483E-5	8	35	1441	41051	over
GO:0051707	response to other organism	0.00204585	0.0440111	1.20021E-4	54	876	1395	40210	over
GO:0035061	interchromatin granule	0.00208695	0.0453734	1.22782E-4	6	18	1443	41068	over
GO:0010252	auxin homeostasis	0.00214078	0.0470263	1.259E-4	7	27	1442	41059	over
GO:0022402	cell cycle process	0.00216249	0.0480065	1.3025E-4	25	297	1424	40789	over
GO:0042162	telomeric DNA binding	0.00239004	0.053488	1.45363E-4	5	11	1444	41075	over
GO:0016710	trans-cinnamate 4-monooxygenase activity	0.00256242	0.0578371	1.47306E-4	4	5	1445	41081	over

GO:0004170	dUTP diphosphatase activity	0.00286158	0.0670411	1.53794E-4	3	1	1446	41085	over
GO:0046080	dUTP metabolic process	0.00286158	0.0670411	1.53794E-4	3	1	1446	41085	over
GO:0047429	nucleoside-triphosphate diphosphatase activity	0.00286158	0.0670411	1.53794E-4	3	1	1446	41085	over
GO:0009394	2'-deoxyribonucleotide metabolic process	0.00286158	0.0670411	1.53794E-4	3	1	1446	41085	over
GO:0000278	mitotic cell cycle	0.00309837	0.0731014	1.80514E-4	11	76	1438	41010	over
GO:0009585	red, far-red light phototransduction	0.00312665	0.0747011	1.86351E-4	9	51	1440	41035	over
GO:0031981	nuclear lumen	0.00312665	0.0751905	1.88465E-4	31	418	1418	40668	over
GO:0016879	ligase activity, forming carbon-nitrogen bonds	0.00337471	0.0826626	2.09316E-4	47	747	1402	40339	over
GO:0015250	water channel activity	0.00337471	0.0832315	2.12057E-4	9	52	1440	41034	over
GO:0005372	water transporter activity	0.00337471	0.0832315	2.12057E-4	9	52	1440	41034	over
GO:0022403	cell cycle phase	0.00385974	0.0954837	2.4668E-4	15	138	1434	40948	over
GO:0042221	response to chemical stimulus	0.00417141	0.103719	2.67619E-4	143	3004	1306	38082	over
GO:0005402	cation:sugar symporter activity	0.0042287	0.106957	2.6938E-4	5	13	1444	41073	over
GO:0015295	solute:hydrogen symporter activity	0.0042287	0.106957	2.6938E-4	5	13	1444	41073	over
GO:0016569	covalent chromatin modification	0.00423567	0.109776	2.81479E-4	10	67	1439	41019	over
GO:0007600	sensory perception	0.00423567	0.116528	3.0743E-4	9	55	1440	41031	over

GO:0050962	detection of light stimulus during sensory perception	0.00423567	0.116528	3.0743E-4	9	55	1440	41031	over
GO:0050908	detection of light stimulus during visual perception	0.00423567	0.116528	3.0743E-4	9	55	1440	41031	over
GO:0050906	detection of stimulus during sensory perception	0.00423567	0.116528	3.0743E-4	9	55	1440	41031	over
GO:0050953	sensory perception of light stimulus	0.00423567	0.116528	3.0743E-4	9	55	1440	41031	over
GO:0007602	phototransduction	0.00423567	0.116528	3.0743E-4	9	55	1440	41031	over
GO:0009583	detection of light stimulus	0.00423567	0.116528	3.0743E-4	9	55	1440	41031	over
GO:0007601	visual perception	0.00423567	0.116528	3.0743E-4	9	55	1440	41031	over
GO:0009584	detection of visible light	0.00423567	0.116528	3.0743E-4	9	55	1440	41031	over
GO:0005506	iron ion binding	0.00450783	0.124521	3.4391E-4	47	765	1402	40321	over
GO:0000781	chromosome, telomeric region	0.00471652	0.13092	3.55356E-4	5	14	1444	41072	over
GO:0016630	protochlorophyllide reductase activity	0.00499098	0.140136	3.65246E-4	4	7	1445	41079	over
GO:0003968	RNA-directed RNA polymerase activity	0.00499098	0.140136	3.65246E-4	4	7	1445	41079	over
GO:0048479	style development	0.00555021	0.156904	3.74698E-4	3	2	1446	41084	over
GO:0048480	stigma development	0.00555021	0.156904	3.74698E-4	3	2	1446	41084	over
GO:0031418	L-ascorbic acid binding	0.00568355	0.161546	4.03335E-4	8	45	1441	41041	over
GO:0045892	negative regulation of transcription, DNA-dependent	0.00573221	0.164009	4.2642E-4	10	71	1439	41015	over

GO:0051704	multi-organism process	0.00573316	0.165427	4.30317E-4	58	1015	1391	40071	over
GO:0045595	regulation of cell differentiation	0.00573316	0.166427	4.3576E-4	9	58	1440	41028	over
GO:0004029	aldehyde dehydrogenase (NAD) activity	0.00590319	0.173359	4.54821E-4	6	24	1443	41062	over
GO:0016884	carbon-nitrogen ligase activity, with glutamine as amido-N-donor	0.00590319	0.173359	4.54821E-4	6	24	1443	41062	over
GO:0009581	detection of external stimulus	0.00618695	0.182156	4.71013E-4	10	72	1439	41014	over
GO:0009072	aromatic amino acid family metabolic process	0.00645502	0.190558	5.16041E-4	13	117	1436	40969	over
GO:0042178	xenobiotic catabolic process	0.00698668	0.205919	5.33099E-4	4	8	1445	41078	over
GO:0050877	neurological process	0.00698989	0.20739	5.43588E-4	9	60	1440	41026	over
GO:0009723	response to ethylene stimulus	0.00712424	0.212327	5.65896E-4	17	184	1432	40902	over
GO:0006527	arginine catabolic process	0.00743115	0.221832	5.876E-4	5	16	1444	41070	over
GO:0045552	dihydrokaempferol 4-reductase activity	0.00923939	0.271281	7.30354E-4	3	3	1446	41083	over
GO:0050505	hydroquinone glucosyltransferase activity	0.00923939	0.271281	7.30354E-4	3	3	1446	41083	over
GO:0008395	steroid hydroxylase activity	0.00940782	0.277176	7.392E-4	5	17	1444	41069	over
GO:0051219	phosphoprotein binding	0.00982339	0.289214	7.49297E-4	4	9	1445	41077	over
GO:0016481	negative regulation of transcription	0.010248	0.301417	8.02481E-4	15	156	1434	40930	over
GO:0009582	detection of abiotic stimulus	0.0103269	0.305142	8.24974E-4	9	64	1440	41022	over

GO:0015293	symporter activity	0.0106227	0.314173	8.86306E-4	19	228	1430	40858	over
GO:0042742	defense response to bacterium	0.0111181	0.328001	9.34151E-4	18	211	1431	40875	over
GO:0009410	response to xenobiotic stimulus	0.0119531	0.351655	0.0010208	4	10	1445	41076	over
GO:0006805	xenobiotic metabolic process	0.0119531	0.351655	0.0010208	4	10	1445	41076	over
GO:0009314	response to radiation	0.0123791	0.363566	0.00109771	51	900	1398	40186	over
GO:0015291	secondary active transmembrane transporter activity	0.0127495	0.374111	0.00113381	29	428	1420	40658	over
GO:0006541	glutamine metabolic process	0.01493	0.42559	0.00121475	9	68	1440	41018	over
GO:0048518	positive regulation of biological process	0.01493	0.426615	0.00123164	19	235	1430	40851	over
GO:0030643	cellular phosphate ion homeostasis	0.0149637	0.449572	0.0012457	3	4	1446	41082	over
GO:0009102	biotin biosynthetic process	0.0149637	0.449572	0.0012457	3	4	1446	41082	over
GO:0055081	anion homeostasis	0.0149637	0.449572	0.0012457	3	4	1446	41082	over
GO:0030002	cellular anion homeostasis	0.0149637	0.449572	0.0012457	3	4	1446	41082	over
GO:0055061	di-, tri-valent inorganic anion homeostasis	0.0149637	0.449572	0.0012457	3	4	1446	41082	over
GO:0008506	sucrose:hydrogen symporter activity	0.0149637	0.449572	0.0012457	3	4	1446	41082	over
GO:0055062	phosphate ion homeostasis	0.0149637	0.449572	0.0012457	3	4	1446	41082	over
GO:0030319	cellular di-, tri-valent inorganic anion homeostasis	0.0149637	0.449572	0.0012457	3	4	1446	41082	over

GO:0006768	biotin metabolic process	0.0149637	0.449572	0.0012457	3	4	1446	41082	over
GO:0042325	regulation of phosphorylation	0.0149637	0.452348	0.00125372	6	30	1443	41056	over
GO:0016986	transcription initiation factor activity	0.0149637	0.452348	0.00125372	6	30	1443	41056	over
GO:0007389	pattern specification process	0.0149637	0.453027	0.001259	17	199	1432	40887	over
GO:0045934	negative regulation of nucleobase, nucleoside, nucleotide and nucleic acid metabolic process	0.0149637	0.454522	0.00127652	15	164	1434	40922	over
GO:0004033	aldo-keto reductase activity	0.015021	0.457826	0.00128982	7	42	1442	41044	over
GO:0009416	response to light stimulus	0.017069	0.503441	0.00147914	50	891	1399	40195	over
GO:0009908	flower development	0.017069	0.505487	0.00152062	29	437	1420	40649	over
GO:0031324	negative regulation of cellular metabolic process	0.017188	0.510018	0.00159044	15	168	1434	40918	over
GO:0006952	defense response	0.0192869	0.554652	0.00178148	47	832	1402	40254	over
GO:0046983	protein dimerization activity	0.0192869	0.557505	0.00181324	23	321	1426	40765	over
GO:0015288	porin activity	0.0192869	0.559484	0.00182733	13	136	1436	40950	over
GO:0022829	wide pore channel activity	0.0192869	0.559484	0.00182733	13	136	1436	40950	over
GO:0048437	floral organ development	0.0193129	0.564201	0.00187763	16	189	1433	40897	over
GO:0048569	post-embryonic organ development	0.0193129	0.564201	0.00187763	16	189	1433	40897	over
GO:0009639	response to red or far red light	0.0194023	0.567975	0.00190227	18	226	1431	40860	over

GO:0018117	protein amino acid adenylylation	0.0207373	0.598514	0.00194265	3	5	1446	41081	over
GO:0018175	protein amino acid nucleotidylation	0.0207373	0.598514	0.00194265	3	5	1446	41081	over
GO:0010279	indole-3-acetic acid amido synthetase	0.0207373	0.598514	0.00194265	3	5	1446	41081	over
GO:0009913	epidermal cell differentiation	0.0207526	0.611021	0.00198933	18	227	1431	40859	over
GO:0007398	ectoderm development	0.0207526	0.611021	0.00198933	18	227	1431	40859	over
GO:0048730	epidermis morphogenesis	0.0207526	0.611021	0.00198933	18	227	1431	40859	over
GO:0008544	epidermis development	0.0207526	0.611021	0.00198933	18	227	1431	40859	over
GO:0048729	tissue morphogenesis	0.0207526	0.611021	0.00198933	18	227	1431	40859	over
GO:0050793	regulation of developmental process	0.0207526	0.612561	0.00202083	25	364	1424	40722	over
GO:0009064	glutamine family amino acid metabolic process	0.0207526	0.613097	0.00203045	14	155	1435	40931	over
GO:0005351	sugar:hydrogen ion symporter activity	0.0209859	0.619211	0.0020712	15	173	1434	40913	over
GO:0019220	regulation of phosphate metabolic process	0.0213774	0.629988	0.00220098	6	34	1443	41052	over
GO:0051174	regulation of phosphorus metabolic process	0.0213774	0.629988	0.00220098	6	34	1443	41052	over
GO:0006493	protein amino acid O-linked glycosylation	0.0218109	0.639346	0.00223695	4	13	1445	41073	over
GO:0009628	response to abiotic stimulus	0.0226223	0.654734	0.00235603	104	2204	1345	38882	over
GO:0009892	negative regulation of metabolic process	0.0228326	0.660079	0.00241365	15	176	1434	40910	over

GO:0048438	floral whorl development	0.0231954	0.669466	0.00252056	14	159	1435	40927	over
GO:0046914	transition metal ion binding	0.0231954	0.669712	0.00253323	124	2708	1325	38378	over
GO:0051329	interphase of mitotic cell cycle	0.0249724	0.702225	0.00279911	4	14	1445	41072	over
GO:0008792	arginine decarboxylase activity	0.0249724	0.702225	0.00279911	4	14	1445	41072	over
GO:0051325	interphase	0.0249724	0.702225	0.00279911	4	14	1445	41072	over
GO:0046125	pyrimidine deoxyribonucleoside metabolic process	0.0264353	0.728076	0.00284032	3	6	1446	41080	over
GO:0009120	deoxyribonucleoside metabolic process	0.0264353	0.728076	0.00284032	3	6	1446	41080	over
GO:0009211	pyrimidine deoxyribonucleoside triphosphate metabolic process	0.0264353	0.728076	0.00284032	3	6	1446	41080	over
GO:0016563	transcription activator activity	0.0267624	0.736633	0.00302621	16	199	1433	40887	over
GO:0048364	root development	0.0267624	0.737744	0.00306304	24	356	1425	40730	over
GO:0022622	root system development	0.0267624	0.737744	0.00306304	24	356	1425	40730	over
GO:0000323	lytic vacuole	0.02713	0.744263	0.00313817	7	50	1442	41036	over
GO:0009065	glutamine family amino acid catabolic process	0.027839	0.756631	0.0031926	5	25	1444	41061	over
GO:0006855	multidrug transport	0.027839	0.756631	0.0031926	5	25	1444	41061	over
GO:0010062	negative regulation of trichoblast fate	0.0316377	0.804029	0.00340026	2	1	1447	41085	over
GO:0045430	chalcone isomerase activity	0.0316377	0.804029	0.00340026	2	1	1447	41085	over

GO:0051782	negative regulation of cell division	0.0316377	0.804029	0.00340026	2	1	1447	41085	over
GO:0048440	carpel development	0.0335289	0.823711	0.00366395	8	66	1441	41020	over
GO:0016607	nuclear speck	0.0342841	0.831919	0.00381892	7	52	1442	41034	over
GO:0048869	cellular developmental process	0.034354	0.833963	0.0038783	38	665	1411	40421	over
GO:0009200	deoxyribonucleoside triphosphate metabolic process	0.0361824	0.850458	0.00395523	3	7	1446	41079	over
GO:0009887	organ morphogenesis	0.0362887	0.852635	0.00400431	20	283	1429	40803	over
GO:0004672	protein kinase activity	0.0363619	0.854532	0.00404705	99	2122	1350	38964	over
GO:0010017	red or far red light signaling pathway	0.0373976	0.867016	0.00419861	7	53	1442	41033	over
GO:0010087	vascular tissue development (sensu Tracheophyta)	0.0373976	0.867016	0.00419861	7	53	1442	41033	over
GO:0007049	cell cycle	0.0373976	0.868174	0.00426498	26	408	1423	40678	over
GO:0016128	phytosteroid metabolic process	0.0373976	0.870768	0.00426534	5	27	1444	41059	over
GO:0015893	drug transport	0.0373976	0.870768	0.00426534	5	27	1444	41059	over
GO:0016131	brassinosteroid metabolic process	0.0373976	0.870768	0.00426534	5	27	1444	41059	over
GO:0009739	response to gibberellin stimulus	0.0373976	0.871036	0.00428514	15	188	1434	40898	over
GO:0016568	chromatin modification	0.0377744	0.874859	0.00445483	11	117	1438	40969	over
GO:0009850	auxin metabolic process	0.0392688	0.885868	0.00487428	12	136	1437	40950	over

GO:0005654	nucleoplasm	0.0395986	0.889501	0.00490492	16	210	1433	40876	over
GO:0030154	cell differentiation	0.0395986	0.89013	0.00495645	36	631	1413	40455	over
GO:0042445	hormone metabolic process	0.0419294	0.904535	0.00525207	17	231	1432	40855	over
GO:0044451	nucleoplasm part	0.0440647	0.91623	0.00535277	15	193	1434	40893	over
GO:0015238	drug transporter activity	0.0454594	0.924228	0.00559044	6	42	1443	41044	over
GO:0051606	detection of stimulus	0.0454594	0.924299	0.00560914	11	121	1438	40965	over
GO:0051119	sugar transmembrane transporter activity	0.0457548	0.927482	0.00583587	15	195	1434	40891	over
GO:0009861	jasmonic acid and ethylene-dependent systemic resistance	0.0457548	0.928082	0.00589824	9	88	1440	40998	over
GO:0009741	response to brassinosteroid stimulus	0.0457548	0.928891	0.00593159	11	122	1438	40964	over
GO:0048522	positive regulation of cellular process	0.0457548	0.928891	0.00593159	11	122	1438	40964	over
GO:0008515	sucrose transmembrane transporter activity	0.0465165	0.933519	0.0060054	4	18	1445	41068	over
GO:0015154	disaccharide transmembrane transporter activity	0.0465165	0.933519	0.0060054	4	18	1445	41068	over
GO:0045935	positive regulation of nucleobase, nucleoside, nucleotide and nucleic acid metabolic process	0.0473554	0.938176	0.00619052	6	43	1443	41043	over
GO:0045941	positive regulation of transcription	0.0473554	0.938176	0.00619052	6	43	1443	41043	over
GO:0005488	binding	0.0486188	0.943298	0.00642472	699	18444	750	22642	over
GO:0009617	response to bacterium	0.0487622	0.945022	0.00663387	21	318	1428	40768	over

GO:0009734	auxin mediated signaling pathway	0.0487622	0.945132	0.00664104	14	179	1435	40907	over

GOSSIP
Test-Set: tm1.yuc.7.up.txt
Tests for all terms in Gene Ontology whether it is enriched in a test group when compared to a reference group using Fisher's exacat test with Multiple Testing.
Pub: Biological Profiling of Gene Groups utilizing Gene Ontology A Statistical Framework
Poster: GOSSIP: Biological Profiling of Gene Groups utilizing Gene Ontology
by Nils Blthgen, Karsten Brand, Hanspeter Herzel, Dieter Beule

GO Term	Name	FDR	FWER	single test p-Value	# in test group	# in reference group	# non annot test	# non annot reference group	Over/Under
GO:0015267	channel activity	4.42831E-6	2.38596E-6	7.82592E-9	22	248	760	41505	over
GO:0022803	passive transmembrane transporter activity	4.42831E-6	2.38596E-6	7.82592E-9	22	248	760	41505	over
GO:0042631	cellular response to water deprivation	4.42831E-6	3.32123E-6	1.15065E-8	7	10	775	41743	over
GO:0015288	porin activity	3.52107E-5	4.40125E-5	1.21589E-7	15	134	767	41619	over
GO:0022829	wide pore channel activity	3.52107E-5	4.40125E-5	1.21589E-7	15	134	767	41619	over
GO:0042286	glutamate-1-semialdehyde 2,1-aminomutase activity	0.0016142	0.00282087	7.48299E-6	4	4	778	41749	over
GO:0016869	intramolecular transferase activity, transferring amino groups	0.0016142	0.00282087	7.48299E-6	4	4	778	41749	over
GO:0042277	peptide binding	0.00242793	0.0048441	1.38635E-5	8	52	774	41701	over
GO:0019867	outer membrane	0.00265201	0.00594925	1.77581E-5	16	233	766	41520	over

GO:0022838	substrate specific channel activity	0.00276821	0.00689664	2.16325E-5	14	185	768	41568	over
GO:0005576	extracellular region	0.00848982	0.0257567	7.03805E-5	20	383	762	41370	over
GO:0043269	regulation of ion transport	0.00848982	0.0272149	7.10215E-5	4	9	778	41744	over
GO:0051049	regulation of transport	0.00848982	0.0272149	7.10215E-5	4	9	778	41744	over
GO:0000234	phosphoethanolamine N-methyltransferase activity	0.010028	0.0344895	9.7983E-5	4	10	778	41743	over
GO:0015431	glutathione S-conjugate-exporting ATPase activity	0.0122444	0.0507083	1.3167E-4	4	11	778	41742	over
GO:0009288	flagellin-based flagellum	0.0122444	0.0507083	1.3167E-4	4	11	778	41742	over
GO:0001539	ciliary or flagellar motility	0.0122444	0.0507083	1.3167E-4	4	11	778	41742	over
GO:0005509	calcium ion binding	0.0129339	0.0565417	1.57641E-4	29	717	753	41036	over
GO:0017101	aminoacyl-tRNA synthetase multienzyme complex	0.0160862	0.0776694	2.05029E-4	3	4	779	41749	over
GO:0051674	localization of cell	0.0160862	0.0832288	2.22954E-4	4	13	778	41740	over
GO:0006928	cell motility	0.0160862	0.0832288	2.22954E-4	4	13	778	41740	over
GO:0005216	ion channel activity	0.0160862	0.0886452	2.49379E-4	10	128	772	41625	over
GO:0033554	cellular response to stress	0.0160862	0.0898668	2.53323E-4	7	61	775	41692	over
GO:0005262	calcium channel activity	0.0160862	0.0992815	2.82494E-4	4	14	778	41739	over
GO:0019861	flagellum	0.0160862	0.0992815	2.82494E-4	4	14	778	41739	over

GO:0042995	cell projection	0.0160862	0.0992815	2.82494E-4	4	14	778	41739	over
GO:0051716	cellular response to stimulus	0.0178554	0.117495	3.31411E-4	7	64	775	41689	over
GO:0031668	cellular response to extracellular stimulus	0.0178554	0.117495	3.31411E-4	7	64	775	41689	over
GO:0004151	dihydroorotase activity	0.0199642	0.134756	3.37579E-4	2	0	780	41753	over
GO:0009854	oxidative photosynthetic carbon pathway	0.0234014	0.160976	4.34401E-4	4	16	778	41737	over
GO:0015671	oxygen transport	0.024287	0.178592	4.787E-4	3	6	779	41747	over
GO:0015669	gas transport	0.024287	0.178592	4.787E-4	3	6	779	41747	over
GO:0046873	metal ion transmembrane transporter activity	0.024287	0.181576	4.93641E-4	12	195	770	41558	over
GO:0005253	anion channel activity	0.0321746	0.239286	6.80404E-4	6	52	776	41701	over
GO:0009414	response to water deprivation	0.0411016	0.302089	9.24226E-4	18	403	764	41350	over
GO:0003785	actin monomer binding	0.0469977	0.344929	0.00100035	2	1	780	41752	over

GOSSIP
Test-Set: tm1.yuc.7.down.txt
Tests for all terms in Gene Ontology whether it is enriched in a test group when compared to a reference group using Fisher's excact test with Multiple Testing.
Pub: Biological Profiling of Gene Groups utilizing Gene Ontology A Statistical Framework
Poster: GOSSIP: Biological Profiling of Gene Groups utilizing Gene Ontology
by Nils Blthgen, Karsten Brand, Hanspeter Herzel, Dieter Beule

GO Term	Name	FDR	FWER	single test	# in test	# in reference	# non annot	# non annot	Over/Under

				p-Value	group	group	test	reference group	
GO:0016210	naringenin-chalcone synthase activity	1.14806E-8	1.71789E-8	0.0	14	16	474	42031	over
GO:0009813	flavonoid biosynthetic process	1.14806E-8	1.71789E-8	0.0	31	144	457	41903	over
GO:0006519	amino acid and derivative metabolic process	1.14806E-8	1.71789E-8	0.0	75	1608	413	40439	over
GO:0006575	amino acid derivative metabolic process	1.14806E-8	1.71789E-8	0.0	49	587	439	41460	over
GO:0019438	aromatic compound biosynthetic process	1.14806E-8	1.80805E-8	4.94741E-12	37	378	451	41669	over
GO:0009812	flavonoid metabolic process	1.14806E-8	2.53391E-8	3.06513E-11	31	160	457	41887	over
GO:0006725	aromatic compound metabolic process	1.14806E-8	2.68171E-8	4.26406E-11	47	696	441	41351	over
GO:0042398	amino acid derivative biosynthetic process	1.14806E-8	2.69704E-8	4.46489E-11	38	464	450	41583	over
GO:0009698	phenylpropanoid metabolic process	1.14806E-8	2.73124E-8	4.69959E-11	43	357	445	41690	over
GO:0003824	catalytic activity	1.14806E-8	3.12609E-8	6.72474E-11	298	18211	190	23836	over
GO:0019748	secondary metabolic process	1.14806E-8	3.15717E-8	7.16927E-11	54	795	434	41252	over
GO:0009699	phenylpropanoid biosynthetic process	1.20341E-8	3.61023E-8	8.32444E-	37	281	451	41766	over

				11					
GO:0016491	oxidoreductase activity	3.99501E-7	1.29838E-6	4.47789E-9	85	3781	403	38266	over
GO:0016211	ammonia ligase activity	1.70411E-6	6.39041E-6	2.07303E-8	9	49	479	41998	over
GO:0016880	acid-ammonia (or amide) ligase activity	1.70411E-6	6.39041E-6	2.07303E-8	9	49	479	41998	over
GO:0019752	carboxylic acid metabolic process	1.86803E-6	8.42072E-6	2.60855E-8	55	2088	433	39959	over
GO:0006082	organic acid metabolic process	1.86803E-6	8.44769E-6	2.64265E-8	55	2089	433	39958	over
GO:0005506	iron ion binding	1.86803E-6	8.68767E-6	2.8489E-8	30	782	458	41265	over
GO:0006807	nitrogen compound metabolic process	1.86803E-6	8.87312E-6	2.93435E-8	43	1430	445	40617	over
GO:0004497	monooxygenase activity	2.24385E-6	1.12192E-5	4.0073E-8	19	336	469	41711	over
GO:0009064	glutamine family amino acid metabolic process	5.52169E-6	2.89884E-5	9.22503E-8	13	156	475	41891	over
GO:0008395	steroid hydroxylase activity	7.43677E-6	4.09014E-5	1.41253E-7	6	16	482	42031	over
GO:0006541	glutamine metabolic process	1.50849E-5	8.67347E-5	2.58845E-7	9	68	479	41979	over
GO:0008415	acyltransferase activity	2.11087E-5	1.26644E-4	4.25296E-7	23	563	465	41484	over
GO:0016747	transferase activity, transferring groups other than amino-acyl groups	2.70412E-5	1.68993E-4	5.69067E-7	23	573	465	41474	over
GO:0051213	dioxygenase activity	5.39226E-5	3.63911E-4	1.15516E-6	4	4	484	42043	over
GO:0050589	leucocyanidin oxygenase activity	5.39226E-5	3.63911E-4	1.15516E-6	4	4	484	42043	over

GO:0016711	flavonoid 3'-monooxygenase activity	6.91952E-5	5.22296E-4	1.50096E-6	3	0	485	42047	over
GO:0009308	amine metabolic process	6.91952E-5	5.23797E-4	1.51594E-6	37	1324	451	40723	over
GO:0016131	brassinosteroid metabolic process	6.91952E-5	5.36119E-4	1.55618E-6	6	26	482	42021	over
GO:0016128	phytosteroid metabolic process	6.91952E-5	5.36119E-4	1.55618E-6	6	26	482	42021	over
GO:0031418	L-ascorbic acid binding	9.96992E-5	7.97276E-4	2.44931E-6	7	46	481	42001	over
GO:0016746	transferase activity, transferring acyl groups	1.13904E-4	9.39268E-4	3.08265E-6	23	636	465	41411	over
GO:0006520	amino acid metabolic process	1.62079E-4	0.00137672	4.5242E-6	33	1168	455	40879	over
GO:0004053	arginase activity	2.32945E-4	0.00209431	5.95263E-6	3	1	485	42046	over
GO:0008783	agmatinase activity	2.32945E-4	0.00209431	5.95263E-6	3	1	485	42046	over
GO:0008152	metabolic process	2.58035E-4	0.00238397	7.07116E-6	290	20798	198	21249	over
GO:0016840	carbon-nitrogen lyase activity	3.1806E-4	0.00301701	8.85495E-6	7	57	481	41990	over
GO:0006542	glutamine biosynthetic process	3.50966E-4	0.00341607	1.04988E-5	5	21	483	42026	over
GO:0016620	oxidoreductase activity, acting on the aldehyde or oxo group of donors, NAD or NADP as acceptor	4.22344E-4	0.00421453	1.33018E-5	11	178	477	41869	over
GO:0009399	nitrogen fixation	5.13379E-4	0.00524832	1.53919E-5	5	23	483	42024	over
GO:0010268	brassinosteroid homeostasis	5.38267E-4	0.00563587	1.59419E-5	6	41	482	42006	over
GO:0016621	cinnamoyl-CoA reductase activity	5.66892E-4	0.00621642	1.84237E-5	5	24	483	42023	over

GO:0004356	glutamate-ammonia ligase activity	5.66892E-4	0.00621642	1.84237E-5	5	24	483	42023	over
GO:0004029	aldehyde dehydrogenase (NAD) activity	6.7065E-4	0.00751644	2.19005E-5	5	25	483	42022	over
GO:0045552	dihydrokaempferol 4-reductase activity	9.44675E-4	0.010805	2.92565E-5	3	3	485	42044	over
GO:0044271	nitrogen compound biosynthetic process	0.00132966	0.0155021	4.76745E-5	20	607	468	41440	over
GO:0044249	cellular biosynthetic process	0.00182552	0.0216682	6.84541E-5	82	4598	406	37449	over
GO:0044237	cellular metabolic process	0.00235303	0.0284133	8.29112E-5	242	17235	246	24812	over
GO:0006529	asparagine biosynthetic process	0.00236283	0.03025	8.77291E-5	4	17	484	42030	over
GO:0004066	asparagine synthase (glutamine-hydrolyzing) activity	0.00236283	0.03025	8.77291E-5	4	17	484	42030	over
GO:0006528	asparagine metabolic process	0.00236283	0.03025	8.77291E-5	4	17	484	42030	over
GO:0009855	determination of bilateral symmetry	0.00312917	0.0413643	1.27461E-4	4	19	484	42028	over
GO:0009799	determination of symmetry	0.00312917	0.0413643	1.27461E-4	4	19	484	42028	over
GO:0016903	oxidoreductase activity, acting on the aldehyde or oxo group of donors	0.00370435	0.0496599	1.3487E-4	11	233	477	41814	over
GO:0010073	meristem maintenance	0.00380247	0.0518429	1.46257E-4	5	39	483	42008	over
GO:0008652	amino acid biosynthetic process	0.00392375	0.0543797	1.51705E-4	17	510	471	41537	over
GO:0016706	oxidoreductase activity, acting on paired donors, with incorporation or reduction of molecular oxygen, 2-oxoglutarate as one donor, and incorporation of one atom each of oxygen into both donors	0.00447753	0.0628623	1.76149E-4	8	127	480	41920	over

GO:0007389	pattern specification process	0.0050731	0.0720981	2.17751E-4	10	206	478	41841	over
GO:0016705	oxidoreductase activity, acting on paired donors, with incorporation or reduction of molecular oxygen	0.00514504	0.0742739	2.28921E-4	13	336	475	41711	over
GO:0016813	hydrolase activity, acting on carbon-nitrogen (but not peptide) bonds, in linear amidines	0.00539701	0.0790096	2.31248E-4	3	8	485	42039	over
GO:0015291	secondary active transmembrane transporter activity	0.00656081	0.0966947	3.04216E-4	15	442	473	41605	over
GO:0004325	ferrochelatase activity	0.00698986	0.104249	3.05705E-4	3	9	485	42038	over
GO:0006783	heme biosynthetic process	0.00704367	0.108154	3.23764E-4	4	25	484	42022	over
GO:0045548	phenylalanine ammonia-lyase activity	0.00704367	0.108154	3.23764E-4	4	25	484	42022	over
GO:0016165	lipoxygenase activity	0.00754719	0.120439	3.70205E-4	4	26	484	42021	over
GO:0016884	carbon-nitrogen ligase activity, with glutamine as amido-N-donor	0.00754719	0.120439	3.70205E-4	4	26	484	42021	over
GO:0009067	aspartate family amino acid biosynthetic process	0.00754719	0.121552	3.76704E-4	8	143	480	41904	over
GO:0016874	ligase activity	0.00754719	0.122074	3.80297E-4	27	1115	461	40932	over
GO:0045430	chalcone isomerase activity	0.00869848	0.141208	3.91081E-4	2	1	486	42046	over
GO:0048878	chemical homeostasis	0.00985924	0.160549	4.72646E-4	9	187	479	41860	over
GO:0004730	pseudouridylate synthase activity	0.0105409	0.172829	4.97234E-4	3	11	485	42036	over
GO:0019842	vitamin binding	0.0106713	0.176966	5.28831E-4	12	321	476	41726	over
GO:0006559	L-phenylalanine catabolic process	0.0117095	0.194777	6.04202E-4	4	30	484	42017	over

GO:0009309	amine biosynthetic process	0.0125537	0.20974	6.42053E-4	17	581	471	41466	over
GO:0042168	heme metabolic process	0.0128745	0.217004	6.76023E-4	4	31	484	42016	over
GO:0015294	solute:cation symporter activity	0.0133857	0.227164	7.35976E-4	5	57	483	41990	over
GO:0010179	IAA-Ala conjugate hydrolase activity	0.0164982	0.27511	7.76209E-4	2	2	486	42045	over
GO:0009741	response to brassinosteroid stimulus	0.0175536	0.292987	9.01302E-4	7	126	481	41921	over
GO:0009074	aromatic amino acid family catabolic process	0.0183999	0.307899	9.27544E-4	4	34	484	42013	over
GO:0006118	electron transport	0.0184861	0.31446	9.90288E-4	33	1569	455	40478	over
GO:0016879	ligase activity, forming carbon-nitrogen bonds	0.0184861	0.315449	0.00100008	20	774	468	41273	over
GO:0016841	ammonia-lyase activity	0.0188192	0.323301	0.00102426	4	35	484	42012	over
GO:0020037	heme binding	0.0189367	0.328136	0.00106296	11	301	477	41746	over
GO:0009058	biosynthetic process	0.0210827	0.361124	0.00126708	94	5959	394	36088	over
GO:0005366	myo-inositol:hydrogen symporter activity	0.0236657	0.405894	0.00128385	2	3	486	42044	over
GO:0003867	4-aminobutyrate transaminase activity	0.0236657	0.405894	0.00128385	2	3	486	42044	over
GO:0009865	pollen tube adhesion	0.0236657	0.405894	0.00128385	2	3	486	42044	over
GO:0016844	strictosidine synthase activity	0.0258138	0.440591	0.00147963	3	17	485	42030	over
GO:0016843	amine-lyase activity	0.0258138	0.440591	0.00147963	3	17	485	42030	over

GO:0019439	aromatic compound catabolic process	0.0267303	0.455661	0.00161596	4	40	484	42007	over
GO:0016616	oxidoreductase activity, acting on the CH-OH group of donors, NAD or NADP as acceptor	0.0269387	0.461875	0.00169802	15	527	473	41520	over
GO:0006527	arginine catabolic process	0.0273484	0.474163	0.00171162	3	18	485	42029	over
GO:0047458	beta-pyrazolylalanine synthase activity	0.0273484	0.474163	0.00171162	3	18	485	42029	over
GO:0016829	lyase activity	0.0286285	0.493391	0.00188115	26	1184	462	40863	over
GO:0010178	IAA-amino acid conjugate hydrolase activity	0.0324288	0.540862	0.00191114	2	4	486	42043	over
GO:0009809	lignin biosynthetic process	0.0343789	0.565612	0.00207883	6	108	482	41939	over
GO:0016740	transferase activity	0.0348204	0.573966	0.00221263	98	6377	390	35670	over
GO:0046906	tetrapyrrole binding	0.0358116	0.591807	0.00234177	11	334	477	41713	over
GO:0015293	symporter activity	0.0358116	0.59364	0.00236953	9	238	479	41809	over
GO:0040008	regulation of growth	0.0358116	0.598824	0.00241117	4	45	484	42002	over
GO:0004033	aldo-keto reductase activity	0.0358116	0.598824	0.00241117	4	45	484	42002	over
GO:0045486	naringenin 3-dioxygenase activity	0.0371679	0.616052	0.00253919	3	21	485	42026	over
GO:0015269	calcium-activated potassium channel activity	0.0387462	0.658847	0.00265529	2	5	486	42042	over
GO:0005227	calcium activated cation channel activity	0.0387462	0.658847	0.00265529	2	5	486	42042	over
GO:0022839	ion gated channel activity	0.0387462	0.658847	0.00265529	2	5	486	42042	over

GO:0051259	protein oligomerization	0.0387462	0.658847	0.00265529	2	5	486	42042	over
GO:0051260	protein homooligomerization	0.0387462	0.658847	0.00265529	2	5	486	42042	over
GO:0004015	adenosylmethionine-8-amino-7-oxononanoate transaminase activity	0.0387462	0.658847	0.00265529	2	5	486	42042	over
GO:0009102	biotin biosynthetic process	0.0387462	0.658847	0.00265529	2	5	486	42042	over
GO:0006768	biotin metabolic process	0.0387462	0.658847	0.00265529	2	5	486	42042	over
GO:0044255	cellular lipid metabolic process	0.038994	0.664474	0.00275708	26	1219	462	40828	over
GO:0010224	response to UV-B	0.0396071	0.673438	0.00285943	5	79	483	41968	over
GO:0009063	amino acid catabolic process	0.0410011	0.692427	0.00310819	8	202	480	41845	over
GO:0009310	amine catabolic process	0.0410011	0.692427	0.00310819	8	202	480	41845	over
GO:0010014	meristem initiation	0.0415203	0.707152	0.00320693	3	23	485	42024	over
GO:0009820	alkaloid metabolic process	0.0415203	0.707152	0.00320693	3	23	485	42024	over
GO:0009821	alkaloid biosynthetic process	0.0415203	0.707152	0.00320693	3	23	485	42024	over
GO:0016614	oxidoreductase activity, acting on CH-OH group of donors	0.0415203	0.709885	0.00321749	15	565	473	41482	over
GO:0008202	steroid metabolic process	0.0415203	0.712319	0.00329232	6	119	482	41928	over
GO:0006558	L-phenylalanine metabolic process	0.0423809	0.722611	0.00344094	4	50	484	41997	over
GO:0015271	outward rectifier potassium channel activity	0.0452702	0.751527	0.00351355	2	6	486	42041	over

GO:0010279	indole-3-acetic acid amido synthetase	0.0452702	0.751527	0.00351355	2	6	486	42041	over
GO:0044270	nitrogen compound catabolic process	0.0479188	0.773701	0.00378892	8	209	480	41838	over

GOSSIP
Test-Set: tm1.yuc.10.up.txt
Tests for all terms in Gene Ontology whether it is enriched in a test group when compared to a reference group using Fisher's excact test with Multiple Testing.
Pub: Biological Profiling of Gene Groups utilizing Gene Ontology A Statistical Framework
Poster: GOSSIP: Biological Profiling of Gene Groups utilizing Gene Ontology
by Nils Blthgen, Karsten Brand, Hanspeter Herzel, Dieter Beule

GO Term	Name	FDR	FWER	single test p-Value	# in test group	# in reference group	# non annot test	# non annot reference group	Over/Under
GO:0045267	proton-transporting ATP synthase, catalytic core	2.28896E-6	1.83729E-6	5.55247E-9	14	54	1185	41282	over
GO:0005754	mitochondrial proton-transporting ATP synthase, catalytic core	2.28896E-6	1.83729E-6	5.55247E-9	14	54	1185	41282	over
GO:0000275	mitochondrial proton-transporting ATP synthase complex, catalytic core F(1)	2.28896E-6	2.44247E-6	8.24194E-9	14	56	1185	41280	over
GO:0031966	mitochondrial membrane	2.28896E-6	2.61843E-6	9.09598E-9	46	592	1153	40744	over
GO:0005753	mitochondrial proton-transporting ATP synthase complex	2.28896E-6	3.24825E-6	1.05295E-8	15	68	1184	41268	over
GO:0044455	mitochondrial membrane part	2.28896E-6	3.43343E-6	1.1299E-8	29	272	1170	41064	over
GO:0005740	mitochondrial envelope	4.85485E-6	8.49595E-6	2.51691E-8	47	635	1152	40701	over

GO:0044429	mitochondrial part	2.48551E-5	4.9874E-5	1.4117E-7	49	720	1150	40616	over
GO:0031967	organelle envelope	2.48551E-5	6.21242E-5	1.94297E-7	57	911	1142	40425	over
GO:0042631	cellular response to water deprivation	2.48551E-5	7.22113E-5	2.11044E-7	7	10	1192	41326	over
GO:0006119	oxidative phosphorylation	2.48551E-5	7.27709E-5	2.158E-7	32	373	1167	40963	over
GO:0031975	envelope	2.48551E-5	7.90098E-5	2.45533E-7	57	918	1142	40418	over
GO:0019866	organelle inner membrane	2.48551E-5	8.07757E-5	2.52397E-7	41	560	1158	40776	over
GO:0033178	proton-transporting two-sector ATPase complex, catalytic domain	3.3995E-5	1.18975E-4	3.60658E-7	16	106	1183	41230	over
GO:0005743	mitochondrial inner membrane	3.71353E-5	1.45873E-4	4.20363E-7	38	508	1161	40828	over
GO:0045261	proton-transporting ATP synthase complex, catalytic core F(1)	3.71353E-5	1.4853E-4	4.42099E-7	15	94	1184	41242	over
GO:0008324	cation transmembrane transporter activity	3.77634E-5	1.60482E-4	5.16049E-7	48	732	1151	40604	over
GO:0022890	inorganic cation transmembrane transporter activity	5.37558E-5	2.41872E-4	6.87534E-7	40	562	1159	40774	over
GO:0015078	hydrogen ion transmembrane transporter activity	6.5978E-5	3.13347E-4	8.55805E-7	34	440	1165	40896	over
GO:0045259	proton-transporting ATP synthase complex	7.13941E-5	3.56907E-4	9.57035E-7	16	115	1183	41221	over
GO:0015075	ion transmembrane transporter activity	7.60556E-5	3.99212E-4	1.12253E-6	55	918	1144	40418	over
GO:0048046	apoplast	2.70378E-4	0.00148597	4.05055E-6	16	130	1183	41206	over
GO:0015077	monovalent inorganic cation transmembrane transporter activity	3.05892E-4	0.00175733	4.85356E-6	34	479	1165	40857	over

GO:0046961	hydrogen ion transporting ATPase activity, rotational mechanism	5.87559E-4	0.00351915	9.37287E-6	21	228	1178	41108	over
GO:0016209	antioxidant activity	9.52641E-4	0.00593632	1.68525E-5	25	317	1174	41019	over
GO:0019829	cation-transporting ATPase activity	0.00127015	0.008222	2.10371E-5	23	281	1176	41055	over
GO:0006163	purine nucleotide metabolic process	0.00142948	0.00960257	2.25454E-5	25	323	1174	41013	over
GO:0009108	coenzyme biosynthetic process	0.00144747	0.0106662	2.60105E-5	27	368	1172	40968	over
GO:0015985	energy coupled proton transport, down electrochemical gradient	0.00144747	0.0107973	2.67693E-5	21	246	1178	41090	over
GO:0015986	ATP synthesis coupled proton transport	0.00144747	0.0107973	2.67693E-5	21	246	1178	41090	over
GO:0006753	nucleoside phosphate metabolic process	0.00157303	0.0133921	3.70969E-5	21	252	1178	41084	over
GO:0016469	proton-transporting two-sector ATPase complex	0.00157303	0.0133921	3.70969E-5	21	252	1178	41084	over
GO:0006754	ATP biosynthetic process	0.00157303	0.0133921	3.70969E-5	21	252	1178	41084	over
GO:0046034	ATP metabolic process	0.00157303	0.0133921	3.70969E-5	21	252	1178	41084	over
GO:0051188	cofactor biosynthetic process	0.00157303	0.0136698	3.80726E-5	33	510	1166	40826	over
GO:0016869	intramolecular transferase activity, transferring amino groups	0.00168749	0.0154881	4.01641E-5	4	4	1195	41332	over
GO:0042286	glutamate-1-semialdehyde 2,1-aminomutase activity	0.00168749	0.0154881	4.01641E-5	4	4	1195	41332	over
GO:0009152	purine ribonucleotide biosynthetic process	0.00190543	0.0179388	4.67794E-5	23	297	1176	41039	over
GO:0009150	purine ribonucleotide metabolic process	0.00193063	0.0186477	4.90719E-5	23	298	1176	41038	over

GO:0046933	hydrogen ion transporting ATP synthase activity, rotational mechanism	0.00206327	0.0204214	5.6248E-5	20	240	1179	41096	over
GO:0009206	purine ribonucleoside triphosphate biosynthetic process	0.00230988	0.0239621	6.89905E-5	21	264	1178	41072	over
GO:0009205	purine ribonucleoside triphosphate metabolic process	0.00230988	0.0239621	6.89905E-5	21	264	1178	41072	over
GO:0006122	mitochondrial electron transport, ubiquinol to cytochrome c	0.00232489	0.0248029	7.02984E-5	6	20	1193	41316	over
GO:0009199	ribonucleoside triphosphate metabolic process	0.00232489	0.0269479	7.25174E-5	21	265	1178	41071	over
GO:0009201	ribonucleoside triphosphate biosynthetic process	0.00232489	0.0269479	7.25174E-5	21	265	1178	41071	over
GO:0009145	purine nucleoside triphosphate biosynthetic process	0.00232489	0.0269479	7.25174E-5	21	265	1178	41071	over
GO:0009144	purine nucleoside triphosphate metabolic process	0.00232489	0.0269479	7.25174E-5	21	265	1178	41071	over
GO:0016020	membrane	0.00238157	0.0281745	7.64523E-5	341	9751	858	31585	over
GO:0006164	purine nucleotide biosynthetic process	0.00313089	0.0376275	8.94949E-5	23	311	1176	41025	over
GO:0009142	nucleoside triphosphate biosynthetic process	0.00325212	0.0398366	9.72524E-5	21	271	1178	41065	over
GO:0009260	ribonucleotide biosynthetic process	0.00326808	0.040955	1.02257E-4	23	314	1176	41022	over
GO:0009259	ribonucleotide metabolic process	0.00326808	0.0415955	1.06857E-4	23	315	1176	41021	over
GO:0004785	copper, zinc superoxide dismutase activity	0.00341746	0.0442718	1.1133E-4	5	13	1194	41323	over
GO:0009141	nucleoside triphosphate metabolic process	0.00364667	0.0488262	1.17648E-4	21	275	1178	41061	over
GO:0016491	oxidoreductase activity	0.00364667	0.0496043	1.21881E-4	147	3719	1052	37617	over

GO:0005739	mitochondrion	0.00364667	0.0497725	1.232E-4	337	9682	862	31654	over
GO:0016762	xyloglucan:xyloglucosyl transferase activity	0.00496174	0.0691931	1.88199E-4	10	78	1189	41258	over
GO:0016679	oxidoreductase activity, acting on diphenols and related substances as donors	0.00496174	0.0694191	1.88479E-4	13	128	1186	41208	over
GO:0005576	extracellular region	0.00636592	0.0896252	2.31949E-4	25	378	1174	40958	over
GO:0009165	nucleotide biosynthetic process	0.0074371	0.106817	2.71225E-4	26	405	1173	40931	over
GO:0042277	peptide binding	0.0074371	0.107223	2.71726E-4	8	52	1191	41284	over
GO:0042625	ATPase activity, coupled to transmembrane movement of ions	0.00866767	0.125718	3.43921E-4	24	366	1175	40970	over
GO:0000049	tRNA binding	0.0113483	0.163678	4.19237E-4	3	3	1196	41333	over
GO:0000234	phosphoethanolamine N-methyltransferase activity	0.0130472	0.190966	5.01539E-4	4	10	1195	41326	over
GO:0006811	ion transport	0.0130472	0.191056	5.01821E-4	47	951	1152	40385	over
GO:0042254	ribosome biogenesis and assembly	0.0142691	0.209785	5.89891E-4	32	573	1167	40763	over
GO:0015926	glucosidase activity	0.0148771	0.220579	6.3194E-4	6	32	1193	41304	over
GO:0031090	organelle membrane	0.0149012	0.22379	6.50522E-4	70	1595	1129	39741	over
GO:0006752	group transfer coenzyme metabolic process	0.0188876	0.278072	7.75249E-4	21	320	1178	41016	over
GO:0044444	cytoplasmic part	0.0189202	0.281884	7.91454E-4	754	24101	445	17235	over
GO:0004151	dihydroorotase activity	0.0210557	0.31186	7.9395E-4	2	0	1197	41336	over

GO:0008121	ubiquinol-cytochrome-c reductase activity	0.0235705	0.349617	9.44928E-4	8	64	1191	41272	over
GO:0016681	oxidoreductase activity, acting on diphenols and related substances as donors, cytochrome as acceptor	0.0235705	0.349617	9.44928E-4	8	64	1191	41272	over
GO:0019107	myristoyltransferase activity	0.0240492	0.362982	0.00101076	5	23	1194	41313	over
GO:0006656	phosphatidylcholine biosynthetic process	0.0240492	0.362982	0.00101076	5	23	1194	41313	over
GO:0015992	proton transport	0.0269293	0.402972	0.0011294	23	377	1176	40959	over
GO:0006818	hydrogen transport	0.0269293	0.406279	0.00116699	23	378	1176	40958	over
GO:0042775	organelle ATP synthesis coupled electron transport	0.0269293	0.408545	0.00117287	11	119	1188	41217	over
GO:0030529	ribonucleoprotein complex	0.0278391	0.422978	0.00123881	72	1694	1127	39642	over
GO:0005262	calcium channel activity	0.0302897	0.461496	0.00140134	4	14	1195	41322	over
GO:0016023	cytoplasmic membrane-bound vesicle	0.0302897	0.464373	0.0014546	180	4979	1019	36357	over
GO:0031410	cytoplasmic vesicle	0.0302897	0.464373	0.0014546	180	4979	1019	36357	over
GO:0031982	vesicle	0.0302897	0.470677	0.00147961	180	4981	1019	36355	over
GO:0031988	membrane-bound vesicle	0.0302897	0.470677	0.00147961	180	4981	1019	36355	over
GO:0004784	superoxide dismutase activity	0.0312741	0.489555	0.00162963	5	26	1194	41310	over
GO:0016721	oxidoreductase activity, acting on superoxide radicals as acceptor	0.0312741	0.489555	0.00162963	5	26	1194	41310	over
GO:0016859	cis-trans isomerase activity	0.0333396	0.519807	0.00172465	12	145	1187	41191	over

GO:0003755	peptidyl-prolyl cis-trans isomerase activity	0.0333396	0.519807	0.00172465	12	145	1187	41191	over
GO:0006801	superoxide metabolic process	0.034189	0.532711	0.00177142	6	40	1193	41296	over
GO:0042773	ATP synthesis coupled electron transport	0.0352532	0.54935	0.00189667	11	127	1188	41209	over
GO:0022892	substrate-specific transporter activity	0.0352532	0.551625	0.00192482	71	1696	1128	39640	over
GO:0009117	nucleotide metabolic process	0.0356806	0.562328	0.00201483	32	621	1167	40715	over
GO:0006812	cation transport	0.0356806	0.566734	0.00206059	39	806	1160	40530	over
GO:0008757	S-adenosylmethionine-dependent methyltransferase activity	0.0356806	0.567694	0.00207501	13	169	1186	41167	over
GO:0043492	ATPase activity, coupled to movement of substances	0.0387207	0.605231	0.00229547	28	524	1171	40812	over
GO:0042626	ATPase activity, coupled to transmembrane movement of substances	0.0387207	0.605231	0.00229547	28	524	1171	40812	over
GO:0000163	protein phosphatase type 1 activity	0.0444608	0.671012	0.00233716	2	1	1197	41335	over
GO:0003785	actin monomer binding	0.0444608	0.671012	0.00233716	2	1	1197	41335	over
GO:0010241	ent-kaurene oxidase activity	0.0444608	0.671012	0.00233716	2	1	1197	41335	over
GO:0009805	coumarin biosynthetic process	0.0444608	0.671012	0.00233716	2	1	1197	41335	over
GO:0006123	mitochondrial electron transport, cytochrome c to oxygen	0.0455811	0.686582	0.00248923	5	29	1194	41307	over
GO:0022891	substrate-specific transmembrane transporter activity	0.0455811	0.687313	0.00251426	58	1342	1141	39994	over
GO:0008654	phospholipid biosynthetic process	0.0483762	0.713209	0.0027515	10	114	1189	41222	over

GO:0016820	hydrolase activity, acting on acid anhydrides, catalyzing transmembrane movement of substances	0.0483762	0.717158	0.00280784	28	532	1171	40804	over
GO:0045277	respiratory chain complex IV	0.0483762	0.722588	0.00283757	5	30	1194	41306	over
GO:0005751	mitochondrial respiratory chain complex IV	0.0483762	0.722588	0.00283757	5	30	1194	41306	over
GO:0033554	cellular response to stress	0.0492834	0.7325	0.00302068	7	61	1192	41275	over

GOSSIP
Test-Set: tm1.yuc.10.down.txt
Tests for all terms in Gene Ontology whether it is enriched in a test group when compared to a reference group using Fisher's excact test with Multiple Testing.
Pub: Biological Profiling of Gene Groups utilizing Gene Ontology A Statistical Framework
Poster: GOSSIP: Biological Profiling of Gene Groups utilizing Gene Ontology
by Nils Blthgen, Karsten Brand, Hanspeter Herzel, Dieter Beule

GO Term	Name	FDR	FWER	single test p-Value	# in test group	# in reference group	# non annot test	# non annot reference group	Over/Under
GO:0003824	catalytic activity	1.38162E-7	3.45406E-8	3.55351E-11	503	18006	398	23628	over
GO:0019752	carboxylic acid metabolic process	5.98963E-7	4.37683E-7	1.39506E-9	89	2054	812	39580	over
GO:0006082	organic acid metabolic process	5.98963E-7	4.49222E-7	1.47817E-9	89	2055	812	39579	over
GO:0009813	flavonoid biosynthetic process	1.54679E-6	2.3952E-6	7.25467E-9	19	156	882	41478	over
GO:0016165	lipoxygenase activity	1.54679E-6	2.62742E-6	7.97664E-9	9	21	892	41613	over

GO:0006519	amino acid and derivative metabolic process	1.54679E-6	2.62742E-6	7.98813E-9	73	1610	828	40024	over
GO:0006541	glutamine metabolic process	1.54679E-6	2.70688E-6	8.42953E-9	13	64	888	41570	over
GO:0009812	flavonoid metabolic process	4.83563E-6	9.67121E-6	3.04399E-8	19	172	882	41462	over
GO:0009064	glutamine family amino acid metabolic process	1.80456E-5	4.42523E-5	1.36166E-7	17	152	884	41482	over
GO:0006528	asparagine metabolic process	1.80456E-5	5.41353E-5	1.67806E-7	7	14	894	41620	over
GO:0006529	asparagine biosynthetic process	1.80456E-5	5.41353E-5	1.67806E-7	7	14	894	41620	over
GO:0004066	asparagine synthase (glutamine-hydrolyzing) activity	1.80456E-5	5.41353E-5	1.67806E-7	7	14	894	41620	over
GO:0004335	galactokinase activity	4.66618E-5	1.51639E-4	4.72555E-7	7	17	894	41617	over
GO:0015299	solute:hydrogen antiporter activity	7.60165E-5	2.66022E-4	8.80403E-7	8	29	893	41605	over
GO:0009343	biotin carboxylase complex	9.43843E-5	3.62621E-4	9.83335E-7	4	1	897	41633	over
GO:0006012	galactose metabolic process	9.43843E-5	3.77466E-4	1.08112E-6	10	55	891	41579	over
GO:0009699	phenylpropanoid biosynthetic process	1.20309E-4	5.11184E-4	1.52198E-6	22	296	879	41338	over
GO:0006885	regulation of pH	1.44242E-4	6.48877E-4	1.90425E-6	6	13	895	41621	over
GO:0016210	naringenin-chalcone synthase activity	1.54249E-4	8.07881E-4	2.48765E-6	7	23	894	41611	over
GO:0016884	carbon-nitrogen ligase activity, with glutamine as amido-N-donor	1.54249E-4	8.07881E-4	2.48765E-6	7	23	894	41611	over
GO:0005506	iron ion binding	1.54249E-4	8.09477E-4	2.4979E-6	39	773	862	40861	over

GO:0048532	organization of an anatomical structure	1.99339E-4	0.00119381	3.22169E-6	10	63	891	41571	over
GO:0009933	meristem organization	1.99339E-4	0.00119381	3.22169E-6	10	63	891	41571	over
GO:0006631	fatty acid metabolic process	1.99339E-4	0.00119532	3.22541E-6	31	549	870	41085	over
GO:0016740	transferase activity	2.38595E-4	0.00149011	4.44497E-6	187	6288	714	35346	over
GO:0015298	solute:cation antiporter activity	2.68562E-4	0.00174413	5.02882E-6	8	38	893	41596	over
GO:0032787	monocarboxylic acid metabolic process	2.79571E-4	0.00188533	5.83257E-6	39	803	862	40831	over
GO:0051791	medium-chain fatty acid metabolic process	3.31236E-4	0.00231597	6.65267E-6	4	3	897	41631	over
GO:0019748	secondary metabolic process	3.35689E-4	0.00243079	7.05506E-6	39	810	862	40824	over
GO:0048507	meristem development	3.56176E-4	0.00266776	7.5867E-6	13	124	888	41510	over
GO:0016711	flavonoid 3'-monooxygenase activity	4.68023E-4	0.00362061	9.47363E-6	3	0	898	41634	over
GO:0006542	glutamine biosynthetic process	6.51001E-4	0.005347	1.42362E-5	6	20	895	41614	over
GO:0015385	sodium:hydrogen antiporter activity	6.51001E-4	0.00583833	1.51721E-5	5	11	896	41623	over
GO:0046835	carbohydrate phosphorylation	6.51001E-4	0.00583833	1.51721E-5	5	11	896	41623	over
GO:0005451	monovalent cation:proton antiporter activity	6.51001E-4	0.00583833	1.51721E-5	5	11	896	41623	over
GO:0016491	oxidoreductase activity	6.51001E-4	0.00584189	1.52288E-5	120	3746	781	37888	over
GO:0009698	phenylpropanoid metabolic process	7.35646E-4	0.00678164	1.87114E-5	23	377	878	41257	over

GO:0006575	amino acid derivative metabolic process	7.4417E-4	0.00704469	1.97787E-5	31	605	870	41029	over
GO:0048878	chemical homeostasis	7.7479E-4	0.00752575	2.12485E-5	15	181	886	41453	over
GO:0009374	biotin binding	7.77921E-4	0.00794199	2.24691E-5	6	22	895	41612	over
GO:0009399	nitrogen fixation	7.77921E-4	0.00794199	2.24691E-5	6	22	895	41612	over
GO:0006520	amino acid metabolic process	8.55846E-4	0.00894614	2.5567E-5	48	1153	853	40481	over
GO:0004356	glutamate-ammonia ligase activity	8.87726E-4	0.00961781	2.78238E-5	6	23	895	41611	over
GO:0008652	amino acid biosynthetic process	8.87726E-4	0.00971748	2.85286E-5	27	500	874	41134	over
GO:0015491	cation:cation antiporter activity	9.51423E-4	0.0106465	3.09968E-5	7	36	894	41598	over
GO:0019438	aromatic compound biosynthetic process	9.65903E-4	0.0110464	3.32109E-5	23	392	878	41242	over
GO:0004053	arginase activity	0.00117559	0.0142054	3.72947E-5	3	1	898	41633	over
GO:0008783	agmatinase activity	0.00117559	0.0142054	3.72947E-5	3	1	898	41633	over
GO:0044271	nitrogen compound biosynthetic process	0.00117559	0.0142978	3.7616E-5	30	597	871	41037	over
GO:0016879	ligase activity, forming carbon-nitrogen bonds	0.00141828	0.0175723	4.73878E-5	35	759	866	40875	over
GO:0016885	ligase activity, forming carbon-carbon bonds	0.00147974	0.0192868	5.02836E-5	6	26	895	41608	over
GO:0016421	CoA carboxylase activity	0.00147974	0.0192868	5.02836E-5	6	26	895	41608	over
GO:0006633	fatty acid biosynthetic process	0.00147974	0.0194157	5.137E-5	23	404	878	41230	over

GO:0009317	acetyl-CoA carboxylase complex	0.00162087	0.0219806	5.86101E-5	4	7	897	41627	over
GO:0016772	transferase activity, transferring phosphorus-containing groups	0.00162087	0.0220405	5.99917E-5	111	3518	790	38116	over
GO:0006118	electron transport	0.00166224	0.0230028	6.26775E-5	58	1544	843	40090	over
GO:0016301	kinase activity	0.00175953	0.0247617	6.78233E-5	98	3027	803	38607	over
GO:0009308	amine metabolic process	0.00183801	0.0262993	7.47152E-5	51	1310	850	40324	over
GO:0008395	steroid hydroxylase activity	0.00192711	0.0284614	8.23079E-5	5	17	896	41617	over
GO:0016773	phosphotransferase activity, alcohol group as acceptor	0.00192711	0.028493	8.28626E-5	84	2512	817	39122	over
GO:0016798	hydrolase activity, acting on glycosyl bonds	0.00221114	0.033158	9.11727E-5	36	818	865	40816	over
GO:0032441	pheophorbide a oxygenase activity	0.00241781	0.0369465	9.17617E-5	3	2	898	41632	over
GO:0009309	amine biosynthetic process	0.00241781	0.0383302	9.7177E-5	28	570	873	41064	over
GO:0004553	hydrolase activity, hydrolyzing O-glycosyl compounds	0.00241781	0.0391179	1.02818E-4	34	759	867	40875	over
GO:0003989	acetyl-CoA carboxylase activity	0.00241781	0.0396895	1.03341E-4	5	18	896	41616	over
GO:0009799	determination of symmetry	0.00241781	0.0396895	1.03341E-4	5	18	896	41616	over
GO:0009855	determination of bilateral symmetry	0.00241781	0.0396895	1.03341E-4	5	18	896	41616	over
GO:0009725	response to hormone stimulus	0.0024768	0.0412317	1.1054E-4	57	1543	844	40091	over
GO:0016053	organic acid biosynthetic process	0.0026994	0.0461415	1.23999E-4	23	430	878	41204	over

GO:0046394	carboxylic acid biosynthetic process	0.0026994	0.0461415	1.23999E-4	23	430	878	41204	over
GO:0003997	acyl-CoA oxidase activity	0.00275761	0.0477694	1.28265E-4	5	19	896	41615	over
GO:0015291	secondary active transmembrane transporter activity	0.00292022	0.0512068	1.40977E-4	23	434	878	41200	over
GO:0006807	nitrogen compound metabolic process	0.0029645	0.0526652	1.50036E-4	53	1420	848	40214	over
GO:0004075	biotin carboxylase activity	0.00336521	0.061263	1.68985E-4	4	10	897	41624	over
GO:0009063	amino acid catabolic process	0.00336521	0.0619385	1.73254E-4	14	196	887	41438	over
GO:0009310	amine catabolic process	0.00336521	0.0619385	1.73254E-4	14	196	887	41438	over
GO:0042398	amino acid derivative biosynthetic process	0.00423581	0.0795549	2.19437E-4	24	478	877	41156	over
GO:0016880	acid-ammonia (or amide) ligase activity	0.00423581	0.0802549	2.19714E-4	7	51	894	41583	over
GO:0016211	ammonia ligase activity	0.00423581	0.0802549	2.19714E-4	7	51	894	41583	over
GO:0016627	oxidoreductase activity, acting on the CH-CH group of donors	0.00455652	0.0893681	2.35838E-4	12	154	889	41480	over
GO:0006629	lipid metabolic process	0.00455652	0.0899543	2.40692E-4	51	1378	850	40256	over
GO:0016874	ligase activity	0.00455652	0.0903977	2.42436E-4	43	1099	858	40535	over
GO:0044270	nitrogen compound catabolic process	0.00455652	0.0905143	2.43058E-4	14	203	887	41431	over
GO:0006814	sodium ion transport	0.00455652	0.0912532	2.43161E-4	6	36	895	41598	over
GO:0016646	oxidoreductase activity, acting on the CH-NH group of donors, NAD or NADP as acceptor	0.00476431	0.0962871	2.72089E-4	7	53	894	41581	over

GO:0008535	cytochrome c oxidase complex assembly	0.00570409	0.117938	3.11102E-4	3	4	898	41630	over
GO:0015886	heme transport	0.00570409	0.117938	3.11102E-4	3	4	898	41630	over
GO:0015232	heme transporter activity	0.00570409	0.117938	3.11102E-4	3	4	898	41630	over
GO:0006783	heme biosynthetic process	0.00589407	0.122911	3.28309E-4	5	24	896	41610	over
GO:0006493	protein amino acid O-linked glycosylation	0.00639814	0.135464	3.81939E-4	4	13	897	41621	over
GO:0017004	cytochrome complex assembly	0.00639814	0.135464	3.81939E-4	4	13	897	41621	over
GO:0004029	aldehyde dehydrogenase (NAD) activity	0.00660439	0.14093	3.87139E-4	5	25	896	41609	over
GO:0006793	phosphorus metabolic process	0.00665825	0.143421	4.04692E-4	79	2452	822	39182	over
GO:0044255	cellular lipid metabolic process	0.00676596	0.147976	4.21319E-4	45	1200	856	40434	over
GO:0009739	response to gibberellin stimulus	0.00676596	0.14845	4.29179E-4	13	190	888	41444	over
GO:0009054	electron acceptor activity	0.00762701	0.171207	4.48213E-4	2	0	899	41634	over
GO:0050136	NADH dehydrogenase (quinone) activity	0.00762701	0.172027	4.49348E-4	8	77	893	41557	over
GO:0008137	NADH dehydrogenase (ubiquinone) activity	0.00762701	0.172027	4.49348E-4	8	77	893	41557	over
GO:0016645	oxidoreductase activity, acting on the CH-NH group of donors	0.00762701	0.172027	4.49348E-4	8	77	893	41557	over
GO:0010268	brassinosteroid homeostasis	0.00770799	0.175276	4.55041E-4	6	41	895	41593	over
GO:0047274	galactinol-sucrose galactosyltransferase activity	0.008399	0.196182	4.89914E-4	3	5	898	41629	over

GO:0051213	dioxygenase activity	0.008399	0.196182	4.89914E-4	3	5	898	41629	over
GO:0046369	galactose biosynthetic process	0.008399	0.196182	4.89914E-4	3	5	898	41629	over
GO:0050589	leucocyanidin oxygenase activity	0.008399	0.196182	4.89914E-4	3	5	898	41629	over
GO:0016128	phytosteroid metabolic process	0.00861382	0.204095	5.28276E-4	5	27	896	41607	over
GO:0016131	brassinosteroid metabolic process	0.00861382	0.204095	5.28276E-4	5	27	896	41607	over
GO:0043169	cation binding	0.00862253	0.205993	5.41821E-4	110	3695	791	37939	over
GO:0016706	oxidoreductase activity, acting on paired donors, with incorporation or reduction of molecular oxygen, 2-oxoglutarate as one donor, and incorporation of one atom each of oxygen into both donors	0.00951159	0.226496	6.31348E-4	10	125	891	41509	over
GO:0004672	protein kinase activity	0.00972809	0.232873	6.65684E-4	70	2151	831	39483	over
GO:0006725	aromatic compound metabolic process	0.00974978	0.235193	6.83258E-4	30	713	871	40921	over
GO:0016634	oxidoreductase activity, acting on the CH-CH group of donors, oxygen as acceptor	0.0100482	0.243347	7.10485E-4	6	45	895	41589	over
GO:0042221	response to chemical stimulus	0.0110069	0.26994	7.52672E-4	93	3054	808	38580	over
GO:0006857	oligopeptide transport	0.0110069	0.271282	7.63744E-4	8	84	893	41550	over
GO:0003954	NADH dehydrogenase activity	0.0110069	0.271282	7.63744E-4	8	84	893	41550	over
GO:0015833	peptide transport	0.0110069	0.271282	7.63744E-4	8	84	893	41550	over
GO:0042168	heme metabolic process	0.0114244	0.282031	8.08172E-4	5	30	896	41604	over

GO:0006796	phosphate metabolic process	0.0115327	0.286343	8.34344E-4	77	2439	824	39195	over
GO:0047458	beta-pyrazolylalanine synthase activity	0.0122687	0.303683	8.97904E-4	4	17	897	41617	over
GO:0009735	response to cytokinin stimulus	0.0125242	0.311074	9.33557E-4	10	132	891	41502	over
GO:0032502	developmental process	0.0127083	0.317011	9.81124E-4	89	2923	812	38711	over
GO:0008152	metabolic process	0.0128394	0.321873	0.00101331	493	20595	408	21039	over
GO:0051181	cofactor transport	0.0135804	0.339153	0.00101702	3	7	898	41627	over
GO:0015300	solute:solute antiporter activity	0.0148504	0.366622	0.00115606	8	90	893	41544	over
GO:0008810	cellulase activity	0.015126	0.374338	0.00118594	5	33	896	41601	over
GO:0009070	serine family amino acid biosynthetic process	0.0152989	0.380061	0.00123475	8	91	893	41543	over
GO:0045430	chalcone isomerase activity	0.0179212	0.431398	0.00132569	2	1	899	41633	over
GO:0009873	ethylene mediated signaling pathway	0.0181626	0.438677	0.0013411	7	71	894	41563	over
GO:0022414	reproductive process	0.0181626	0.44081	0.00135397	41	1130	860	40504	over
GO:0016813	hydrolase activity, acting on carbon-nitrogen (but not peptide) bonds, in linear amidines	0.018814	0.457476	0.00137643	3	8	898	41626	over
GO:0003968	RNA-directed RNA polymerase activity	0.018814	0.457476	0.00137643	3	8	898	41626	over
GO:0009888	tissue development	0.0189529	0.462474	0.00141341	20	423	881	41211	over
GO:0033013	tetrapyrrole metabolic process	0.0197896	0.482157	0.00154004	11	167	890	41467	over

GO:0006778	porphyrin metabolic process	0.0197896	0.482157	0.00154004	11	167	890	41467	over
GO:0019200	carbohydrate kinase activity	0.0210821	0.506554	0.0017658	9	120	892	41514	over
GO:0009257	10-formyltetrahydrofolate biosynthetic process	0.0212264	0.539237	0.00180642	3	9	898	41625	over
GO:0006545	glycine biosynthetic process	0.0212264	0.539237	0.00180642	3	9	898	41625	over
GO:0004146	dihydrofolate reductase activity	0.0212264	0.539237	0.00180642	3	9	898	41625	over
GO:0006231	dTMP biosynthetic process	0.0212264	0.539237	0.00180642	3	9	898	41625	over
GO:0009157	deoxyribonucleoside monophosphate biosynthetic process	0.0212264	0.539237	0.00180642	3	9	898	41625	over
GO:0004799	thymidylate synthase activity	0.0212264	0.539237	0.00180642	3	9	898	41625	over
GO:0009162	deoxyribonucleoside monophosphate metabolic process	0.0212264	0.539237	0.00180642	3	9	898	41625	over
GO:0009256	10-formyltetrahydrofolate metabolic process	0.0212264	0.539237	0.00180642	3	9	898	41625	over
GO:0009176	pyrimidine deoxyribonucleoside monophosphate metabolic process	0.0212264	0.539237	0.00180642	3	9	898	41625	over
GO:0046073	dTMP metabolic process	0.0212264	0.539237	0.00180642	3	9	898	41625	over
GO:0042083	5,10-methylenetetrahydrofolate-dependent methyltransferase activity	0.0212264	0.539237	0.00180642	3	9	898	41625	over
GO:0009177	pyrimidine deoxyribonucleoside monophosphate biosynthetic process	0.0212264	0.539237	0.00180642	3	9	898	41625	over
GO:0000003	reproduction	0.0220477	0.559484	0.00201534	41	1156	860	40478	over
GO:0010014	meristem initiation	0.0220477	0.572144	0.00206237	4	22	897	41612	over

GO:0010084	specification of organ axis polarity	0.0220477	0.572144	0.00206237	4	22	897	41612	over
GO:0005983	starch catabolic process	0.0220477	0.572144	0.00206237	4	22	897	41612	over
GO:0009944	polarity specification of adaxial/abaxial axis	0.0220477	0.572144	0.00206237	4	22	897	41612	over
GO:0009955	adaxial/abaxial pattern formation	0.0220477	0.572144	0.00206237	4	22	897	41612	over
GO:0009943	adaxial/abaxial axis specification	0.0220477	0.572144	0.00206237	4	22	897	41612	over
GO:0065001	specification of axis polarity	0.0220477	0.572144	0.00206237	4	22	897	41612	over
GO:0006470	protein amino acid dephosphorylation	0.0225089	0.583525	0.0021794	14	258	887	41376	over
GO:0019842	vitamin binding	0.0225089	0.584382	0.00220432	16	317	885	41317	over
GO:0010073	meristem maintenance	0.0242115	0.61344	0.00231183	5	39	896	41595	over
GO:0042592	homeostatic process	0.0253161	0.634065	0.00238971	17	350	884	41284	over
GO:0044237	cellular metabolic process	0.0253161	0.636592	0.00244298	412	17065	489	24569	over
GO:0048856	anatomical structure development	0.0253161	0.636811	0.00244669	68	2190	833	39444	over
GO:0015293	symporter activity	0.0257061	0.644726	0.00254598	13	234	888	41400	over
GO:0019594	mannitol metabolic process	0.0280001	0.696445	0.00261409	2	2	899	41632	over
GO:0019593	mannitol biosynthetic process	0.0280001	0.696445	0.00261409	2	2	899	41632	over
GO:0046173	polyol biosynthetic process	0.0280001	0.696445	0.00261409	2	2	899	41632	over

GO:0047268	galactinol-raffinose galactosyltransferase activity	0.0280001	0.696445	0.00261409	2	2	899	41632	over
GO:0019406	hexitol biosynthetic process	0.0280001	0.696445	0.00261409	2	2	899	41632	over
GO:0006059	hexitol metabolic process	0.0280001	0.696445	0.00261409	2	2	899	41632	over
GO:0010325	raffinose family oligosaccharide biosynthetic process	0.0280001	0.696445	0.00261409	2	2	899	41632	over
GO:0019401	alditol biosynthetic process	0.0280001	0.696445	0.00261409	2	2	899	41632	over
GO:0019400	alditol metabolic process	0.0280001	0.696445	0.00261409	2	2	899	41632	over
GO:0046914	transition metal ion binding	0.0280001	0.698872	0.0026713	82	2750	819	38884	over
GO:0009719	response to endogenous stimulus	0.0280001	0.700086	0.00268756	69	2238	832	39396	over
GO:0004473	malate dehydrogenase (oxaloacetate-decarboxylating) (NADP+) activity	0.0284376	0.709835	0.00273157	4	24	897	41610	over
GO:0009251	glucan catabolic process	0.0284376	0.709835	0.00273157	4	24	897	41610	over
GO:0004730	pseudouridylate synthase activity	0.0301689	0.732964	0.00289585	3	11	898	41623	over
GO:0050801	ion homeostasis	0.0301689	0.734925	0.00293312	9	130	892	41504	over
GO:0004721	phosphoprotein phosphatase activity	0.0302283	0.737608	0.0029802	21	485	880	41149	over
GO:0016311	dephosphorylation	0.0309559	0.74983	0.00311759	14	269	887	41365	over
GO:0042440	pigment metabolic process	0.0309559	0.74983	0.00311759	14	269	887	41365	over
GO:0015994	chlorophyll metabolic process	0.0316675	0.759587	0.0033818	9	133	892	41501	over

GO:0015071	protein phosphatase type 2C activity	0.0319429	0.76444	0.00340948	12	214	889	41420	over
GO:0051539	4 iron, 4 sulfur cluster binding	0.0320799	0.767767	0.00345146	7	85	894	41549	over
GO:0009065	glutamine family amino acid catabolic process	0.0330925	0.780057	0.00353593	4	26	897	41608	over
GO:0017119	Golgi transport complex	0.0345085	0.795632	0.00356317	3	12	898	41622	over
GO:0007275	multicellular organismal development	0.034864	0.800694	0.00369536	67	2191	834	39443	over
GO:0009084	glutamine family amino acid biosynthetic process	0.0355747	0.808852	0.00388973	7	87	894	41547	over
GO:0009723	response to ethylene stimulus	0.0355856	0.810642	0.0039435	11	190	890	41444	over
GO:0046483	heterocycle metabolic process	0.0370768	0.825032	0.00421359	18	403	883	41231	over
GO:0000914	phragmoplast formation	0.0399459	0.856012	0.00429564	2	3	899	41631	over
GO:0003867	4-aminobutyrate transaminase activity	0.0399459	0.856012	0.00429564	2	3	899	41631	over
GO:0031032	actomyosin structure organization and biogenesis	0.0399459	0.856012	0.00429564	2	3	899	41631	over
GO:0009865	pollen tube adhesion	0.0399459	0.856012	0.00429564	2	3	899	41631	over
GO:0048768	root hair cell tip growth	0.0399459	0.856012	0.00429564	2	3	899	41631	over
GO:0000912	cytokinesis, formation of actomyosin apparatus	0.0399459	0.856012	0.00429564	2	3	899	41631	over
GO:0009130	pyrimidine nucleoside monophosphate biosynthetic process	0.041023	0.866122	0.00431689	3	13	898	41621	over
GO:0009129	pyrimidine nucleoside monophosphate metabolic process	0.041023	0.866122	0.00431689	3	13	898	41621	over

GO:0044247	cellular polysaccharide catabolic process	0.0411074	0.868038	0.00436895	7	89	894	41545	over
GO:0009798	axis specification	0.0413153	0.870726	0.00442976	5	46	896	41588	over
GO:0048367	shoot development	0.0424628	0.88061	0.00471068	19	440	882	41194	over
GO:0022621	shoot system development	0.0424628	0.88061	0.00471068	19	440	882	41194	over
GO:0009528	plastid inner membrane	0.0424628	0.883554	0.00481709	5	47	896	41587	over
GO:0009706	chloroplast inner membrane	0.0424628	0.883554	0.00481709	5	47	896	41587	over
GO:0016655	oxidoreductase activity, acting on NADH or NADPH, quinone or similar compound as acceptor	0.0424628	0.884188	0.00484465	9	141	892	41493	over
GO:0004124	cysteine synthase activity	0.0432824	0.891415	0.00502204	4	29	897	41605	over
GO:0009067	aspartate family amino acid biosynthetic process	0.0432824	0.891769	0.00505747	9	142	892	41492	over
GO:0044262	cellular carbohydrate metabolic process	0.0432824	0.892461	0.00508812	40	1185	861	40449	over
GO:0009221	pyrimidine deoxyribonucleotide biosynthetic process	0.0440996	0.898028	0.00516009	3	14	898	41620	over
GO:0031418	L-ascorbic acid binding	0.0446315	0.9019	0.00522784	5	48	896	41586	over
GO:0009066	aspartate family amino acid metabolic process	0.0448469	0.904076	0.00537898	13	257	888	41377	over
GO:0009908	flower development	0.0450143	0.905978	0.00551508	19	447	882	41187	over
GO:0006120	mitochondrial electron transport, NADH to ubiquinone	0.0460556	0.912308	0.00566275	5	49	896	41585	over
GO:0065007	biological regulation	0.0460556	0.913012	0.0057332	123	4524	778	37110	over

GO:0016791	phosphoric monoester hydrolase activity	0.0469041	0.917812	0.00605365	27	725	874	40909	over
GO:0045271	respiratory chain complex I	0.047292	0.925011	0.00609554	3	15	898	41619	over
GO:0009263	deoxyribonucleotide biosynthetic process	0.047292	0.925011	0.00609554	3	15	898	41619	over
GO:0009788	negative regulation of abscisic acid mediated signaling	0.047292	0.925011	0.00609554	3	15	898	41619	over
GO:0051184	cofactor transporter activity	0.047292	0.925011	0.00609554	3	15	898	41619	over
GO:0005747	mitochondrial respiratory chain complex I	0.047292	0.925011	0.00609554	3	15	898	41619	over
GO:0030964	NADH dehydrogenase complex (quinone)	0.047292	0.925011	0.00609554	3	15	898	41619	over
GO:0032501	multicellular organismal process	0.0476723	0.927429	0.00616967	70	2363	831	39271	over

GOSSIP
Test-Set: tm1.yuc.20.up.txt
Tests for all terms in Gene Ontology whether it is enriched in a test group when compared to a reference group using Fisher's excact test with Multiple Testing.
Pub: Biological Profiling of Gene Groups utilizing Gene Ontology A Statistical Framework
Poster: GOSSIP: Biological Profiling of Gene Groups utilizing Gene Ontology
by Nils Blthgen, Karsten Brand, Hanspeter Herzel, Dieter Beule

GO Term	Name	FDR	FWER	single test p-Value	# in test group	# in reference group	# non annot test	# non annot reference group	Over/Under
GO:0042455	ribonucleoside biosynthetic process	3.1721E-4	5.54963E-4	1.64304E-6	5	10	609	41911	over
GO:0046132	pyrimidine ribonucleoside biosynthetic process	3.1721E-4	5.54963E-4	1.64304E-6	5	10	609	41911	over

GO:0046108	uridine metabolic process	3.1721E-4	5.54963E-4	1.64304E-6	5	10	609	41911	over
GO:0046109	uridine biosynthetic process	3.1721E-4	5.54963E-4	1.64304E-6	5	10	609	41911	over
GO:0046134	pyrimidine nucleoside biosynthetic process	3.1721E-4	5.54963E-4	1.64304E-6	5	10	609	41911	over
GO:0009052	pentose-phosphate shunt, non-oxidative branch	3.1721E-4	5.54963E-4	1.64304E-6	5	10	609	41911	over
GO:0009163	nucleoside biosynthetic process	3.1721E-4	5.54963E-4	1.64304E-6	5	10	609	41911	over
GO:0042286	glutamate-1-semialdehyde 2,1-aminomutase activity	4.13563E-4	9.30084E-4	2.87449E-6	4	4	610	41917	over
GO:0016869	intramolecular transferase activity, transferring amino groups	4.13563E-4	9.30084E-4	2.87449E-6	4	4	610	41917	over
GO:0005262	calcium channel activity	6.1776E-4	0.00154321	4.52238E-6	5	13	609	41908	over
GO:0046131	pyrimidine ribonucleoside metabolic process	0.00122301	0.00335764	1.03622E-5	5	16	609	41905	over
GO:0000234	phosphoethanolamine N-methyltransferase activity	0.00456255	0.0135944	3.83636E-5	4	10	610	41911	over
GO:0006213	pyrimidine nucleoside metabolic process	0.00745852	0.0239489	6.51836E-5	5	25	609	41896	over
GO:0042631	cellular response to water deprivation	0.00887441	0.0305832	8.8126E-5	4	13	610	41908	over
GO:0019825	oxygen binding	0.0102417	0.0376786	1.04753E-4	5	28	609	41893	over
GO:0005245	voltage-gated calcium channel activity	0.0140648	0.0547065	1.58807E-4	3	5	611	41916	over
GO:0004872	receptor activity	0.0154794	0.0636711	1.90546E-4	19	491	595	41430	over
GO:0032502	developmental process	0.0234219	0.108156	3.05035E-4	67	2945	547	38976	over

GO:0019720	Mo-molybdopterin cofactor metabolic process	0.0234219	0.120871	3.33036E-4	3	7	611	41914	over
GO:0043545	molybdopterin cofactor metabolic process	0.0234219	0.120871	3.33036E-4	3	7	611	41914	over
GO:0032324	molybdopterin cofactor biosynthetic process	0.0234219	0.120871	3.33036E-4	3	7	611	41914	over
GO:0006777	Mo-molybdopterin cofactor biosynthetic process	0.0234219	0.120871	3.33036E-4	3	7	611	41914	over
GO:0016209	antioxidant activity	0.0314862	0.167543	5.17299E-4	14	328	600	41593	over
GO:0009734	auxin mediated signaling pathway	0.0314862	0.172151	5.3981E-4	10	183	604	41738	over
GO:0010088	phloem histogenesis	0.0317595	0.198043	5.9755E-4	3	9	611	41912	over
GO:0008271	secondary active sulfate transmembrane transporter activity	0.0317595	0.198043	5.9755E-4	3	9	611	41912	over
GO:0004871	signal transducer activity	0.0317595	0.199346	5.99804E-4	24	766	590	41155	over
GO:0060089	molecular transducer activity	0.0317595	0.199346	5.99804E-4	24	766	590	41155	over
GO:0003785	actin monomer binding	0.0346633	0.225981	6.18132E-4	2	1	612	41920	over
GO:0019107	myristoyltransferase activity	0.0346633	0.237525	6.68488E-4	4	24	610	41897	over
GO:0006656	phosphatidylcholine biosynthetic process	0.0346633	0.237525	6.68488E-4	4	24	610	41897	over
GO:0004372	glycine hydroxymethyltransferase activity	0.0346633	0.242683	6.96186E-4	5	44	609	41877	over
GO:0004497	monooxygenase activity	0.0346633	0.248729	7.43887E-4	14	341	600	41580	over
GO:0042559	pteridine and derivative biosynthetic process	0.0350166	0.270334	7.68499E-4	3	10	611	41911	over

GO:0015116	sulfate transmembrane transporter activity	0.0350166	0.270334	7.68499E-4	3	10	611	41911	over
GO:0042558	pteridine and derivative metabolic process	0.0350166	0.270334	7.68499E-4	3	10	611	41911	over
GO:0006816	calcium ion transport	0.0381156	0.300598	9.41184E-4	6	72	608	41849	over
GO:0050896	response to stimulus	0.0381156	0.303804	9.64607E-4	111	5667	503	36254	over
GO:0007585	respiratory gaseous exchange	0.0388748	0.315493	9.67627E-4	3	11	611	41910	over
GO:0006982	response to lipid hydroperoxide	0.044793	0.368192	0.0011966	3	12	611	41909	over
GO:0033194	response to hydroperoxide	0.044793	0.368192	0.0011966	3	12	611	41909	over

GOSSIP
Test-Set: tm1.yuc.20.down.txt
Tests for all terms in Gene Ontology whether it is enriched in a test group when compared to a reference group using Fisher's excact test with Multiple Testing.
Pub: Biological Profiling of Gene Groups utilizing Gene Ontology A Statistical Framework
Poster: GOSSIP: Biological Profiling of Gene Groups utilizing Gene Ontology
by Nils Blthgen, Karsten Brand, Hanspeter Herzel, Dieter Beule

GO Term	Name	FDR	FWER	single test p-Value	# in test group	# in reference group	# non annot test	# non annot reference group	Over/Under
GO:0009813	flavonoid biosynthetic process	3.05368E-8	2.42797E-8	0.0	21	154	494	41866	over
GO:0047461	(+)-delta-cadinene synthase activity	3.05368E-8	2.42797E-8	0.0	8	8	507	42012	over
GO:0009812	flavonoid metabolic process	3.05368E-8	2.59604E-8	6.70616E-12	21	170	494	41850	over

GO:0016210	naringenin-chalcone synthase activity	3.05368E-8	3.05368E-8	2.97372E-11	13	17	502	42003	over
GO:0009699	phenylpropanoid biosynthetic process	3.61765E-8	4.52207E-8	1.05016E-10	22	296	493	41724	over
GO:0009698	phenylpropanoid metabolic process	5.89349E-8	8.84024E-8	2.14192E-10	24	376	491	41644	over
GO:0003824	catalytic activity	1.64109E-7	2.87191E-7	1.01872E-9	292	18217	223	23803	over
GO:0019438	aromatic compound biosynthetic process	1.44116E-6	2.88231E-6	1.07387E-8	22	393	493	41627	over
GO:0006575	amino acid derivative metabolic process	3.53814E-6	7.96078E-6	2.56268E-8	27	609	488	41411	over
GO:0016838	carbon-oxygen lyase activity, acting on phosphates	5.01177E-6	1.25294E-5	4.36852E-8	8	35	507	41985	over
GO:0019748	secondary metabolic process	7.86787E-6	2.16364E-5	7.31113E-8	31	818	484	41202	over
GO:0006519	amino acid and derivative metabolic process	1.30418E-5	3.91247E-5	1.24349E-7	47	1636	468	40384	over
GO:0006725	aromatic compound metabolic process	1.49612E-5	4.86228E-5	1.6703E-7	28	715	487	41305	over
GO:0016746	transferase activity, transferring acyl groups	1.6217E-5	5.87874E-5	1.93426E-7	26	633	489	41387	over
GO:0008395	steroid hydroxylase activity	1.6217E-5	6.0812E-5	1.93753E-7	6	16	509	42004	over
GO:0042398	amino acid derivative biosynthetic process	2.01051E-5	8.04171E-5	2.95333E-7	22	480	493	41540	over
GO:0008415	acyltransferase activity	7.70419E-5	3.27375E-4	1.07007E-6	23	563	492	41457	over
GO:0016747	transferase activity, transferring groups other than amino-acyl groups	9.47297E-5	4.26193E-4	1.42329E-6	23	573	492	41447	over
GO:0016711	flavonoid 3'-monooxygenase activity	1.29896E-4	6.16818E-4	1.76469E-6	3	0	512	42020	over

GO:0016131	brassinosteroid metabolic process	1.35301E-4	7.10077E-4	2.12277E-6	6	26	509	41994	over
GO:0016128	phytosteroid metabolic process	1.35301E-4	7.10077E-4	2.12277E-6	6	26	509	41994	over
GO:0004489	methylenetetrahydrofolate reductase (NADPH) activity	0.00124742	0.00683735	1.93106E-5	4	10	511	42010	over
GO:0010268	brassinosteroid homeostasis	0.00128287	0.00734935	2.15707E-5	6	41	509	41979	over
GO:0000910	cytokinesis	0.00148872	0.00889256	2.65325E-5	8	90	507	41930	over
GO:0016740	transferase activity	0.00257508	0.0159655	4.78377E-5	112	6363	403	35657	over
GO:0004675	transmembrane receptor protein serine/threonine kinase activity	0.00372669	0.0239326	7.1264E-5	4	15	511	42005	over
GO:0051301	cell division	0.00400829	0.0266934	8.32127E-5	11	208	504	41812	over
GO:0016297	acyl-[acyl-carrier-protein] hydrolase activity	0.00402951	0.0287915	8.82289E-5	4	16	511	42004	over
GO:0010281	acyl-ACP thioesterase activity	0.00402951	0.0287915	8.82289E-5	4	16	511	42004	over
GO:0005245	voltage-gated calcium channel activity	0.00443862	0.0329974	9.44487E-5	3	5	512	42015	over
GO:0009845	seed germination	0.00443862	0.0338146	9.98197E-5	7	81	508	41939	over
GO:0016835	carbon-oxygen lyase activity	0.00483278	0.0379247	1.13262E-4	15	380	500	41640	over
GO:0008202	steroid metabolic process	0.00704402	0.0564575	1.49294E-4	8	117	507	41903	over
GO:0004320	oleoyl-[acyl-carrier-protein] hydrolase activity	0.0154192	0.122841	3.57879E-4	3	9	512	42011	over
GO:0004312	fatty-acid synthase activity	0.0161536	0.131813	4.03029E-4	6	73	509	41947	over

GO:0045430	chalcone isomerase activity	0.018723	0.15508	4.35414E-4	2	1	513	42019	over
GO:0005484	SNAP receptor activity	0.0244367	0.202321	5.83755E-4	4	28	511	41992	over
GO:0004418	hydroxymethylbilane synthase activity	0.0367901	0.29498	8.63834E-4	2	2	513	42018	over
GO:0004672	protein kinase activity	0.0413125	0.331573	0.00103158	44	2177	471	39843	over
GO:0000096	sulfur amino acid metabolic process	0.0413362	0.350103	0.00112691	9	201	506	41819	over
GO:0000325	vacuole, cell cycle independent morphology	0.0413362	0.362662	0.00119395	6	91	509	41929	over
GO:0009741	response to brassinosteroid stimulus	0.0413362	0.365481	0.00122894	7	126	508	41894	over
GO:0022832	voltage-gated channel activity	0.0413362	0.374354	0.00124898	4	35	511	41985	over
GO:0022843	voltage-gated cation channel activity	0.0413362	0.374354	0.00124898	4	35	511	41985	over
GO:0005244	voltage-gated ion channel activity	0.0413362	0.374354	0.00124898	4	35	511	41985	over
GO:0005262	calcium channel activity	0.0413362	0.384762	0.00125757	3	15	512	42005	over
GO:0009773	photosynthetic electron transport in photosystem I	0.0413362	0.384762	0.00125757	3	15	512	42005	over
GO:0047209	coniferyl-alcohol glucosyltransferase activity	0.0441071	0.441471	0.00142817	2	3	513	42017	over
GO:0048768	root hair cell tip growth	0.0441071	0.441471	0.00142817	2	3	513	42017	over
GO:0000914	phragmoplast formation	0.0441071	0.441471	0.00142817	2	3	513	42017	over
GO:0031032	actomyosin structure organization and biogenesis	0.0441071	0.441471	0.00142817	2	3	513	42017	over

GO:0000912	cytokinesis, formation of actomyosin apparatus	0.0441071	0.441471	0.00142817	2	3	513	42017	over
GO:0004497	monooxygenase activity	0.0441071	0.442606	0.001454	12	343	503	41677	over
GO:0004337	geranyltranstransferase activity	0.0447861	0.453751	0.00148001	3	16	512	42004	over
GO:0004659	prenyltransferase activity	0.0460947	0.46947	0.0016177	5	65	510	41955	over
GO:0004559	alpha-mannosidase activity	0.0471412	0.492675	0.00172561	3	17	512	42003	over
GO:0005057	receptor signaling protein activity	0.0471412	0.498232	0.00179356	6	99	509	41921	over
GO:0004702	receptor signaling protein serine/threonine kinase activity	0.0471412	0.498232	0.00179356	6	99	509	41921	over
GO:0000036	acyl carrier activity	0.0471412	0.501137	0.00180398	4	39	511	41981	over
GO:0004888	transmembrane receptor activity	0.0478393	0.512121	0.00193839	7	137	508	41883	over

GOSSIP
Test-Set: tm1.yuc.25.up.txt
Tests for all terms in Gene Ontology whether it is enriched in a test group when compared to a reference group using Fisher's excact test with Multiple Testing.
Pub: Biological Profiling of Gene Groups utilizing Gene Ontology A Statistical Framework
Poster: GOSSIP: Biological Profiling of Gene Groups utilizing Gene Ontology
by Nils Blthgen, Karsten Brand, Hanspeter Herzel, Dieter Beule

GO Term	Name	FDR	FWER	single test p-Value	# in test group	# in reference group	# non annot test	# non annot reference group	Over/Under
GO:0009607	response to biotic stimulus	2.34657E-8	3.77368E-8	0.0	81	1152	1119	40183	over

GO:0009052	pentose-phosphate shunt, non-oxidative branch	2.34657E-8	4.69314E-8	3.40069E-11	9	6	1191	41329	over
GO:0046109	uridine biosynthetic process	2.34657E-8	4.69314E-8	3.40069E-11	9	6	1191	41329	over
GO:0042455	ribonucleoside biosynthetic process	2.34657E-8	4.69314E-8	3.40069E-11	9	6	1191	41329	over
GO:0046108	uridine metabolic process	2.34657E-8	4.69314E-8	3.40069E-11	9	6	1191	41329	over
GO:0009163	nucleoside biosynthetic process	2.34657E-8	4.69314E-8	3.40069E-11	9	6	1191	41329	over
GO:0046132	pyrimidine ribonucleoside biosynthetic process	2.34657E-8	4.69314E-8	3.40069E-11	9	6	1191	41329	over
GO:0046134	pyrimidine nucleoside biosynthetic process	2.34657E-8	4.69314E-8	3.40069E-11	9	6	1191	41329	over
GO:0045735	nutrient reservoir activity	2.80565E-8	6.31271E-8	9.31431E-11	20	99	1180	41236	over
GO:0050832	defense response to fungus	1.12241E-7	2.80602E-7	8.29915E-10	22	137	1178	41198	over
GO:0051707	response to other organism	1.78526E-7	4.90946E-7	1.46062E-9	61	869	1139	40466	over
GO:0046131	pyrimidine ribonucleoside metabolic process	2.45482E-7	7.36447E-7	2.29431E-9	9	12	1191	41323	over
GO:0009620	response to fungus	5.82865E-7	1.89431E-6	6.31163E-9	26	215	1174	41120	over
GO:0051704	multi-organism process	7.57265E-7	2.65042E-6	9.50875E-9	65	1008	1135	40327	over
GO:0031347	regulation of defense response	3.51135E-6	1.31849E-5	4.01212E-8	10	26	1190	41309	over
GO:0009751	response to salicylic acid stimulus	3.51135E-6	1.40453E-5	4.15565E-8	27	255	1173	41080	over
GO:0006213	pyrimidine nucleoside metabolic process	6.65532E-6	2.82847E-5	9.21254E-8	9	21	1191	41314	over

GO:0050896	response to stimulus	2.65357E-5	1.19403E-4	3.64123E-7	224	5554	976	35781	over
GO:0009414	response to water deprivation	3.393E-5	1.61155E-4	5.1868E-7	32	389	1168	40946	over
GO:0048583	regulation of response to stimulus	3.6067E-5	1.88273E-4	5.81909E-7	11	47	1189	41288	over
GO:0006952	defense response	3.6067E-5	1.89334E-4	5.92553E-7	52	827	1148	40508	over
GO:0009415	response to water	4.89574E-5	2.69229E-4	7.35323E-7	33	416	1167	40919	over
GO:0009703	nitrate reductase (NADH) activity	5.86663E-5	3.51936E-4	9.24635E-7	5	3	1195	41332	over
GO:0046857	oxidoreductase activity, acting on other nitrogenous compounds as donors, with NAD or NADP as acceptor	5.86663E-5	3.51936E-4	9.24635E-7	5	3	1195	41332	over
GO:0042742	defense response to bacterium	1.39724E-4	8.72894E-4	2.57081E-6	21	208	1179	41127	over
GO:0020037	heme binding	1.95752E-4	0.00127158	3.50425E-6	25	287	1175	41048	over
GO:0004497	monooxygenase activity	1.99443E-4	0.00134533	3.8695E-6	27	328	1173	41007	over
GO:0009685	gibberellin metabolic process	2.15599E-4	0.00156187	4.30412E-6	7	18	1193	41317	over
GO:0016101	diterpenoid metabolic process	2.15599E-4	0.00156187	4.30412E-6	7	18	1193	41317	over
GO:0005576	extracellular region	2.39232E-4	0.00179263	5.09293E-6	29	374	1171	40961	over
GO:0042044	fluid transport	3.16825E-4	0.00253139	7.30999E-6	8	29	1192	41306	over
GO:0006833	water transport	3.16825E-4	0.00253139	7.30999E-6	8	29	1192	41306	over
GO:0016661	oxidoreductase activity, acting on other nitrogenous compounds as donors	3.24351E-4	0.00267232	7.5686E-6	7	20	1193	41315	over

GO:0016853	isomerase activity	3.31028E-4	0.00281655	8.37079E-6	35	514	1165	40821	over
GO:0006950	response to stress	3.31028E-4	0.0028923	8.71913E-6	115	2594	1085	38741	over
GO:0046906	tetrapyrrole binding	8.01963E-4	0.00719169	1.97815E-5	25	320	1175	41015	over
GO:0030151	molybdenum ion binding	0.00127042	0.0116826	2.86919E-5	5	9	1195	41326	over
GO:0042221	response to chemical stimulus	0.00127251	0.0121527	3.08492E-5	127	3020	1073	38315	over
GO:0004197	cysteine-type endopeptidase activity	0.00127251	0.0125076	3.24081E-5	18	192	1182	41143	over
GO:0051789	response to protein stimulus	0.00127251	0.0129586	3.37167E-5	21	250	1179	41085	over
GO:0006986	response to unfolded protein	0.00127251	0.0129586	3.37167E-5	21	250	1179	41085	over
GO:0016869	intramolecular transferase activity, transferring amino groups	0.00143906	0.0155543	4.02953E-5	4	4	1196	41331	over
GO:0042286	glutamate-1-semialdehyde 2,1-aminomutase activity	0.00143906	0.0155543	4.02953E-5	4	4	1196	41331	over
GO:0009617	response to bacterium	0.00143906	0.0157051	4.16768E-5	24	315	1176	41020	over
GO:0015431	glutathione S-conjugate-exporting ATPase activity	0.00148974	0.0166199	4.20363E-5	5	10	1195	41325	over
GO:0005372	water transporter activity	0.00163599	0.0190394	5.10069E-5	9	52	1191	41283	over
GO:0015250	water channel activity	0.00163599	0.0190394	5.10069E-5	9	52	1191	41283	over
GO:0009119	ribonucleoside metabolic process	0.00166275	0.0197553	5.43078E-5	10	66	1190	41269	over
GO:0006457	protein folding	0.00168604	0.0204423	5.62086E-5	43	759	1157	40576	over

GO:0009628	response to abiotic stimulus	0.00187089	0.0231149	6.55646E-5	97	2211	1103	39124	over
GO:0004871	signal transducer activity	0.00232542	0.0297783	8.21618E-5	42	748	1158	40587	over
GO:0060089	molecular transducer activity	0.00232542	0.0297783	8.21618E-5	42	748	1158	40587	over
GO:0042631	cellular response to water deprivation	0.00234119	0.0305448	8.26397E-5	5	12	1195	41323	over
GO:0008629	induction of apoptosis by intracellular signals	0.0026566	0.0365095	8.77088E-5	3	1	1197	41334	over
GO:0008631	induction of apoptosis by oxidative stress	0.0026566	0.0365095	8.77088E-5	3	1	1197	41334	over
GO:0010327	acetyl CoA:(Z)-3-hexen-1-ol acetyltransferase activity	0.0026566	0.0365095	8.77088E-5	3	1	1197	41334	over
GO:0022803	passive transmembrane transporter activity	0.00273496	0.0388812	9.56505E-5	20	250	1180	41085	over
GO:0015267	channel activity	0.00273496	0.0388812	9.56505E-5	20	250	1180	41085	over
GO:0005262	calcium channel activity	0.00308329	0.0444603	1.11767E-4	5	13	1195	41322	over
GO:0004672	protein kinase activity	0.0042728	0.062082	1.6638E-4	92	2129	1108	39206	over
GO:0019953	sexual reproduction	0.00585108	0.0866143	2.15102E-4	7	37	1193	41298	over
GO:0006767	water-soluble vitamin metabolic process	0.00585108	0.0873769	2.16567E-4	20	267	1180	41068	over
GO:0030528	transcription regulator activity	0.00585108	0.0880372	2.2123E-4	75	1668	1125	39667	over
GO:0008271	secondary active sulfate transmembrane transporter activity	0.00691065	0.105614	2.60282E-4	4	8	1196	41327	over
GO:0048046	apoplast	0.00691065	0.106549	2.68122E-4	13	133	1187	41202	over

GO:0042277	peptide binding	0.00691065	0.107767	2.7325E-4	8	52	1192	41283	over
GO:0019321	pentose metabolic process	0.00722785	0.114027	3.05033E-4	12	117	1188	41218	over
GO:0015288	porin activity	0.00744853	0.120578	3.27037E-4	13	136	1187	41199	over
GO:0022829	wide pore channel activity	0.00744853	0.120578	3.27037E-4	13	136	1187	41199	over
GO:0004872	receptor activity	0.00750874	0.123138	3.37043E-4	29	481	1171	40854	over
GO:0015116	sulfate transmembrane transporter activity	0.00823814	0.136042	3.6757E-4	4	9	1196	41326	over
GO:0035264	multicellular organism growth	0.00872126	0.145285	4.00998E-4	6	29	1194	41306	over
GO:0000234	phosphoethanolamine N-methyltransferase activity	0.0115442	0.191697	5.03121E-4	4	10	1196	41325	over
GO:0003700	transcription factor activity	0.0115442	0.193831	5.14618E-4	58	1247	1142	40088	over
GO:0016859	cis-trans isomerase activity	0.0115442	0.196958	5.4054E-4	13	144	1187	41191	over
GO:0003755	peptidyl-prolyl cis-trans isomerase activity	0.0115442	0.196958	5.4054E-4	13	144	1187	41191	over
GO:0022838	substrate specific channel activity	0.0118962	0.204681	5.69949E-4	15	184	1185	41151	over
GO:0006766	vitamin metabolic process	0.0165762	0.276211	7.67856E-4	20	297	1180	41038	over
GO:0005955	calcineurin complex	0.0189396	0.312481	7.95276E-4	2	0	1198	41335	over
GO:0009612	response to mechanical stimulus	0.0189396	0.318564	8.41232E-4	6	34	1194	41301	over
GO:0009638	phototropism	0.0189396	0.318564	8.41232E-4	6	34	1194	41301	over

GO:0031410	cytoplasmic vesicle	0.0191309	0.327973	8.59983E-4	182	4977	1018	36358	over
GO:0016023	cytoplasmic membrane-bound vesicle	0.0191309	0.327973	8.59983E-4	182	4977	1018	36358	over
GO:0009931	calcium-dependent protein serine/threonine kinase activity	0.0191309	0.342123	8.74477E-4	4	12	1196	41323	over
GO:0008272	sulfate transport	0.0191309	0.342123	8.74477E-4	4	12	1196	41323	over
GO:0031982	vesicle	0.0191309	0.342469	8.75475E-4	182	4979	1018	36356	over
GO:0031988	membrane-bound vesicle	0.0191309	0.342469	8.75475E-4	182	4979	1018	36356	over
GO:0008234	cysteine-type peptidase activity	0.0191309	0.343552	8.95414E-4	20	301	1180	41034	over
GO:0009116	nucleoside metabolic process	0.0197882	0.35617	9.86749E-4	12	135	1188	41200	over
GO:0047918	GDP-mannose 3,5-epimerase activity	0.0226821	0.403135	0.00112783	3	5	1197	41330	over
GO:0005245	voltage-gated calcium channel activity	0.0226821	0.403135	0.00112783	3	5	1197	41330	over
GO:0005509	calcium ion binding	0.0272368	0.465549	0.00145211	36	710	1164	40625	over
GO:0016157	sucrose synthase activity	0.0355252	0.562239	0.0019912	6	41	1194	41294	over
GO:0044421	extracellular region part	0.0385839	0.596214	0.00218089	5	28	1195	41307	over
GO:0004751	ribose-5-phosphate isomerase activity	0.0417893	0.629416	0.00231662	3	7	1197	41328	over
GO:0003785	actin monomer binding	0.0464023	0.671714	0.00234103	2	1	1198	41334	over

GOSSIP
Test-Set: tm1.yuc.25.down.txt
Tests for all terms in Gene Ontology whether it is enriched in a test group when compared to a reference group using Fisher's excact test with Multiple Testing.
Pub: Biological Profiling of Gene Groups utilizing Gene Ontology A Statistical Framework
Poster: GOSSIP: Biological Profiling of Gene Groups utilizing Gene Ontology
by Nils Blthgen, Karsten Brand, Hanspeter Herzel, Dieter Beule

GO Term	Name	FDR	FWER	single test p-Value	# in test group	# in reference group	# non annot test	# non annot reference group	Over/Under
GO:0003824	catalytic activity	1.08483E-8	4.33932E-8	0.0	513	17996	427	23599	over
GO:0006725	aromatic compound metabolic process	1.08483E-8	4.33932E-8	0.0	58	685	882	40910	over
GO:0009699	phenylpropanoid biosynthetic process	1.08483E-8	4.33932E-8	0.0	43	275	897	41320	over
GO:0045548	phenylalanine ammonia-lyase activity	1.08483E-8	4.33932E-8	0.0	11	18	929	41577	over
GO:0016211	ammonia ligase activity	1.08483E-8	4.33932E-8	0.0	14	44	926	41551	over
GO:0019438	aromatic compound biosynthetic process	1.08483E-8	4.33932E-8	0.0	44	371	896	41224	over
GO:0009698	phenylpropanoid metabolic process	1.08483E-8	4.33932E-8	0.0	55	345	885	41250	over
GO:0008415	acyltransferase activity	1.08483E-8	4.33932E-8	0.0	50	536	890	41059	over
GO:0006575	amino acid derivative metabolic process	1.08483E-8	4.33932E-8	0.0	58	578	882	41017	over
GO:0009813	flavonoid biosynthetic process	1.08483E-8	4.33932E-8	0.0	39	136	901	41459	over

GO:0016747	transferase activity, transferring groups other than amino-acyl groups	1.08483E-8	4.33932E-8	0.0	50	546	890	41049	over
GO:0016880	acid-ammonia (or amide) ligase activity	1.08483E-8	4.33932E-8	0.0	14	44	926	41551	over
GO:0016746	transferase activity, transferring acyl groups	1.08483E-8	4.33932E-8	0.0	51	608	889	40987	over
GO:0042398	amino acid derivative biosynthetic process	1.08483E-8	4.33932E-8	0.0	45	457	895	41138	over
GO:0019748	secondary metabolic process	1.08483E-8	4.33932E-8	0.0	71	778	869	40817	over
GO:0009812	flavonoid metabolic process	1.08483E-8	4.33932E-8	0.0	40	151	900	41444	over
GO:0016210	naringenin-chalcone synthase activity	1.15343E-8	4.90209E-8	1.50888E-11	16	14	924	41581	over
GO:0006519	amino acid and derivative metabolic process	1.31559E-8	6.14863E-8	7.98185E-11	80	1603	860	39992	over
GO:0006559	L-phenylalanine catabolic process	1.31559E-8	6.24906E-8	9.18372E-11	11	23	929	41572	over
GO:0031418	L-ascorbic acid binding	1.41666E-8	7.0833E-8	1.08828E-10	13	40	927	41555	over
GO:0006631	fatty acid metabolic process	2.33932E-8	1.22814E-7	2.84534E-10	40	540	900	41055	over
GO:0009074	aromatic amino acid family catabolic process	2.49119E-8	1.37015E-7	3.49522E-10	11	27	929	41568	over
GO:0016841	ammonia-lyase activity	3.18965E-8	1.83405E-7	5.07378E-10	11	28	929	41567	over

GO:0045486	naringenin 3-dioxygenase activity	6.21899E-8	3.73139E-7	1.17065E-9	9	15	931	41580	over
GO:0006558	L-phenylalanine metabolic process	8.72859E-8	5.45537E-7	1.76646E-9	12	42	928	41553	over
GO:0019439	aromatic compound catabolic process	1.03568E-7	6.73193E-7	2.21236E-9	11	33	929	41562	over
GO:0045552	dihydrokaempferol 4-reductase activity	1.55516E-6	1.04973E-5	3.07052E-8	5	1	935	41594	over
GO:0032787	monocarboxylic acid metabolic process	3.03785E-6	2.12647E-5	7.04721E-8	45	797	895	40798	over
GO:0042335	cuticle development	4.13189E-6	2.99557E-5	9.97999E-8	10	39	930	41556	over
GO:0000038	very-long-chain fatty acid metabolic process	5.37591E-6	4.03185E-5	1.22211E-7	10	40	930	41555	over
GO:0016840	carbon-nitrogen lyase activity	5.90011E-6	4.57248E-5	1.48004E-7	11	53	929	41542	over
GO:0005506	iron ion binding	7.1709E-6	5.73656E-5	1.7667E-7	43	769	897	40826	over
GO:0009922	fatty acid elongase activity	1.23861E-5	1.0218E-4	2.76319E-7	5	3	935	41592	over
GO:0016705	oxidoreductase activity, acting on paired donors, with incorporation or reduction of molecular oxygen	1.41136E-5	1.23062E-4	3.42539E-7	25	324	915	41271	over
GO:0016706	oxidoreductase activity, acting on paired donors, with incorporation or reduction of molecular oxygen, 2-oxoglutarate as one donor, and incorporation of one atom each of oxygen into both donors	1.41136E-5	1.23486E-4	3.43864E-7	15	120	925	41475	over
GO:0006633	fatty acid biosynthetic process	1.51426E-5	1.36274E-4	4.20328E-7	28	399	912	41196	over
GO:0046072	dTDP metabolic process	3.32075E-5	4.06709E-4	1.1643E-6	4	1	936	41594	over
GO:0004798	thymidylate kinase activity	3.32075E-5	4.06709E-4	1.1643E-6	4	1	936	41594	over

GO:0009148	pyrimidine nucleoside triphosphate biosynthetic process	3.32075E-5	4.06709E-4	1.1643E-6	4	1	936	41594	over
GO:0009138	pyrimidine nucleoside diphosphate metabolic process	3.32075E-5	4.06709E-4	1.1643E-6	4	1	936	41594	over
GO:0009197	pyrimidine deoxyribonucleoside diphosphate biosynthetic process	3.32075E-5	4.06709E-4	1.1643E-6	4	1	936	41594	over
GO:0009133	nucleoside diphosphate biosynthetic process	3.32075E-5	4.06709E-4	1.1643E-6	4	1	936	41594	over
GO:0046075	dTTP metabolic process	3.32075E-5	4.06709E-4	1.1643E-6	4	1	936	41594	over
GO:0009139	pyrimidine nucleoside diphosphate biosynthetic process	3.32075E-5	4.06709E-4	1.1643E-6	4	1	936	41594	over
GO:0009189	deoxyribonucleoside diphosphate biosynthetic process	3.32075E-5	4.06709E-4	1.1643E-6	4	1	936	41594	over
GO:0006233	dTDP biosynthetic process	3.32075E-5	4.06709E-4	1.1643E-6	4	1	936	41594	over
GO:0009196	pyrimidine deoxyribonucleoside diphosphate metabolic process	3.32075E-5	4.06709E-4	1.1643E-6	4	1	936	41594	over
GO:0006235	dTTP biosynthetic process	3.32075E-5	4.06709E-4	1.1643E-6	4	1	936	41594	over
GO:0009212	pyrimidine deoxyribonucleoside triphosphate biosynthetic process	3.32075E-5	4.06709E-4	1.1643E-6	4	1	936	41594	over
GO:0016740	transferase activity	3.64106E-5	4.64211E-4	1.28434E-6	197	6278	743	35317	over
GO:0004312	fatty-acid synthase activity	3.64106E-5	4.70328E-4	1.33198E-6	11	68	929	41527	over
GO:0046394	carboxylic acid biosynthetic process	3.64106E-5	4.82324E-4	1.34999E-6	28	425	912	41170	over
GO:0016053	organic acid biosynthetic process	3.64106E-5	4.82324E-4	1.34999E-6	28	425	912	41170	over
GO:0019752	carboxylic acid metabolic process	5.42489E-5	7.37073E-4	2.17241E-6	81	2062	859	39533	over

GO:0006082	organic acid metabolic process	5.42489E-5	7.45644E-4	2.21099E-6	81	2063	859	39532	over
GO:0009202	deoxyribonucleoside triphosphate biosynthetic process	9.2117E-5	0.00128881	3.4314E-6	4	2	936	41593	over
GO:0006629	lipid metabolic process	1.09068E-4	0.00155301	4.18879E-6	59	1370	881	40225	over
GO:0044255	cellular lipid metabolic process	1.29543E-4	0.00187661	5.53028E-6	53	1192	887	40403	over
GO:0016711	flavonoid 3'-monooxygenase activity	2.77025E-4	0.00407779	1.07593E-5	3	0	937	41595	over
GO:0016491	oxidoreductase activity	2.82882E-4	0.00423425	1.10171E-5	125	3741	815	37854	over
GO:0005576	extracellular region	3.42206E-4	0.00520505	1.39033E-5	24	379	916	41216	over
GO:0051213	dioxygenase activity	3.85582E-4	0.00605453	1.54555E-5	4	4	936	41591	over
GO:0050589	leucocyanidin oxygenase activity	3.85582E-4	0.00605453	1.54555E-5	4	4	936	41591	over
GO:0007267	cell-cell signaling	4.03525E-4	0.00643561	1.74768E-5	7	31	933	41564	over
GO:0019842	vitamin binding	4.60075E-4	0.00744836	2.03386E-5	21	312	919	41283	over
GO:0009186	deoxyribonucleoside diphosphate metabolic process	5.95213E-4	0.0102149	2.73317E-5	4	5	936	41590	over
GO:0009211	pyrimidine deoxyribonucleoside triphosphate metabolic process	5.95213E-4	0.0102149	2.73317E-5	4	5	936	41590	over
GO:0009120	deoxyribonucleoside metabolic process	5.95213E-4	0.0102149	2.73317E-5	4	5	936	41590	over
GO:0046125	pyrimidine deoxyribonucleoside metabolic process	5.95213E-4	0.0102149	2.73317E-5	4	5	936	41590	over
GO:0009072	aromatic amino acid family metabolic process	6.62288E-4	0.0115232	3.36101E-5	12	118	928	41477	over

GO:0010025	wax biosynthetic process	7.49691E-4	0.0134038	3.98382E-5	8	50	932	41545	over
GO:0010166	wax metabolic process	7.49691E-4	0.0134038	3.98382E-5	8	50	932	41545	over
GO:0016165	lipoxygenase activity	9.31285E-4	0.0168524	4.32455E-5	6	24	934	41571	over
GO:0016724	oxidoreductase activity, oxidizing metal ions, oxygen as acceptor	9.4406E-4	0.0182408	4.47538E-5	4	6	936	41589	over
GO:0009200	deoxyribonucleoside triphosphate metabolic process	9.4406E-4	0.0182408	4.47538E-5	4	6	936	41589	over
GO:0009132	nucleoside diphosphate metabolic process	9.4406E-4	0.0182408	4.47538E-5	4	6	936	41589	over
GO:0004322	ferroxidase activity	9.4406E-4	0.0182408	4.47538E-5	4	6	936	41589	over
GO:0010039	response to iron ion	9.4406E-4	0.0182408	4.47538E-5	4	6	936	41589	over
GO:0016297	acyl-[acyl-carrier-protein] hydrolase activity	0.00115576	0.0228501	6.13502E-5	5	15	935	41580	over
GO:0010281	acyl-ACP thioesterase activity	0.00115576	0.0228501	6.13502E-5	5	15	935	41580	over
GO:0003777	microtubule motor activity	0.00118924	0.0237945	6.47005E-5	11	107	929	41488	over
GO:0005875	microtubule associated complex	0.00231906	0.0464288	1.24715E-4	10	96	930	41499	over
GO:0003774	motor activity	0.00243379	0.0492476	1.34001E-4	15	206	925	41389	over
GO:0008199	ferric iron binding	0.00265719	0.0542732	1.44509E-4	4	9	936	41586	over
GO:0004489	methylenetetrahydrofolate reductase (NADPH) activity	0.00343767	0.070447	1.98774E-4	4	10	936	41585	over
GO:0006826	iron ion transport	0.00492041	0.100387	2.66319E-4	4	11	936	41584	over

GO:0000910	cytokinesis	0.00561014	0.114872	3.29407E-4	9	89	931	41506	over
GO:0000036	acyl carrier activity	0.00583579	0.12049	3.47864E-4	6	37	934	41558	over
GO:0009221	pyrimidine deoxyribonucleotide biosynthetic process	0.00777069	0.158781	4.48281E-4	4	13	936	41582	over
GO:0009263	deoxyribonucleotide biosynthetic process	0.0112171	0.223062	5.66313E-4	4	14	936	41581	over
GO:0004675	transmembrane receptor protein serine/threonine kinase activity	0.0127714	0.252159	7.04832E-4	4	15	936	41580	over
GO:0007017	microtubule-based process	0.0133608	0.264582	7.64141E-4	19	355	921	41240	over
GO:0007018	microtubule-based movement	0.0134012	0.267722	7.91363E-4	16	273	924	41322	over
GO:0009219	pyrimidine deoxyribonucleotide metabolic process	0.0177158	0.343467	0.00105079	4	17	936	41578	over
GO:0047458	beta-pyrazolylalanine synthase activity	0.0177158	0.343467	0.00105079	4	17	936	41578	over
GO:0015630	microtubule cytoskeleton	0.0182757	0.355096	0.00112541	22	456	918	41139	over
GO:0016722	oxidoreductase activity, oxidizing metal ions	0.0205939	0.399352	0.00126198	4	18	936	41577	over
GO:0008395	steroid hydroxylase activity	0.0205939	0.399352	0.00126198	4	18	936	41577	over
GO:0009147	pyrimidine nucleoside triphosphate metabolic process	0.0205939	0.399352	0.00126198	4	18	936	41577	over
GO:0045430	chalcone isomerase activity	0.0252543	0.468169	0.00144211	2	1	938	41594	over
GO:0030705	cytoskeleton-dependent intracellular transport	0.0287526	0.516206	0.00169226	16	295	924	41300	over
GO:0016879	ligase activity, forming carbon-nitrogen bonds	0.0308499	0.544693	0.00185996	31	763	909	40832	over

GO:0004320	oleoyl-[acyl-carrier-protein] hydrolase activity	0.034108	0.584561	0.00203883	3	9	937	41586	over
GO:0009262	deoxyribonucleotide metabolic process	0.0377385	0.625205	0.00240481	4	22	936	41573	over
GO:0033559	unsaturated fatty acid metabolic process	0.0480282	0.733153	0.0028419	2	2	938	41593	over
GO:0042389	omega-3 fatty acid desaturase activity	0.0480282	0.733153	0.0028419	2	2	938	41593	over
GO:0045604	regulation of epidermal cell differentiation	0.0480282	0.733153	0.0028419	2	2	938	41593	over
GO:0045682	regulation of epidermis development	0.0480282	0.733153	0.0028419	2	2	938	41593	over
GO:0010321	regulation of vegetative phase change	0.0480282	0.733153	0.0028419	2	2	938	41593	over
GO:0006636	unsaturated fatty acid biosynthetic process	0.0480282	0.733153	0.0028419	2	2	938	41593	over

GOSSIP
Test-Set: yuc.2.7.up.txt
Tests for all terms in Gene Ontology whether it is enriched in a test group when compared to a reference group using Fisher's excact test with Multiple Testing.
Pub: Biological Profiling of Gene Groups utilizing Gene Ontology A Statistical Framework
Poster: GOSSIP: Biological Profiling of Gene Groups utilizing Gene Ontology
by Nils Blthgen, Karsten Brand, Hanspeter Herzel, Dieter Beule

GO Term	Name	FDR	FWER	single test p-Value	# in test group	# in reference group	# non annot test	# non annot reference group	Over/Under
GO:0033279	ribosomal subunit	1.73428E-8	2.33995E-8	0.0	45	580	703	41207	over
GO:0006412	translation	1.73428E-8	2.33995E-8	0.0	95	2147	653	39640	over

GO:0030529	ribonucleoprotein complex	1.73428E-8	2.34908E-8	1.24024E-12	87	1679	661	40108	over
GO:0005198	structural molecule activity	1.73428E-8	2.5835E-8	1.48471E-11	85	1780	663	40007	over
GO:0003735	structural constituent of ribosome	1.73428E-8	3.01385E-8	1.87462E-11	78	1417	670	40370	over
GO:0005840	ribosome	1.73428E-8	3.05994E-8	2.16442E-11	80	1517	668	40270	over
GO:0044249	cellular biosynthetic process	1.73428E-8	3.23797E-8	3.4158E-11	142	4538	606	37249	over
GO:0015934	large ribosomal subunit	1.73428E-8	3.46857E-8	4.54068E-11	28	260	720	41527	over
GO:0022613	ribonucleoprotein complex biogenesis and assembly	2.7162E-8	6.81779E-8	1.61368E-10	44	796	704	40991	over
GO:0043228	non-membrane-bound organelle	2.7162E-8	7.46954E-8	1.89029E-10	98	2738	650	39049	over
GO:0043232	intracellular non-membrane-bound organelle	2.7162E-8	7.46954E-8	1.89029E-10	98	2738	650	39049	over
GO:0042254	ribosome biogenesis and assembly	3.30583E-8	9.91748E-8	2.96442E-10	36	569	712	41218	over
GO:0009059	macromolecule biosynthetic process	7.58534E-8	2.46524E-7	7.2012E-10	105	3117	643	38670	over
GO:0000080	G1 phase of mitotic cell cycle	7.32023E-6	2.74505E-5	8.89563E-8	5	3	743	41784	over
GO:0051318	G1 phase	7.32023E-6	2.74505E-5	8.89563E-8	5	3	743	41784	over
GO:0003723	RNA binding	9.36186E-6	3.74468E-5	1.11354E-7	53	1321	695	40466	over
GO:0003676	nucleic acid binding	2.24694E-5	9.54904E-5	3.18534E-7	158	5961	590	35826	over
GO:0009058	biosynthetic process	7.48482E-5	3.3676E-4	1.13488E-6	154	5899	594	35888	over

GO:0044445	cytosolic part	2.78333E-4	0.00132121	4.01906E-6	21	354	727	41433	over
GO:0022402	cell cycle process	3.99281E-4	0.00199442	5.44706E-6	19	303	729	41484	over
GO:0051329	interphase of mitotic cell cycle	7.58484E-4	0.00418104	1.17584E-5	5	13	743	41774	over
GO:0051325	interphase	7.58484E-4	0.00418104	1.17584E-5	5	13	743	41774	over
GO:0019207	kinase regulator activity	7.58484E-4	0.00454057	1.28974E-5	8	54	740	41733	over
GO:0019887	protein kinase regulator activity	7.58484E-4	0.00454057	1.28974E-5	8	54	740	41733	over
GO:0000074	regulation of progression through cell cycle	0.00136952	0.00852297	2.24754E-5	12	144	736	41643	over
GO:0045036	protein targeting to chloroplast	0.00142402	0.00926662	2.562E-5	6	28	742	41759	over
GO:0006396	RNA processing	0.00142402	0.0095661	2.65772E-5	25	536	723	41251	over
GO:0051726	regulation of cell cycle	0.0014327	0.00997883	2.71594E-5	12	147	736	41640	over
GO:0009281	cytosolic ribosome (sensu Bacteria)	0.00229812	0.0170883	4.95415E-5	6	32	742	41755	over
GO:0009282	cytosolic large ribosomal subunit (sensu Bacteria)	0.00229812	0.0170883	4.95415E-5	6	32	742	41755	over
GO:0006469	negative regulation of protein kinase activity	0.00234678	0.01975	5.27559E-5	3	2	745	41785	over
GO:0051348	negative regulation of transferase activity	0.00234678	0.01975	5.27559E-5	3	2	745	41785	over
GO:0000079	regulation of cyclin-dependent protein kinase activity	0.00234678	0.01975	5.27559E-5	3	2	745	41785	over
GO:0045736	negative regulation of cyclin-dependent protein kinase activity	0.00234678	0.01975	5.27559E-5	3	2	745	41785	over

GO:0005830	cytosolic ribosome (sensu Eukaryota)	0.0029964	0.0258779	7.26868E-5	15	247	733	41540	over
GO:0016538	cyclin-dependent protein kinase regulator activity	0.00340318	0.0308535	8.86877E-5	6	36	742	41751	over
GO:0022403	cell cycle phase	0.00340318	0.0309893	9.02598E-5	11	142	737	41645	over
GO:0045038	protein import into chloroplast thylakoid membrane	0.00413245	0.0392412	1.04128E-4	3	3	745	41784	over
GO:0007049	cell cycle	0.00413245	0.0394908	1.05512E-4	20	414	728	41373	over
GO:0015935	small ribosomal subunit	0.00450377	0.0446093	1.17301E-4	17	320	731	41467	over
GO:0005843	cytosolic small ribosomal subunit (sensu Eukaryota)	0.00450377	0.0458974	1.22286E-4	14	230	734	41557	over
GO:0043229	intracellular organelle	0.00450377	0.0468345	1.28583E-4	534	27156	214	14631	over
GO:0043226	organelle	0.00450377	0.0472626	1.29933E-4	534	27158	214	14629	over
GO:0005730	nucleolus	0.0047954	0.0513828	1.44944E-4	14	234	734	41553	over
GO:0009554	megasporogenesis	0.00617342	0.067095	1.79836E-4	3	4	745	41783	over
GO:0048443	stamen development	0.00816899	0.0915246	2.76674E-4	7	65	741	41722	over
GO:0048466	androecium development	0.00816899	0.0915246	2.76674E-4	7	65	741	41722	over
GO:0018117	protein amino acid adenylylation	0.00854213	0.0993542	2.83971E-4	3	5	745	41782	over
GO:0018175	protein amino acid nucleotidylation	0.00854213	0.0993542	2.83971E-4	3	5	745	41782	over
GO:0032991	macromolecular complex	0.01045	0.122456	3.10951E-4	126	5196	622	36591	over

GO:0005622	intracellular	0.0106015	0.126435	3.33132E-4	557	28697	191	13090	over
GO:0016071	mRNA metabolic process	0.0111097	0.134484	3.65336E-4	15	289	733	41498	over
GO:0043231	intracellular membrane-bound organelle	0.0114821	0.14313	3.87355E-4	513	26151	235	15636	over
GO:0043227	membrane-bound organelle	0.0114821	0.143596	3.92969E-4	513	26154	235	15633	over
GO:0006397	mRNA processing	0.0116575	0.148108	4.15629E-4	14	261	734	41526	over
GO:0045786	negative regulation of progression through cell cycle	0.0121794	0.158165	4.20386E-4	3	6	745	41781	over
GO:0044424	intracellular part	0.0121794	0.159335	4.27506E-4	550	28334	198	13453	over
GO:0031981	nuclear lumen	0.0126304	0.167354	4.49527E-4	19	430	729	41357	over
GO:0015380	anion exchanger activity	0.0136626	0.188088	5.39242E-4	4	18	744	41769	over
GO:0015108	chloride transmembrane transporter activity	0.0136626	0.188088	5.39242E-4	4	18	744	41769	over
GO:0015106	bicarbonate transmembrane transporter activity	0.0136626	0.188088	5.39242E-4	4	18	744	41769	over
GO:0016246	RNA interference	0.0164917	0.225574	6.56089E-4	5	35	743	41752	over
GO:0048653	anther development	0.0181561	0.248719	7.72286E-4	6	56	742	41731	over
GO:0030332	cyclin binding	0.0191696	0.264153	8.04326E-4	3	8	745	41779	over
GO:0000278	mitotic cell cycle	0.0198317	0.275508	8.75254E-4	7	80	741	41707	over
GO:0004861	cyclin-dependent protein kinase inhibitor activity	0.0225971	0.31899	9.15699E-4	2	1	746	41786	over

GO:0007050	cell cycle arrest	0.0225971	0.31899	9.15699E-4	2	1	746	41786	over
GO:0051782	negative regulation of cell division	0.0225971	0.31899	9.15699E-4	2	1	746	41786	over
GO:0031050	dsRNA fragmentation	0.0232768	0.334605	0.00102209	5	39	743	41748	over
GO:0043331	response to dsRNA	0.0232768	0.334605	0.00102209	5	39	743	41748	over
GO:0010050	vegetative phase change	0.0236327	0.342634	0.00104234	4	22	744	41765	over
GO:0010234	tapetal cell fate specification	0.0242803	0.361877	0.00105844	3	9	745	41778	over
GO:0016701	oxidoreductase activity, acting on single donors with incorporation of molecular oxygen	0.0242803	0.361877	0.00105844	3	9	745	41778	over
GO:0050113	inositol oxygenase activity	0.0242803	0.361877	0.00105844	3	9	745	41778	over
GO:0019843	rRNA binding	0.0253203	0.377989	0.00118522	11	196	737	41591	over
GO:0010267	RNA interference, production of ta-siRNAs	0.0258262	0.387829	0.00120672	4	23	744	41764	over
GO:0005737	cytoplasm	0.0258295	0.399707	0.00129113	490	25088	258	16699	over
GO:0009536	plastid	0.0258295	0.402019	0.00132182	244	11500	504	30287	over
GO:0006414	translational elongation	0.0258295	0.405447	0.00135741	12	231	736	41556	over
GO:0048654	anther morphogenesis	0.0258295	0.422434	0.00135805	3	10	745	41777	over
GO:0048656	tapetal layer formation	0.0258295	0.422434	0.00135805	3	10	745	41777	over
GO:0048655	tapetal layer morphogenesis	0.0258295	0.422434	0.00135805	3	10	745	41777	over

GO:0048455	stamen formation	0.0258295	0.422434	0.00135805	3	10	745	41777	over
GO:0048657	tapetal cell differentiation	0.0258295	0.422434	0.00135805	3	10	745	41777	over
GO:0048658	tapetal layer development	0.0258295	0.422434	0.00135805	3	10	745	41777	over
GO:0015301	anion:anion antiporter activity	0.026399	0.433137	0.00138841	4	24	744	41763	over
GO:0006526	arginine biosynthetic process	0.0282844	0.461372	0.00158833	4	25	744	41762	over
GO:0045941	positive regulation of transcription	0.0282844	0.470846	0.00166992	5	44	743	41743	over
GO:0045935	positive regulation of nucleobase, nucleoside, nucleotide and nucleic acid metabolic process	0.0282844	0.470846	0.00166992	5	44	743	41743	over
GO:0051607	defense response to virus	0.0282844	0.470846	0.00166992	5	44	743	41743	over
GO:0004860	protein kinase inhibitor activity	0.0350917	0.554782	0.00181001	2	2	746	41785	over
GO:0019210	kinase inhibitor activity	0.0350917	0.554782	0.00181001	2	2	746	41785	over
GO:0031325	positive regulation of cellular metabolic process	0.0350917	0.561667	0.00182897	5	45	743	41742	over
GO:0009893	positive regulation of metabolic process	0.0350917	0.561667	0.00182897	5	45	743	41742	over
GO:0040029	regulation of gene expression, epigenetic	0.0360358	0.575134	0.00199086	11	210	737	41577	over
GO:0009163	nucleoside biosynthetic process	0.036514	0.605944	0.00210465	3	12	745	41775	over
GO:0046132	pyrimidine ribonucleoside biosynthetic process	0.036514	0.605944	0.00210465	3	12	745	41775	over
GO:0009052	pentose-phosphate shunt, non-oxidative branch	0.036514	0.605944	0.00210465	3	12	745	41775	over

GO:0046109	uridine biosynthetic process	0.036514	0.605944	0.00210465	3	12	745	41775	over
GO:0042455	ribonucleoside biosynthetic process	0.036514	0.605944	0.00210465	3	12	745	41775	over
GO:0046108	uridine metabolic process	0.036514	0.605944	0.00210465	3	12	745	41775	over
GO:0046134	pyrimidine nucleoside biosynthetic process	0.036514	0.605944	0.00210465	3	12	745	41775	over
GO:0008380	RNA splicing	0.0373982	0.618319	0.00225851	10	182	738	41605	over
GO:0006221	pyrimidine nucleotide biosynthetic process	0.039431	0.641346	0.00254831	6	72	742	41715	over
GO:0048448	stamen morphogenesis	0.0403373	0.656698	0.00255667	3	13	745	41774	over
GO:0009864	induced systemic resistance, jasmonic acid mediated signaling pathway	0.0403373	0.656698	0.00255667	3	13	745	41774	over
GO:0000377	RNA splicing, via transesterification reactions with bulged adenosine as nucleophile	0.0404046	0.667543	0.00260203	7	98	741	41689	over
GO:0000398	nuclear mRNA splicing, via spliceosome	0.0404046	0.667543	0.00260203	7	98	741	41689	over
GO:0000375	RNA splicing, via transesterification reactions	0.0404046	0.667543	0.00260203	7	98	741	41689	over
GO:0004180	carboxypeptidase activity	0.0417244	0.684606	0.00289314	7	100	741	41687	over
GO:0009556	microsporogenesis	0.0417244	0.689177	0.00289365	4	30	744	41757	over
GO:0046112	nucleobase biosynthetic process	0.0417244	0.689177	0.00289365	4	30	744	41757	over
GO:0046653	tetrahydrofolate metabolic process	0.0458133	0.729093	0.00298152	2	3	746	41784	over

GO:0046654	tetrahydrofolate biosynthetic process	0.0458133	0.729093	0.00298152	2	3	746	41784	over
GO:0030422	RNA interference, production of siRNA	0.0476093	0.750078	0.00322213	4	31	744	41756	over
GO:0006220	pyrimidine nucleotide metabolic process	0.0476093	0.75349	0.00327571	6	76	742	41711	over
GO:0005634	nucleus	0.0476093	0.761129	0.00347445	93	3908	655	37879	over
GO:0009089	lysine biosynthetic process via diaminopimelate	0.0476093	0.766006	0.00352629	5	53	743	41734	over
GO:0010025	wax biosynthetic process	0.0476093	0.766006	0.00352629	5	53	743	41734	over
GO:0046451	diaminopimelate metabolic process	0.0476093	0.766006	0.00352629	5	53	743	41734	over
GO:0009085	lysine biosynthetic process	0.0476093	0.766006	0.00352629	5	53	743	41734	over
GO:0010166	wax metabolic process	0.0476093	0.766006	0.00352629	5	53	743	41734	over
GO:0048437	floral organ development	0.0476775	0.771996	0.00361185	10	195	738	41592	over
GO:0048569	post-embryonic organ development	0.0476775	0.771996	0.00361185	10	195	738	41592	over
GO:0048438	floral whorl development	0.0492553	0.785544	0.00370548	9	164	739	41623	over
GO:0044444	cytoplasmic part	0.0498617	0.792181	0.00385118	473	24382	275	17405	over

GOSSIP
Test-Set: yuc.2.7.down.txt
Tests for all terms in Gene Ontology whether it is enriched in a test group when compared to a reference group using Fisher's excact test with Multiple Testing.
Pub: Biological Profiling of Gene Groups utilizing Gene Ontology A Statistical Framework
Poster: GOSSIP: Biological Profiling of Gene Groups utilizing Gene Ontology

by Nils Blthgen, Karsten Brand, Hanspeter Herzel, Dieter Beule									
**GO Term**	**Name**	**FDR**	**FWER**	**single test p-Value**	**# in test group**	**# in reference group**	**# non annot test**	**# non annot reference group**	**Over/Under**
GO:0007018	microtubule-based movement	9.72019E-9	3.46687E-8	0.0	31	258	1056	41190	over
GO:0015288	porin activity	9.72019E-9	3.46687E-8	0.0	23	126	1064	41322	over
GO:0006810	transport	9.72019E-9	3.46687E-8	0.0	257	5948	830	35500	over
GO:0051258	protein polymerization	9.72019E-9	3.46687E-8	0.0	27	199	1060	41249	over
GO:0016020	membrane	9.72019E-9	3.46687E-8	0.0	395	9697	692	31751	over
GO:0031410	cytoplasmic vesicle	9.72019E-9	3.46687E-8	0.0	207	4952	880	36496	over
GO:0031988	membrane-bound vesicle	9.72019E-9	3.46687E-8	0.0	208	4953	879	36495	over
GO:0051641	cellular localization	9.72019E-9	3.46687E-8	0.0	152	3210	935	38238	over
GO:0022829	wide pore channel activity	9.72019E-9	3.46687E-8	0.0	23	126	1064	41322	over
GO:0016023	cytoplasmic membrane-bound vesicle	9.72019E-9	3.46687E-8	0.0	207	4952	880	36496	over
GO:0051649	establishment of cellular localization	9.72019E-9	3.46687E-8	0.0	152	3203	935	38245	over
GO:0051234	establishment of localization	9.72019E-9	3.46687E-8	0.0	260	5965	827	35483	over
GO:0031982	vesicle	9.72019E-9	3.46687E-8	0.0	208	4953	879	36495	over

GO:0004351	glutamate decarboxylase activity	9.72019E-9	3.55029E-8	6.08044E-12	9	3	1078	41445	over
GO:0051179	localization	9.72019E-9	3.64507E-8	1.19956E-11	263	5986	824	35462	over
GO:0005874	microtubule	1.20584E-8	4.82337E-8	6.52008E-11	32	293	1055	41155	over
GO:0030705	cytoskeleton-dependent intracellular transport	2.05873E-8	8.74961E-8	1.9938E-10	31	280	1056	41168	over
GO:0015267	channel activity	3.22078E-8	1.52987E-7	4.04486E-10	28	242	1059	41206	over
GO:0022803	passive transmembrane transporter activity	3.22078E-8	1.52987E-7	4.04486E-10	28	242	1059	41206	over
GO:0007017	microtubule-based process	2.10572E-7	1.05286E-6	3.47399E-9	32	342	1055	41106	over
GO:0015630	microtubule cytoskeleton	7.40292E-7	3.88653E-6	1.11429E-8	36	442	1051	41006	over
GO:0019867	outer membrane	1.83433E-6	1.01304E-5	3.32912E-8	24	225	1063	41223	over
GO:0006944	membrane fusion	1.83433E-6	1.10059E-5	3.69E-8	10	29	1077	41419	over
GO:0005544	calcium-dependent phospholipid binding	1.83433E-6	1.10059E-5	3.69E-8	10	29	1077	41419	over
GO:0031224	intrinsic to membrane	5.3478E-6	3.34232E-5	1.07515E-7	93	1973	994	39475	over
GO:0006536	glutamate metabolic process	6.39197E-6	4.32264E-5	1.32777E-7	9	25	1078	41423	over
GO:0032561	guanyl ribonucleotide binding	6.39197E-6	4.52009E-5	1.44429E-7	52	874	1035	40574	over
GO:0005525	GTP binding	6.39197E-6	4.52009E-5	1.44429E-7	52	874	1035	40574	over
GO:0019001	guanyl nucleotide binding	6.39197E-6	4.63407E-5	1.54301E-7	52	876	1035	40572	over

GO:0044430	cytoskeletal part	1.14807E-5	8.61017E-5	2.58899E-7	38	552	1049	40896	over
GO:0006970	response to osmotic stress	1.21456E-5	9.41237E-5	2.9118E-7	39	578	1048	40870	over
GO:0006529	asparagine biosynthetic process	2.6476E-5	2.2502E-4	5.93358E-7	7	14	1080	41434	over
GO:0006528	asparagine metabolic process	2.6476E-5	2.2502E-4	5.93358E-7	7	14	1080	41434	over
GO:0004066	asparagine synthase (glutamine-hydrolyzing) activity	2.6476E-5	2.2502E-4	5.93358E-7	7	14	1080	41434	over
GO:0016044	membrane organization and biogenesis	3.9287E-5	3.51475E-4	1.09711E-6	14	98	1073	41350	over
GO:0016021	integral to membrane	3.9287E-5	3.5352E-4	1.12581E-6	88	1941	999	39507	over
GO:0046907	intracellular transport	4.87582E-5	4.50912E-4	1.31263E-6	71	1461	1016	39987	over
GO:0009631	cold acclimation	5.68478E-5	5.39909E-4	1.65635E-6	10	47	1077	41401	over
GO:0007010	cytoskeleton organization and biogenesis	1.6688E-4	0.00166584	5.03225E-6	38	630	1049	40818	over
GO:0003924	GTPase activity	1.6688E-4	0.00166741	5.04742E-6	27	370	1060	41078	over
GO:0005774	vacuolar membrane	1.78491E-4	0.00182786	5.44599E-6	14	114	1073	41334	over
GO:0007154	cell communication	1.8766E-4	0.0019685	6.0159E-6	90	2089	997	39359	over
GO:0009415	response to water	2.15495E-4	0.0023139	6.22507E-6	29	420	1058	41028	over
GO:0009064	glutamine family amino acid metabolic process	2.7371E-4	0.00300628	7.8515E-6	16	153	1071	41295	over
GO:0016884	carbon-nitrogen ligase activity, with glutamine as amido-N-donor	2.8726E-4	0.00322647	8.49838E-6	7	23	1080	41425	over

GO:0005856	cytoskeleton	2.92161E-4	0.00335421	8.89081E-6	38	647	1049	40801	over
GO:0022857	transmembrane transporter activity	3.01304E-4	0.00353406	9.87259E-6	78	1759	1009	39689	over
GO:0044425	membrane part	3.89893E-4	0.00466779	1.37839E-5	120	3068	967	38380	over
GO:0009414	response to water deprivation	4.4233E-4	0.00540389	1.45468E-5	27	394	1060	41054	over
GO:0005773	vacuole	4.68068E-4	0.00583378	1.65198E-5	26	374	1061	41074	over
GO:0010286	heat acclimation	5.73633E-4	0.00728715	1.78413E-5	5	9	1082	41439	over
GO:0006793	phosphorus metabolic process	5.81732E-4	0.007534	1.88776E-5	99	2432	988	39016	over
GO:0043068	positive regulation of programmed cell death	7.093E-4	0.00988108	2.61978E-5	5	10	1082	41438	over
GO:0006917	induction of apoptosis	7.093E-4	0.00988108	2.61978E-5	5	10	1082	41438	over
GO:0012502	induction of programmed cell death	7.093E-4	0.00988108	2.61978E-5	5	10	1082	41438	over
GO:0043065	positive regulation of apoptosis	7.093E-4	0.00988108	2.61978E-5	5	10	1082	41438	over
GO:0045045	secretory pathway	7.21363E-4	0.0102268	2.72449E-5	86	2056	1001	39392	over
GO:0000813	ESCRT I complex	7.61403E-4	0.0109796	2.73517E-5	4	4	1083	41444	over
GO:0005215	transporter activity	7.69025E-4	0.0112791	2.91925E-5	102	2554	985	38894	over
GO:0030170	pyridoxal phosphate binding	8.20845E-4	0.0122372	3.22503E-5	17	193	1070	41255	over
GO:0032940	secretion by cell	8.76656E-4	0.0134963	3.72044E-5	86	2075	1001	39373	over

GO:0046903	secretion	8.76656E-4	0.0134963	3.72044E-5	86	2075	1001	39373	over
GO:0006796	phosphate metabolic process	9.28388E-4	0.0145158	3.98606E-5	97	2419	990	39029	over
GO:0008629	induction of apoptosis by intracellular signals	0.00161243	0.02667	6.53068E-5	3	1	1084	41447	over
GO:0009904	chloroplast accumulation movement	0.00161243	0.02667	6.53068E-5	3	1	1084	41447	over
GO:0009903	chloroplast avoidance movement	0.00161243	0.02667	6.53068E-5	3	1	1084	41447	over
GO:0008631	induction of apoptosis by oxidative stress	0.00161243	0.02667	6.53068E-5	3	1	1084	41447	over
GO:0044437	vacuolar part	0.00161243	0.0270392	6.79576E-5	14	146	1073	41302	over
GO:0000178	exosome (RNase complex)	0.00165996	0.0284243	7.1678E-5	13	128	1074	41320	over
GO:0042803	protein homodimerization activity	0.00165996	0.0286315	7.23338E-5	9	61	1078	41387	over
GO:0019904	protein domain specific binding	0.00170129	0.0297467	7.69546E-5	10	77	1077	41371	over
GO:0000160	two-component signal transduction system (phosphorelay)	0.00184124	0.0325993	7.99315E-5	18	230	1069	41218	over
GO:0007165	signal transduction	0.00187232	0.0335929	8.48474E-5	82	2004	1005	39444	over
GO:0016043	cellular component organization and biogenesis	0.00190944	0.0347082	8.994E-5	200	5882	887	35566	over
GO:0004553	hydrolase activity, hydrolyzing O-glycosyl compounds	0.00198475	0.0365303	9.50293E-5	39	754	1048	40694	over
GO:0016787	hydrolase activity	0.00211964	0.0394733	1.03309E-4	200	5897	887	35551	over
GO:0005769	early endosome	0.00248233	0.047253	1.2161E-4	5	15	1082	41433	over

GO:0010200	response to chitin	0.00248233	0.047253	1.2161E-4	5	15	1082	41433	over
GO:0009737	response to abscisic acid stimulus	0.00279635	0.0537312	1.54277E-4	27	457	1060	40991	over
GO:0009341	beta-galactosidase complex	0.00291702	0.0566719	1.57361E-4	8	53	1079	41395	over
GO:0010231	maintenance of seed dormancy	0.00322981	0.0633115	1.6015E-4	3	2	1084	41446	over
GO:0004565	beta-galactosidase activity	0.00337778	0.0669024	1.76664E-4	8	54	1079	41394	over
GO:0005543	phospholipid binding	0.00373595	0.0745935	1.97419E-4	11	105	1076	41343	over
GO:0022838	substrate specific channel activity	0.00384557	0.0784404	2.02837E-4	15	184	1072	41264	over
GO:0007264	small GTPase mediated signal transduction	0.00384557	0.0784696	2.02869E-4	22	342	1065	41106	over
GO:0016798	hydrolase activity, acting on glycosyl bonds	0.00394541	0.0813294	2.13993E-4	40	814	1047	40634	over
GO:0009902	chloroplast relocation	0.00451132	0.0955052	2.52193E-4	4	9	1083	41439	over
GO:0051644	plastid localization	0.00451132	0.0955052	2.52193E-4	4	9	1083	41439	over
GO:0051667	establishment of plastid localization	0.00451132	0.0955052	2.52193E-4	4	9	1083	41439	over
GO:0015925	galactosidase activity	0.00469459	0.100243	2.74353E-4	8	58	1079	41390	over
GO:0000161	MAPKKK cascade during osmolarity sensing	0.00544683	0.123737	3.14194E-4	3	3	1084	41445	over
GO:0043405	regulation of MAPK activity	0.00544683	0.123737	3.14194E-4	3	3	1084	41445	over
GO:0000169	activation of MAPK activity during osmolarity sensing	0.00544683	0.123737	3.14194E-4	3	3	1084	41445	over

GO:0000187	activation of MAPK activity	0.00544683	0.123737	3.14194E-4	3	3	1084	41445	over
GO:0043406	positive regulation of MAPK activity	0.00544683	0.123737	3.14194E-4	3	3	1084	41445	over
GO:0007231	osmosensory signaling pathway	0.00544683	0.123737	3.14194E-4	3	3	1084	41445	over
GO:0009898	internal side of plasma membrane	0.00544683	0.123737	3.14194E-4	3	3	1084	41445	over
GO:0009628	response to abiotic stimulus	0.00555832	0.127316	3.30494E-4	86	2222	1001	39226	over
GO:0065002	intracellular protein transport across a membrane	0.00581082	0.135215	3.45933E-4	4	10	1083	41438	over
GO:0000060	protein import into nucleus, translocation	0.00581082	0.135215	3.45933E-4	4	10	1083	41438	over
GO:0009409	response to cold	0.00684122	0.158649	4.47942E-4	27	491	1060	40957	over
GO:0012505	endomembrane system	0.00701347	0.163768	4.5692E-4	88	2310	999	39138	over
GO:0010262	somatic embryogenesis	0.00737026	0.172868	4.62201E-4	4	11	1083	41437	over
GO:0009612	response to mechanical stimulus	0.00769445	0.181321	5.01717E-4	6	34	1081	41414	over
GO:0006898	receptor-mediated endocytosis	0.00955851	0.221917	6.03834E-4	4	12	1083	41436	over
GO:0015115	silicate transmembrane transporter activity	0.0109514	0.258026	6.52494E-4	2	0	1085	41448	over
GO:0044426	cell wall part	0.0109514	0.258026	6.52494E-4	2	0	1085	41448	over
GO:0048226	Casparian strip	0.0109514	0.258026	6.52494E-4	2	0	1085	41448	over
GO:0015708	silicate transport	0.0109514	0.258026	6.52494E-4	2	0	1085	41448	over

GO:0005941	unlocalized protein complex	0.011326	0.268158	7.06357E-4	10	104	1077	41344	over
GO:0032555	purine ribonucleotide binding	0.011326	0.272145	7.27244E-4	150	4404	937	37044	over
GO:0032553	ribonucleotide binding	0.011326	0.272145	7.27244E-4	150	4404	937	37044	over
GO:0046961	hydrogen ion transporting ATPase activity, rotational mechanism	0.011326	0.273835	7.46665E-4	16	233	1071	41215	over
GO:0045298	tubulin complex	0.0116834	0.283228	7.69881E-4	5	24	1082	41424	over
GO:0042631	cellular response to water deprivation	0.0118573	0.288883	7.73711E-4	4	13	1083	41435	over
GO:0004674	protein serine/threonine kinase activity	0.011891	0.29168	7.90687E-4	62	1532	1025	39916	over
GO:0047958	glycine transaminase activity	0.0128624	0.315773	8.46579E-4	3	5	1084	41443	over
GO:0000299	integral to membrane of membrane fraction	0.0128624	0.315773	8.46579E-4	3	5	1084	41443	over
GO:0016310	phosphorylation	0.0130675	0.32212	8.88862E-4	81	2138	1006	39310	over
GO:0005372	water transporter activity	0.0133915	0.333104	9.1648E-4	7	54	1080	41394	over
GO:0015250	water channel activity	0.0133915	0.333104	9.1648E-4	7	54	1080	41394	over
GO:0051656	establishment of organelle localization	0.0159745	0.389616	0.00120982	4	15	1083	41433	over
GO:0051640	organelle localization	0.0159745	0.389616	0.00120982	4	15	1083	41433	over
GO:0009260	ribonucleotide biosynthetic process	0.0159745	0.390584	0.00121773	19	318	1068	41130	over
GO:0046474	glycerophospholipid biosynthetic process	0.0160567	0.39457	0.00122167	7	57	1080	41391	over

GO:0044462	external encapsulating structure part	0.0167778	0.419233	0.00124575	3	6	1084	41442	over
GO:0051748	UDP-sugar pyrophosphorylase activity	0.0167778	0.419233	0.00124575	3	6	1084	41442	over
GO:0003983	UTP:glucose-1-phosphate uridylyltransferase activity	0.0167778	0.419233	0.00124575	3	6	1084	41442	over
GO:0008474	palmitoyl-(protein) hydrolase activity	0.0167778	0.419233	0.00124575	3	6	1084	41442	over
GO:0009259	ribonucleotide metabolic process	0.0167778	0.42035	0.00126015	19	319	1068	41129	over
GO:0048037	cofactor binding	0.0180225	0.445839	0.00147331	41	934	1046	40514	over
GO:0016844	strictosidine synthase activity	0.0182512	0.458274	0.00148187	4	16	1083	41432	over
GO:0008422	beta-glucosidase activity	0.0182512	0.458274	0.00148187	4	16	1083	41432	over
GO:0016843	amine-lyase activity	0.0182512	0.458274	0.00148187	4	16	1083	41432	over
GO:0022892	substrate-specific transporter activity	0.0182512	0.459924	0.0014993	66	1701	1021	39747	over
GO:0046983	protein dimerization activity	0.0183607	0.468846	0.00154199	19	325	1068	41123	over
GO:0015985	energy coupled proton transport, down electrochemical gradient	0.0183607	0.469278	0.00154759	16	251	1071	41197	over
GO:0015986	ATP synthesis coupled proton transport	0.0183607	0.469278	0.00154759	16	251	1071	41197	over
GO:0006886	intracellular protein transport	0.0187527	0.478918	0.0016244	38	851	1049	40597	over
GO:0005886	plasma membrane	0.0187527	0.481298	0.00166243	37	823	1050	40625	over
GO:0009882	blue light photoreceptor activity	0.0216386	0.533672	0.00179378	4	17	1083	41431	over

GO:0019740	nitrogen utilization	0.0219404	0.543644	0.0018547	5	30	1082	41418	over
GO:0006808	regulation of nitrogen utilization	0.0219404	0.543644	0.0018547	5	30	1082	41418	over
GO:0008863	formate dehydrogenase activity	0.0239189	0.593976	0.00192419	2	1	1085	41447	over
GO:0010329	auxin efflux transmembrane transporter activity	0.0239189	0.593976	0.00192419	2	1	1085	41447	over
GO:0016328	lateral plasma membrane	0.0239189	0.593976	0.00192419	2	1	1085	41447	over
GO:0010082	regulation of root meristem size	0.0239189	0.593976	0.00192419	2	1	1085	41447	over
GO:0016469	proton-transporting two-sector ATPase complex	0.0239189	0.594681	0.00193907	16	257	1071	41191	over
GO:0006754	ATP biosynthetic process	0.0239189	0.594681	0.00193907	16	257	1071	41191	over
GO:0046034	ATP metabolic process	0.0239189	0.594681	0.00193907	16	257	1071	41191	over
GO:0006753	nucleoside phosphate metabolic process	0.0239189	0.594681	0.00193907	16	257	1071	41191	over
GO:0017076	purine nucleotide binding	0.0241165	0.600113	0.00202224	153	4625	934	36823	over
GO:0009269	response to desiccation	0.0257133	0.628874	0.00226623	6	47	1081	41401	over
GO:0005515	protein binding	0.0257133	0.629781	0.00227737	137	4090	950	37358	over
GO:0007242	intracellular signaling cascade	0.0257133	0.63178	0.00231996	51	1263	1036	40185	over
GO:0019829	cation-transporting ATPase activity	0.0257133	0.633221	0.00233453	17	287	1070	41161	over
GO:0000165	MAPKKK cascade	0.0266631	0.651245	0.00235513	3	8	1084	41440	over

GO:0007030	Golgi organization and biogenesis	0.0266631	0.651245	0.00235513	3	8	1084	41440	over
GO:0051171	regulation of nitrogen metabolic process	0.0268382	0.655967	0.00238793	5	32	1082	41416	over
GO:0019842	vitamin binding	0.0277062	0.669935	0.00254811	18	315	1069	41133	over
GO:0043067	regulation of programmed cell death	0.0280716	0.677001	0.0026302	7	66	1080	41382	over
GO:0031225	anchored to membrane	0.0287142	0.687504	0.00269264	5	33	1082	41415	over
GO:0042981	regulation of apoptosis	0.0288859	0.691905	0.00274121	6	49	1081	41399	over
GO:0009206	purine ribonucleoside triphosphate biosynthetic process	0.0293778	0.702427	0.00297258	16	269	1071	41179	over
GO:0009205	purine ribonucleoside triphosphate metabolic process	0.0293778	0.702427	0.00297258	16	269	1071	41179	over
GO:0004335	galactokinase activity	0.0297785	0.713775	0.00299702	4	20	1083	41428	over
GO:0006897	endocytosis	0.0297785	0.713775	0.00299702	4	20	1083	41428	over
GO:0010324	membrane invagination	0.0297785	0.713775	0.00299702	4	20	1083	41428	over
GO:0046933	hydrogen ion transporting ATP synthase activity, rotational mechanism	0.0298611	0.722781	0.00306336	15	245	1072	41203	over
GO:0009199	ribonucleoside triphosphate metabolic process	0.0298611	0.725222	0.00307611	16	270	1071	41178	over
GO:0009144	purine nucleoside triphosphate metabolic process	0.0298611	0.725222	0.00307611	16	270	1071	41178	over
GO:0009145	purine nucleoside triphosphate biosynthetic process	0.0298611	0.725222	0.00307611	16	270	1071	41178	over
GO:0009201	ribonucleoside triphosphate biosynthetic process	0.0298611	0.725222	0.00307611	16	270	1071	41178	over

GO:0016773	phosphotransferase activity, alcohol group as acceptor	0.0317012	0.748255	0.00312764	89	2507	998	38941	over
GO:0022891	substrate-specific transmembrane transporter activity	0.031741	0.750678	0.00320684	53	1347	1034	40101	over
GO:0005516	calmodulin binding	0.0323496	0.759189	0.00335982	19	350	1068	41098	over
GO:0006541	glutamine metabolic process	0.0336164	0.775027	0.00355683	7	70	1080	41378	over
GO:0016301	kinase activity	0.0336164	0.77605	0.00357988	104	3021	983	38427	over
GO:0009142	nucleoside triphosphate biosynthetic process	0.0344169	0.785744	0.00376175	16	276	1071	41172	over
GO:0004656	procollagen-proline 4-dioxygenase activity	0.0380703	0.827852	0.00378308	2	2	1085	41446	over
GO:0019798	procollagen-proline dioxygenase activity	0.0380703	0.827852	0.00378308	2	2	1085	41446	over
GO:0031176	endo-1,4-beta-xylanase activity	0.0380703	0.827852	0.00378308	2	2	1085	41446	over
GO:0031543	peptidyl-proline dioxygenase activity	0.0380703	0.827852	0.00378308	2	2	1085	41446	over
GO:0031545	peptidyl-proline 4-dioxygenase activity	0.0380703	0.827852	0.00378308	2	2	1085	41446	over
GO:0022610	biological adhesion	0.0380703	0.829806	0.00382308	7	71	1080	41377	over
GO:0007155	cell adhesion	0.0380703	0.829806	0.00382308	7	71	1080	41377	over
GO:0006644	phospholipid metabolic process	0.0383436	0.83356	0.0039102	12	179	1075	41269	over
GO:0007243	protein kinase cascade	0.0400014	0.847512	0.00392935	3	10	1084	41438	over
GO:0009820	alkaloid metabolic process	0.0407166	0.855529	0.0040496	4	22	1083	41426	over

GO:0009821	alkaloid biosynthetic process	0.0407166	0.855529	0.0040496	4	22	1083	41426	over
GO:0009743	response to carbohydrate stimulus	0.0411428	0.861363	0.00424841	12	181	1075	41267	over
GO:0009141	nucleoside triphosphate metabolic process	0.0411428	0.862842	0.00428503	16	280	1071	41168	over
GO:0015031	protein transport	0.0411428	0.863128	0.00430608	42	1026	1045	40422	over
GO:0009066	aspartate family amino acid metabolic process	0.0411428	0.864137	0.00434433	15	255	1072	41193	over
GO:0004022	alcohol dehydrogenase activity	0.0412909	0.866497	0.00442767	8	93	1079	41355	over
GO:0000234	phosphoethanolamine N-methyltransferase activity	0.0457653	0.893899	0.00490665	3	11	1084	41437	over
GO:0019107	myristoyltransferase activity	0.0471193	0.90697	0.00532697	4	24	1083	41424	over
GO:0048040	UDP-glucuronate decarboxylase activity	0.0471193	0.90697	0.00532697	4	24	1083	41424	over
GO:0048196	middle lamella-containing extracellular matrix	0.0471193	0.90697	0.00532697	4	24	1083	41424	over
GO:0006656	phosphatidylcholine biosynthetic process	0.0471193	0.90697	0.00532697	4	24	1083	41424	over
GO:0009881	photoreceptor activity	0.0471193	0.90697	0.00532697	4	24	1083	41424	over
GO:0045184	establishment of protein localization	0.0471193	0.907496	0.00536471	42	1040	1045	40408	over
GO:0010033	response to organic substance	0.0471922	0.908918	0.00540573	12	187	1075	41261	over
GO:0008104	protein localization	0.0472514	0.910259	0.00544812	42	1041	1045	40407	over
GO:0006807	nitrogen compound metabolic process	0.0474037	0.912004	0.00557955	54	1419	1033	40029	over

GO:0016840	carbon-nitrogen lyase activity	0.0486644	0.919499	0.00584536	6	58	1081	41390	over
GO:0009637	response to blue light	0.0486644	0.919499	0.00584536	6	58	1081	41390	over

GOSSIP
Test-Set: yuc.10.20.up.txt
Tests for all terms in Gene Ontology whether it is enriched in a test group when compared to a reference group using Fisher's excact test with Multiple Testing.
Pub: Biological Profiling of Gene Groups utilizing Gene Ontology A Statistical Framework
Poster: GOSSIP: Biological Profiling of Gene Groups utilizing Gene Ontology
by Nils Blthgen, Karsten Brand, Hanspeter Herzel, Dieter Beule

GO Term	Name	FDR	FWER	single test p-Value	# in test group	# in reference group	# non annot test	# non annot reference group	Over/Under
GO:0016023	cytoplasmic membrane-bound vesicle	2.64247E-8	3.71921E-8	0.0	189	4970	723	36653	over
GO:0031410	cytoplasmic vesicle	2.64247E-8	3.71921E-8	0.0	189	4970	723	36653	over
GO:0004553	hydrolase activity, hydrolyzing O-glycosyl compounds	2.64247E-8	3.71921E-8	0.0	57	736	855	40887	over
GO:0016798	hydrolase activity, acting on glycosyl bonds	2.64247E-8	3.71921E-8	0.0	59	795	853	40828	over
GO:0003824	catalytic activity	2.64247E-8	3.71921E-8	0.0	500	18009	412	23614	over
GO:0031982	vesicle	2.64247E-8	4.62432E-8	3.24972E-11	189	4972	723	36651	over
GO:0031988	membrane-bound vesicle	2.64247E-8	4.62432E-8	3.24972E-11	189	4972	723	36651	over
GO:0000287	magnesium ion binding	1.32316E-7	2.64632E-7	7.90459E-10	35	457	877	41166	over

GO:0019748	secondary metabolic process	7.04453E-7	1.58502E-6	4.86561E-9	47	802	865	40821	over
GO:0009147	pyrimidine nucleoside triphosphate metabolic process	1.33079E-6	3.32696E-6	1.06291E-8	8	14	904	41609	over
GO:0009341	beta-galactosidase complex	6.67239E-6	1.83489E-5	6.52423E-8	11	50	901	41573	over
GO:0004565	beta-galactosidase activity	7.3704E-6	2.2111E-5	7.77671E-8	11	51	901	41572	over
GO:0015925	galactosidase activity	1.50236E-5	4.88257E-5	1.52016E-7	11	55	901	41568	over
GO:0009045	xylose isomerase activity	5.14424E-5	1.80032E-4	5.25804E-7	5	4	907	41619	over
GO:0006629	lipid metabolic process	6.617E-5	2.48107E-4	7.82971E-7	60	1369	852	40254	over
GO:0009220	pyrimidine ribonucleotide biosynthetic process	7.06935E-5	3.00402E-4	9.66415E-7	6	11	906	41612	over
GO:0009218	pyrimidine ribonucleotide metabolic process	7.06935E-5	3.00402E-4	9.66415E-7	6	11	906	41612	over
GO:0030312	external encapsulating structure	9.14542E-5	4.11459E-4	1.18712E-6	27	410	885	41213	over
GO:0017171	serine hydrolase activity	1.53967E-4	7.69538E-4	2.38131E-6	25	375	887	41248	over
GO:0008236	serine-type peptidase activity	1.53967E-4	7.69538E-4	2.38131E-6	25	375	887	41248	over
GO:0045552	dihydrokaempferol 4-reductase activity	1.72721E-4	0.00111329	3.04317E-6	4	2	908	41621	over
GO:0046051	UTP metabolic process	1.72721E-4	0.00120832	3.13233E-6	5	7	907	41616	over
GO:0006241	CTP biosynthetic process	1.72721E-4	0.00120832	3.13233E-6	5	7	907	41616	over
GO:0046036	CTP metabolic process	1.72721E-4	0.00120832	3.13233E-6	5	7	907	41616	over

GO:0004550	nucleoside diphosphate kinase activity	1.72721E-4	0.00120832	3.13233E-6	5	7	907	41616	over
GO:0006228	UTP biosynthetic process	1.72721E-4	0.00120832	3.13233E-6	5	7	907	41616	over
GO:0046039	GTP metabolic process	1.72721E-4	0.00120832	3.13233E-6	5	7	907	41616	over
GO:0006183	GTP biosynthetic process	1.72721E-4	0.00120832	3.13233E-6	5	7	907	41616	over
GO:0006575	amino acid derivative metabolic process	1.83616E-4	0.00133033	3.81289E-6	33	603	879	41020	over
GO:0009209	pyrimidine ribonucleoside triphosphate biosynthetic process	2.25498E-4	0.00174609	4.99987E-6	5	8	907	41615	over
GO:0009208	pyrimidine ribonucleoside triphosphate metabolic process	2.25498E-4	0.00174609	4.99987E-6	5	8	907	41615	over
GO:0042398	amino acid derivative biosynthetic process	2.35257E-4	0.00188029	5.39797E-6	28	474	884	41149	over
GO:0005576	extracellular region	3.54449E-4	0.00291994	8.4868E-6	24	379	888	41244	over
GO:0016711	flavonoid 3'-monooxygenase activity	4.44353E-4	0.00376988	9.82525E-6	3	0	909	41623	over
GO:0008810	cellulase activity	6.2464E-4	0.0054507	1.439E-5	7	31	905	41592	over
GO:0047461	(+)-delta-cadinene synthase activity	6.81713E-4	0.00611664	1.60845E-5	5	11	907	41612	over
GO:0004289	subtilase activity	7.23481E-4	0.00666987	1.84773E-5	13	134	899	41489	over
GO:0000015	phosphopyruvate hydratase complex	0.00108779	0.0103027	2.97775E-5	6	23	906	41600	over
GO:0005941	unlocalized protein complex	0.00108779	0.0118173	3.58974E-5	11	103	901	41520	over
GO:0047429	nucleoside-triphosphate diphosphatase activity	0.00108779	0.0148459	3.86713E-5	3	1	909	41622	over

GO:0008631	induction of apoptosis by oxidative stress	0.00108779	0.0148459	3.86713E-5	3	1	909	41622	over
GO:0019401	alditol biosynthetic process	0.00108779	0.0148459	3.86713E-5	3	1	909	41622	over
GO:0019593	mannitol biosynthetic process	0.00108779	0.0148459	3.86713E-5	3	1	909	41622	over
GO:0030497	fatty acid elongation	0.00108779	0.0148459	3.86713E-5	3	1	909	41622	over
GO:0019594	mannitol metabolic process	0.00108779	0.0148459	3.86713E-5	3	1	909	41622	over
GO:0019406	hexitol biosynthetic process	0.00108779	0.0148459	3.86713E-5	3	1	909	41622	over
GO:0046173	polyol biosynthetic process	0.00108779	0.0148459	3.86713E-5	3	1	909	41622	over
GO:0047268	galactinol-raffinose galactosyltransferase activity	0.00108779	0.0148459	3.86713E-5	3	1	909	41622	over
GO:0008629	induction of apoptosis by intracellular signals	0.00108779	0.0148459	3.86713E-5	3	1	909	41622	over
GO:0019400	alditol metabolic process	0.00108779	0.0148459	3.86713E-5	3	1	909	41622	over
GO:0009394	2'-deoxyribonucleotide metabolic process	0.00108779	0.0148459	3.86713E-5	3	1	909	41622	over
GO:0006059	hexitol metabolic process	0.00108779	0.0148459	3.86713E-5	3	1	909	41622	over
GO:0046080	dUTP metabolic process	0.00108779	0.0148459	3.86713E-5	3	1	909	41622	over
GO:0004170	dUTP diphosphatase activity	0.00108779	0.0148459	3.86713E-5	3	1	909	41622	over
GO:0010325	raffinose family oligosaccharide biosynthetic process	0.00108779	0.0148459	3.86713E-5	3	1	909	41622	over
GO:0019438	aromatic compound biosynthetic process	0.00116029	0.0161129	3.9956E-5	23	392	889	41231	over

GO:0005618	cell wall	0.00127105	0.0179495	4.62648E-5	23	396	889	41227	over
GO:0016746	transferase activity, transferring acyl groups	0.00127263	0.0182839	4.83253E-5	31	628	881	40995	over
GO:0004634	phosphopyruvate hydratase activity	0.0013955	0.0203733	5.37786E-5	6	26	906	41597	over
GO:0016787	hydrolase activity	0.00143933	0.0213587	5.93849E-5	173	5924	739	35699	over
GO:0009698	phenylpropanoid metabolic process	0.00158188	0.024081	6.51748E-5	22	378	890	41245	over
GO:0006220	pyrimidine nucleotide metabolic process	0.00158188	0.024607	6.66566E-5	9	73	903	41550	over
GO:0004650	polygalacturonase activity	0.00158188	0.024607	6.66566E-5	9	73	903	41550	over
GO:0009835	ripening	0.00168851	0.0266546	7.42804E-5	8	57	904	41566	over
GO:0008415	acyltransferase activity	0.00178996	0.0286681	8.46625E-5	28	558	884	41065	over
GO:0009343	biotin carboxylase complex	0.00238645	0.0386116	9.51301E-5	3	2	909	41621	over
GO:0006519	amino acid and derivative metabolic process	0.00238855	0.0394042	1.00053E-4	60	1623	852	40000	over
GO:0016801	hydrolase activity, acting on ether bonds	0.00238855	0.0404041	1.03676E-4	7	44	905	41579	over
GO:0004180	carboxypeptidase activity	0.00238855	0.0407747	1.05499E-4	10	97	902	41526	over
GO:0007167	enzyme linked receptor protein signaling pathway	0.00238855	0.0425533	1.12325E-4	17	259	895	41364	over
GO:0007169	transmembrane receptor protein tyrosine kinase signaling pathway	0.00238855	0.0425533	1.12325E-4	17	259	895	41364	over
GO:0016747	transferase activity, transferring groups other than amino-acyl groups	0.00238855	0.0426167	1.12462E-4	28	568	884	41055	over

GO:0009813	flavonoid biosynthetic process	0.00238855	0.0426549	1.12846E-4	13	162	899	41461	over
GO:0004185	serine carboxypeptidase activity	0.00251163	0.0454027	1.26466E-4	8	62	904	41561	over
GO:0008195	phosphatidate phosphatase activity	0.00263791	0.048258	1.28635E-4	4	9	908	41614	over
GO:0009505	cellulose and pectin-containing cell wall	0.00275945	0.0510793	1.38518E-4	18	290	894	41333	over
GO:0006725	aromatic compound metabolic process	0.0035225	0.0655612	1.85921E-4	32	711	880	40912	over
GO:0008883	glutamyl-tRNA reductase activity	0.00390656	0.0742542	1.87217E-4	3	3	909	41620	over
GO:0009312	oligosaccharide biosynthetic process	0.00390656	0.0742542	1.87217E-4	3	3	909	41620	over
GO:0009612	response to mechanical stimulus	0.0039746	0.0764158	1.96901E-4	6	34	906	41589	over
GO:0009699	phenylpropanoid biosynthetic process	0.00408858	0.0794602	2.05295E-4	18	300	894	41323	over
GO:0005975	carbohydrate metabolic process	0.00409376	0.0813005	2.16517E-4	77	2295	835	39328	over
GO:0046148	pigment biosynthetic process	0.00409376	0.081439	2.17332E-4	15	223	897	41400	over
GO:0004091	carboxylesterase activity	0.00425367	0.0854551	2.36945E-4	20	359	892	41264	over
GO:0030675	Rac GTPase activator activity	0.00443439	0.0899288	2.37315E-4	4	11	908	41612	over
GO:0004252	serine-type endopeptidase activity	0.00459108	0.0939946	2.47419E-4	17	278	895	41345	over
GO:0009812	flavonoid metabolic process	0.00476821	0.098514	2.68006E-4	13	178	899	41445	over
GO:0048196	middle lamella-containing extracellular matrix	0.00497997	0.103773	2.92375E-4	5	23	907	41600	over

GO:0016838	carbon-oxygen lyase activity, acting on phosphates	0.00502033	0.105692	2.96141E-4	6	37	906	41586	over
GO:0042440	pigment metabolic process	0.00760137	0.157209	4.5826E-4	16	267	896	41356	over
GO:0004314	[acyl-carrier-protein] S-malonyltransferase activity	0.00795132	0.177476	4.5923E-4	2	0	910	41623	over
GO:0015708	silicate transport	0.00795132	0.177476	4.5923E-4	2	0	910	41623	over
GO:0015115	silicate transmembrane transporter activity	0.00795132	0.177476	4.5923E-4	2	0	910	41623	over
GO:0044426	cell wall part	0.00795132	0.177476	4.5923E-4	2	0	910	41623	over
GO:0016420	malonyltransferase activity	0.00795132	0.177476	4.5923E-4	2	0	910	41623	over
GO:0048226	Casparian strip	0.00795132	0.177476	4.5923E-4	2	0	910	41623	over
GO:0010023	proanthocyanidin biosynthetic process	0.00795132	0.177476	4.5923E-4	2	0	910	41623	over
GO:0016419	S-malonyltransferase activity	0.00795132	0.177476	4.5923E-4	2	0	910	41623	over
GO:0016298	lipase activity	0.00795132	0.178647	4.72381E-4	14	215	898	41408	over
GO:0047274	galactinol-sucrose galactosyltransferase activity	0.00900783	0.202802	5.07599E-4	3	5	909	41618	over
GO:0016846	carbon-sulfur lyase activity	0.00900783	0.203443	5.0768E-4	9	98	903	41525	over
GO:0016740	transferase activity	0.00991492	0.22341	6.1723E-4	175	6300	737	35323	over
GO:0005100	Rho GTPase activator activity	0.010123	0.229471	6.29394E-4	4	15	908	41608	over
GO:0016847	1-aminocyclopropane-1-carboxylate synthase activity	0.0104989	0.240891	6.79883E-4	6	44	906	41579	over

GO:0000038	very-long-chain fatty acid metabolic process	0.0104989	0.240891	6.79883E-4	6	44	906	41579	over
GO:0016020	membrane	0.010737	0.247645	7.39947E-4	258	9834	654	31789	over
GO:0009211	pyrimidine deoxyribonucleoside triphosphate metabolic process	0.0112468	0.265558	7.4926E-4	3	6	909	41617	over
GO:0009120	deoxyribonucleoside metabolic process	0.0112468	0.265558	7.4926E-4	3	6	909	41617	over
GO:0046125	pyrimidine deoxyribonucleoside metabolic process	0.0112468	0.265558	7.4926E-4	3	6	909	41617	over
GO:0012505	endomembrane system	0.0112468	0.266043	7.51299E-4	75	2323	837	39300	over
GO:0050737	O-hydroxycinnamoyltransferase activity	0.0116394	0.280237	7.73451E-4	4	16	908	41607	over
GO:0050734	hydroxycinnamoyltransferase activity	0.0116394	0.280237	7.73451E-4	4	16	908	41607	over
GO:0047205	quinate O-hydroxycinnamoyltransferase activity	0.0116394	0.280237	7.73451E-4	4	16	908	41607	over
GO:0016563	transcription activator activity	0.0117681	0.284957	8.21737E-4	13	202	899	41421	over
GO:0006631	fatty acid metabolic process	0.0123338	0.29856	8.88523E-4	25	555	887	41068	over
GO:0009200	deoxyribonucleoside triphosphate metabolic process	0.0147433	0.350321	0.00105333	3	7	909	41616	over
GO:0005815	microtubule organizing center	0.0147433	0.350321	0.00105333	3	7	909	41616	over
GO:0009064	glutamine family amino acid metabolic process	0.015134	0.360131	0.00111739	11	158	901	41465	over
GO:0044271	nitrogen compound biosynthetic process	0.0161375	0.381299	0.00123029	26	601	886	41022	over
GO:0015926	glucosidase activity	0.0164848	0.39018	0.00125136	5	33	907	41590	over

GO:0006541	glutamine metabolic process	0.0167808	0.398102	0.00133251	7	70	905	41553	over
GO:0010241	ent-kaurene oxidase activity	0.0192116	0.44346	0.00135804	2	1	910	41622	over
GO:0031012	extracellular matrix	0.0196618	0.453743	0.00141032	5	34	907	41589	over
GO:0009317	acetyl-CoA carboxylase complex	0.0204173	0.471002	0.00142528	3	8	909	41615	over
GO:0019752	carboxylic acid metabolic process	0.0204173	0.471766	0.0014336	67	2076	845	39547	over
GO:0006082	organic acid metabolic process	0.0204173	0.474403	0.0014501	67	2077	845	39546	over
GO:0042732	D-xylose metabolic process	0.0212155	0.490168	0.00158367	5	35	907	41588	over
GO:0004013	adenosylhomocysteinase activity	0.0212453	0.497792	0.00158477	4	20	908	41603	over
GO:0016802	trialkylsulfonium hydrolase activity	0.0212453	0.497792	0.00158477	4	20	908	41603	over
GO:0016705	oxidoreductase activity, acting on paired donors, with incorporation or reduction of molecular oxygen	0.0212453	0.498704	0.00160421	17	332	895	41291	over
GO:0003993	acid phosphatase activity	0.0214428	0.505135	0.00166587	7	73	905	41550	over
GO:0044255	cellular lipid metabolic process	0.0214428	0.507225	0.00170575	43	1202	869	40421	over
GO:0009826	unidimensional cell growth	0.0216855	0.51414	0.00178619	14	249	898	41374	over
GO:0045892	negative regulation of transcription, DNA-dependent	0.0216855	0.51643	0.00179029	7	74	905	41549	over
GO:0050113	inositol oxygenase activity	0.0232061	0.550997	0.00187018	3	9	909	41614	over
GO:0016701	oxidoreductase activity, acting on single donors with incorporation of	0.0232061	0.550997	0.00187018	3	9	909	41614	over

	molecular oxygen								
GO:0009311	oligosaccharide metabolic process	0.0232061	0.550997	0.00187018	3	9	909	41614	over
GO:0008393	fatty acid (omega-1)-hydroxylase activity	0.0232061	0.550997	0.00187018	3	9	909	41614	over
GO:0004497	monooxygenase activity	0.0232919	0.554925	0.00191854	17	338	895	41285	over
GO:0007166	cell surface receptor linked signal transduction	0.0234853	0.560496	0.00197291	18	369	894	41254	over
GO:0016861	intramolecular oxidoreductase activity, interconverting aldoses and ketoses	0.0244431	0.577579	0.00211775	6	56	906	41567	over
GO:0006542	glutamine biosynthetic process	0.0249232	0.588653	0.00215527	4	22	908	41601	over
GO:0006721	terpenoid metabolic process	0.0249232	0.589815	0.00216725	10	147	902	41476	over
GO:0003838	sterol 24-C-methyltransferase activity	0.0271421	0.626216	0.00239264	3	10	909	41613	over
GO:0004617	phosphoglycerate dehydrogenase activity	0.0271421	0.626216	0.00239264	3	10	909	41613	over
GO:0005615	extracellular space	0.0282136	0.64299	0.00248759	4	23	908	41600	over
GO:0042389	omega-3 fatty acid desaturase activity	0.0326988	0.704265	0.00267741	2	2	910	41621	over
GO:0033559	unsaturated fatty acid metabolic process	0.0326988	0.704265	0.00267741	2	2	910	41621	over
GO:0006636	unsaturated fatty acid biosynthetic process	0.0326988	0.704265	0.00267741	2	2	910	41621	over
GO:0006012	galactose metabolic process	0.0329213	0.709112	0.00269592	6	59	906	41564	over
GO:0032787	monocarboxylic acid metabolic process	0.0333352	0.715972	0.00284684	31	811	881	40812	over

GO:0009399	nitrogen fixation	0.033688	0.722083	0.00285346	4	24	908	41599	over
GO:0015995	chlorophyll biosynthetic process	0.0337962	0.725554	0.00290773	8	104	904	41519	over
GO:0004764	shikimate 5-dehydrogenase activity	0.0344129	0.744852	0.0029969	3	11	909	41612	over
GO:0019187	beta-1,4-mannosyltransferase activity	0.0344129	0.744852	0.0029969	3	11	909	41612	over
GO:0000030	mannosyltransferase activity	0.0344129	0.744852	0.0029969	3	11	909	41612	over
GO:0051753	mannan synthase activity	0.0344129	0.744852	0.0029969	3	11	909	41612	over
GO:0004075	biotin carboxylase activity	0.0344129	0.744852	0.0029969	3	11	909	41612	over
GO:0016829	lyase activity	0.0344129	0.745445	0.00301741	41	1169	871	40454	over
GO:0004356	glutamate-ammonia ligase activity	0.0360105	0.763258	0.00325447	4	25	908	41598	over
GO:0015994	chlorophyll metabolic process	0.0379986	0.783435	0.00366019	9	133	903	41490	over
GO:0006917	induction of apoptosis	0.0389029	0.799151	0.00368679	3	12	909	41611	over
GO:0043065	positive regulation of apoptosis	0.0389029	0.799151	0.00368679	3	12	909	41611	over
GO:0043068	positive regulation of programmed cell death	0.0389029	0.799151	0.00368679	3	12	909	41611	over
GO:0012502	induction of programmed cell death	0.0389029	0.799151	0.00368679	3	12	909	41611	over
GO:0016776	phosphotransferase activity, phosphate group as acceptor	0.0406875	0.815302	0.00391186	6	64	906	41559	over
GO:0009932	cell tip growth	0.0408558	0.818453	0.00391871	5	44	907	41579	over

GO:0008169	C-methyltransferase activity	0.0497168	0.876171	0.00446581	3	13	909	41610	over

GOSSIP
Test-Set: yuc.10.20.down.txt
Tests for all terms in Gene Ontology whether it is enriched in a test group when compared to a reference group using Fisher's excact test with Multiple Testing.
Pub: Biological Profiling of Gene Groups utilizing Gene Ontology A Statistical Framework
Poster: GOSSIP: Biological Profiling of Gene Groups utilizing Gene Ontology
by Nils Blthgen, Karsten Brand, Hanspeter Herzel, Dieter Beule

GO Term	Name	FDR	FWER	single test p-Value	# in test group	# in reference group	# non annot test	# non annot reference group	Over/Under
GO:0008194	UDP-glycosyltransferase activity	1.34498E-8	4.52833E-8	0.0	44	515	874	41102	over
GO:0016798	hydrolase activity, acting on glycosyl bonds	1.34498E-8	4.52833E-8	0.0	59	795	859	40822	over
GO:0042546	cell wall biogenesis	1.34498E-8	4.52833E-8	0.0	25	198	893	41419	over
GO:0016758	transferase activity, transferring hexosyl groups	1.34498E-8	4.52833E-8	0.0	67	785	851	40832	over
GO:0009832	cellulose and pectin-containing cell wall biogenesis	1.34498E-8	4.52833E-8	0.0	25	162	893	41455	over
GO:0004553	hydrolase activity, hydrolyzing O-glycosyl compounds	1.34498E-8	4.52833E-8	0.0	58	735	860	40882	over
GO:0035251	UDP-glucosyltransferase activity	1.34498E-8	4.52833E-8	0.0	31	285	887	41332	over
GO:0007047	cell wall organization and biogenesis	1.34498E-8	4.52833E-8	0.0	46	463	872	41154	over
GO:0009830	cell wall modification during abscission	1.34498E-8	4.52833E-8	0.0	8	5	910	41612	over

GO:0045229	external encapsulating structure organization and biogenesis	1.34498E-8	4.52833E-8	0.0	46	466	872	41151	over
GO:0006073	glucan metabolic process	1.34498E-8	4.52833E-8	0.0	43	414	875	41203	over
GO:0046527	glucosyltransferase activity	1.34498E-8	4.52833E-8	0.0	33	305	885	41312	over
GO:0009834	cellulose and pectin-containing secondary cell wall biogenesis	1.34498E-8	4.56613E-8	3.62956E-12	21	21	897	41596	over
GO:0016757	transferase activity, transferring glycosyl groups	1.34498E-8	4.70744E-8	1.45455E-11	72	1038	846	40579	over
GO:0005975	carbohydrate metabolic process	1.35672E-8	5.08768E-8	2.81475E-11	115	2257	803	39360	over
GO:0010382	cell wall metabolic process	1.75979E-8	7.03915E-8	1.31978E-10	15	64	903	41553	over
GO:0005618	cell wall	2.34829E-8	9.98025E-8	2.16533E-10	33	386	885	41231	over
GO:0009250	glucan biosynthetic process	2.3493E-8	1.11592E-7	2.5902E-10	29	306	889	41311	over
GO:0009531	secondary cell wall	2.3493E-8	1.11592E-7	2.59437E-10	8	7	910	41610	over
GO:0016051	carbohydrate biosynthetic process	3.11606E-8	1.55803E-7	4.14921E-10	49	788	869	40829	over
GO:0030312	external encapsulating structure	4.29006E-8	2.34455E-7	6.13406E-10	33	404	885	41213	over
GO:0016760	cellulose synthase (UDP-forming) activity	4.29006E-8	2.35953E-7	6.2413E-10	16	86	902	41531	over
GO:0046556	alpha-N-arabinofuranosidase activity	5.39606E-8	3.10273E-7	9.47008E-10	9	15	909	41602	over
GO:0000271	polysaccharide biosynthetic process	5.65671E-8	3.39402E-7	1.10178E-9	29	324	889	41293	over
GO:0016759	cellulose synthase activity	6.05578E-8	3.78486E-7	1.26505E-9	16	91	902	41526	over

GO:0046658	anchored to plasma membrane	8.11883E-8	5.27724E-7	1.68355E-9	8	10	910	41607	over
GO:0031410	cytoplasmic vesicle	8.51275E-8	6.02615E-7	2.10108E-9	173	4986	745	36631	over
GO:0016023	cytoplasmic membrane-bound vesicle	8.51275E-8	6.02615E-7	2.10108E-9	173	4986	745	36631	over
GO:0031982	vesicle	8.51275E-8	6.38456E-7	2.21256E-9	173	4988	745	36629	over
GO:0031988	membrane-bound vesicle	8.51275E-8	6.38456E-7	2.21256E-9	173	4988	745	36629	over
GO:0044247	cellular polysaccharide catabolic process	8.55949E-8	6.6336E-7	2.38421E-9	15	81	903	41536	over
GO:0005976	polysaccharide metabolic process	1.86536E-7	1.53892E-6	4.51884E-9	16	100	902	41517	over
GO:0000272	polysaccharide catabolic process	1.86536E-7	1.53892E-6	4.51884E-9	16	100	902	41517	over
GO:0031226	intrinsic to plasma membrane	2.5477E-7	2.16554E-6	6.93581E-9	17	119	901	41498	over
GO:0009897	external side of plasma membrane	2.60622E-7	2.28044E-6	7.15679E-9	8	13	910	41604	over
GO:0009044	xylan 1,4-beta-xylosidase activity	3.02072E-7	2.71864E-6	7.87243E-9	6	3	912	41614	over
GO:0009735	response to cytokinin stimulus	4.51751E-7	4.17869E-6	1.33689E-8	17	125	901	41492	over
GO:0009664	cellulose and pectin-containing cell wall organization and biogenesis	4.64861E-7	4.41617E-6	1.48872E-8	26	300	892	41317	over
GO:0010410	hemicellulose metabolic process	7.13116E-7	7.48769E-6	2.46838E-8	8	16	910	41601	over
GO:0010383	cell wall polysaccharide metabolic process	7.13116E-7	7.48769E-6	2.46838E-8	8	16	910	41601	over
GO:0045493	xylan catabolic process	7.13116E-7	7.48769E-6	2.46838E-8	8	16	910	41601	over

GO:0045491	xylan metabolic process	7.13116E-7	7.48769E-6	2.46838E-8	8	16	910	41601	over
GO:0030244	cellulose biosynthetic process	1.10012E-6	1.18262E-5	3.84453E-8	18	153	900	41464	over
GO:0042545	cell wall modification	1.12798E-6	1.24077E-5	4.12497E-8	13	73	905	41544	over
GO:0030243	cellulose metabolic process	1.26959E-6	1.42828E-5	4.61117E-8	18	155	900	41462	over
GO:0016762	xyloglucan:xyloglucosyl transferase activity	1.42165E-6	1.63488E-5	5.46163E-8	13	75	905	41542	over
GO:0048196	middle lamella-containing extracellular matrix	2.68075E-6	3.28387E-5	9.67261E-8	8	20	910	41597	over
GO:0010215	cellulose microfibril organization	2.68075E-6	3.28387E-5	9.67261E-8	8	20	910	41597	over
GO:0009827	cellulose and pectin-containing cell wall modification	2.68075E-6	3.28387E-5	9.67261E-8	8	20	910	41597	over
GO:0044262	cellular carbohydrate metabolic process	4.06551E-6	5.08175E-5	1.58442E-7	56	1169	862	40448	over
GO:0009838	abscission	4.34754E-6	5.54296E-5	1.75393E-7	8	22	910	41595	over
GO:0005750	mitochondrial respiratory chain complex III	9.77506E-6	1.27068E-4	3.92859E-7	8	25	910	41592	over
GO:0032350	regulation of hormone metabolic process	1.03294E-5	1.39438E-4	4.2133E-7	6	9	912	41608	over
GO:0010337	regulation of salicylic acid metabolic process	1.03294E-5	1.39438E-4	4.2133E-7	6	9	912	41608	over
GO:0005507	copper ion binding	1.3929E-5	1.91506E-4	5.6982E-7	19	207	899	41410	over
GO:0010044	response to aluminum ion	1.59419E-5	2.23162E-4	6.61779E-7	6	10	912	41607	over
GO:0031225	anchored to membrane	3.094E-5	4.40798E-4	1.25812E-6	8	30	910	41587	over

GO:0031012	extracellular matrix	3.66997E-5	5.32005E-4	1.55294E-6	8	31	910	41586	over
GO:0008378	galactosyltransferase activity	4.51925E-5	6.66368E-4	1.95874E-6	10	58	908	41559	over
GO:0016679	oxidoreductase activity, acting on diphenols and related substances as donors	5.32954E-5	7.99111E-4	2.47753E-6	14	127	904	41490	over
GO:0042981	regulation of apoptosis	5.37719E-5	8.19686E-4	2.55305E-6	9	46	909	41571	over
GO:0035250	UDP-galactosyltransferase activity	5.81178E-5	9.0042E-4	2.8136E-6	7	23	911	41594	over
GO:0048046	apoplast	8.59998E-5	0.00136077	3.74197E-6	14	132	904	41485	over
GO:0043067	regulation of programmed cell death	8.59998E-5	0.00137505	3.79864E-6	10	63	908	41554	over
GO:0009986	cell surface	1.02095E-4	0.00165768	4.88539E-6	11	81	907	41536	over
GO:0045551	cinnamyl-alcohol dehydrogenase activity	1.07477E-4	0.00179862	5.1619E-6	5	8	913	41609	over
GO:0004373	glycogen (starch) synthase activity	1.07477E-4	0.00179862	5.1619E-6	5	8	913	41609	over
GO:0015154	disaccharide transmembrane transporter activity	1.11887E-4	0.00192819	5.51937E-6	6	16	912	41601	over
GO:0008515	sucrose transmembrane transporter activity	1.11887E-4	0.00192819	5.51937E-6	6	16	912	41601	over
GO:0009636	response to toxin	1.51128E-4	0.00264124	7.87707E-6	13	122	905	41495	over
GO:0009696	salicylic acid metabolic process	1.87181E-4	0.00331694	9.59611E-6	6	18	912	41599	over
GO:0008643	carbohydrate transport	2.71166E-4	0.0048691	1.33842E-5	14	149	904	41468	over
GO:0043682	copper-transporting ATPase activity	2.90133E-4	0.0053644	1.40801E-5	4	4	914	41613	over

GO:0004008	copper-exporting ATPase activity	2.90133E-4	0.0053644	1.40801E-5	4	4	914	41613	over
GO:0045275	respiratory chain complex III	2.90133E-4	0.00549736	1.48316E-5	8	44	910	41573	over
GO:0045285	ubiquinol-cytochrome-c reductase complex	2.90133E-4	0.00549736	1.48316E-5	8	44	910	41573	over
GO:0010214	seed coat development	2.92918E-4	0.00562281	1.50101E-5	7	31	911	41586	over
GO:0043284	biopolymer biosynthetic process	3.82019E-4	0.00750369	2.08083E-5	30	566	888	41051	over
GO:0016740	transferase activity	3.82019E-4	0.0075165	2.09477E-5	186	6289	732	35328	over
GO:0009011	starch synthase activity	4.71736E-4	0.00939036	2.49529E-5	6	22	912	41595	over
GO:0005576	extracellular region	4.95147E-4	0.00997666	2.80881E-5	23	380	895	41237	over
GO:0009725	response to hormone stimulus	4.98346E-4	0.0101641	2.96441E-5	60	1540	858	40077	over
GO:0006825	copper ion transport	4.99175E-4	0.0103044	2.97703E-5	7	35	911	41582	over
GO:0009407	toxin catabolic process	8.21682E-4	0.0173092	4.24505E-5	9	68	909	41549	over
GO:0009404	toxin metabolic process	8.21682E-4	0.0173092	4.24505E-5	9	68	909	41549	over
GO:0009505	cellulose and pectin-containing cell wall	8.7544E-4	0.018646	4.86826E-5	19	289	899	41328	over
GO:0009415	response to water	9.06772E-4	0.0195292	5.37489E-5	24	425	894	41192	over
GO:0015766	disaccharide transport	9.19433E-4	0.0204883	5.48425E-5	5	15	913	41602	over
GO:0045330	aspartyl esterase activity	9.19433E-4	0.0204883	5.48425E-5	5	15	913	41602	over

GO:0015770	sucrose transport	9.19433E-4	0.0204883	5.48425E-5	5	15	913	41602	over
GO:0004364	glutathione transferase activity	9.19433E-4	0.0206999	5.51188E-5	8	54	910	41563	over
GO:0051119	sugar transmembrane transporter activity	9.50747E-4	0.0216299	5.83068E-5	15	195	903	41422	over
GO:0016157	sucrose synthase activity	0.00103746	0.0238325	6.32028E-5	7	40	911	41577	over
GO:0006915	apoptosis	0.00107265	0.0248923	6.83187E-5	10	91	908	41526	over
GO:0005984	disaccharide metabolic process	0.0012709	0.0297331	8.86184E-5	11	114	907	41503	over
GO:0016413	O-acetyltransferase activity	0.00129765	0.0308575	8.98748E-5	5	17	913	41600	over
GO:0016020	membrane	0.00129765	0.0309783	9.22583E-5	267	9825	651	31792	over
GO:0004103	choline kinase activity	0.00139021	0.0334868	9.29201E-5	4	8	914	41609	over
GO:0015144	carbohydrate transmembrane transporter activity	0.00184086	0.0445393	1.20242E-4	15	209	903	41408	over
GO:0006043	glucosamine catabolic process	0.00211451	0.0549938	1.52182E-4	6	32	912	41585	over
GO:0006046	N-acetylglucosamine catabolic process	0.00211451	0.0549938	1.52182E-4	6	32	912	41585	over
GO:0006041	glucosamine metabolic process	0.00211451	0.0549938	1.52182E-4	6	32	912	41585	over
GO:0006032	chitin catabolic process	0.00211451	0.0549938	1.52182E-4	6	32	912	41585	over
GO:0006030	chitin metabolic process	0.00211451	0.0549938	1.52182E-4	6	32	912	41585	over
GO:0006040	amino sugar metabolic process	0.00211451	0.0549938	1.52182E-4	6	32	912	41585	over

GO:0046348	amino sugar catabolic process	0.00211451	0.0549938	1.52182E-4	6	32	912	41585	over
GO:0006044	N-acetylglucosamine metabolic process	0.00211451	0.0549938	1.52182E-4	6	32	912	41585	over
GO:0016681	oxidoreductase activity, acting on diphenols and related substances as donors, cytochrome as acceptor	0.00214855	0.0568677	1.61475E-4	8	64	910	41553	over
GO:0008121	ubiquinol-cytochrome-c reductase activity	0.00214855	0.0568677	1.61475E-4	8	64	910	41553	over
GO:0046914	transition metal ion binding	0.00215532	0.0575497	1.64743E-4	90	2742	828	38875	over
GO:0016998	cell wall catabolic process	0.00225321	0.0606126	1.75642E-4	7	48	911	41569	over
GO:0004568	chitinase activity	0.0022883	0.0620638	1.76587E-4	6	33	912	41584	over
GO:0030151	molybdenum ion binding	0.00241465	0.0659402	1.81539E-4	4	10	914	41607	over
GO:0009825	multidimensional cell growth	0.00285549	0.0781592	2.02369E-4	10	105	908	41512	over
GO:0007018	microtubule-based movement	0.00296408	0.0816886	2.09854E-4	17	272	901	41345	over
GO:0007017	microtubule-based process	0.00298612	0.0829553	2.16922E-4	20	354	898	41263	over
GO:0044459	plasma membrane part	0.00303024	0.0848214	2.32528E-4	20	356	898	41261	over
GO:0008471	laccase activity	0.00303425	0.0856225	2.34615E-4	6	35	912	41582	over
GO:0003824	catalytic activity	0.00390167	0.109594	3.09615E-4	451	18058	467	23559	over
GO:0019252	starch biosynthetic process	0.00458737	0.128574	3.47384E-4	9	92	909	41525	over
GO:0009414	response to water deprivation	0.00489296	0.137583	3.87586E-4	21	400	897	41217	over

GO:0019299	rhamnose metabolic process	0.00496345	0.144743	4.0992E-4	4	13	914	41604	over
GO:0019305	dTDP-rhamnose biosynthetic process	0.00496345	0.144743	4.0992E-4	4	13	914	41604	over
GO:0042631	cellular response to water deprivation	0.00496345	0.144743	4.0992E-4	4	13	914	41604	over
GO:0008831	dTDP-4-dehydrorhamnose reductase activity	0.00496345	0.144743	4.0992E-4	4	13	914	41604	over
GO:0046383	dTDP-rhamnose metabolic process	0.00496345	0.144743	4.0992E-4	4	13	914	41604	over
GO:0006725	aromatic compound metabolic process	0.00529975	0.154877	4.4439E-4	31	712	887	40905	over
GO:0030705	cytoskeleton-dependent intracellular transport	0.00633456	0.183488	4.88648E-4	17	294	901	41323	over
GO:0019200	carbohydrate kinase activity	0.00652454	0.18976	5.10113E-4	10	119	908	41498	over
GO:0010273	detoxification of copper ion	0.00694587	0.202082	5.17418E-4	3	5	915	41612	over
GO:0030258	lipid modification	0.00704746	0.20751	5.18065E-4	4	14	914	41603	over
GO:0030259	lipid glycosylation	0.00704746	0.20751	5.18065E-4	4	14	914	41603	over
GO:0006810	transport	0.00711481	0.210675	5.46519E-4	170	6035	748	35582	over
GO:0043167	ion binding	0.00717554	0.21368	5.62812E-4	139	4780	779	36837	over
GO:0009501	amyloplast	0.00741639	0.221444	5.94096E-4	8	79	910	41538	over
GO:0009733	response to auxin stimulus	0.00743565	0.223395	6.08773E-4	30	694	888	40923	over
GO:0051234	establishment of localization	0.00754704	0.22779	6.33891E-4	170	6055	748	35562	over

GO:0000156	two-component response regulator activity	0.00829088	0.248775	7.03487E-4	6	44	912	41573	over
GO:0005982	starch metabolic process	0.00831479	0.250954	7.28125E-4	10	125	908	41492	over
GO:0051179	localization	0.00836018	0.253696	7.55429E-4	170	6079	748	35538	over
GO:0047216	inositol 3-alpha-galactosyltransferase activity	0.00888841	0.268994	7.63672E-4	3	6	915	41611	over
GO:0008422	beta-glucosidase activity	0.0094947	0.28615	7.926E-4	4	16	914	41601	over
GO:0046872	metal ion binding	0.0103925	0.310341	9.60665E-4	133	4604	785	37013	over
GO:0015293	symporter activity	0.0112579	0.333234	0.00105223	14	233	904	41384	over
GO:0009734	auxin mediated signaling pathway	0.0113465	0.33724	0.0010684	12	181	906	41436	over
GO:0009823	cytokinin catabolic process	0.0117083	0.355452	0.00107347	3	7	915	41610	over
GO:0042447	hormone catabolic process	0.0117083	0.355452	0.00107347	3	7	915	41610	over
GO:0004566	beta-glucuronidase activity	0.0117083	0.355452	0.00107347	3	7	915	41610	over
GO:0019139	cytokinin dehydrogenase activity	0.0117083	0.355452	0.00107347	3	7	915	41610	over
GO:0003777	microtubule motor activity	0.0117083	0.357266	0.0010765	9	109	909	41508	over
GO:0005887	integral to plasma membrane	0.0117083	0.357266	0.0010765	9	109	909	41508	over
GO:0000041	transition metal ion transport	0.0117552	0.360286	0.00109431	7	67	911	41550	over
GO:0009805	coumarin biosynthetic process	0.0153135	0.44547	0.00137585	2	1	916	41616	over

GO:0030794	(S)-coclaurine-N-methyltransferase activity	0.0153135	0.44547	0.00137585	2	1	916	41616	over
GO:0009225	nucleotide-sugar metabolic process	0.015752	0.456897	0.00144115	9	114	909	41503	over
GO:0046688	response to copper ion	0.0167088	0.479125	0.00145239	3	8	915	41609	over
GO:0022610	biological adhesion	0.0167088	0.483188	0.0014919	7	71	911	41546	over
GO:0007155	cell adhesion	0.0167088	0.483188	0.0014919	7	71	911	41546	over
GO:0009699	phenylpropanoid biosynthetic process	0.0178514	0.509725	0.0016716	16	302	902	41315	over
GO:0010053	root epidermal cell differentiation	0.0178514	0.510391	0.00168236	8	94	910	41523	over
GO:0004696	glycogen synthase kinase 3 activity	0.0202356	0.55942	0.00190555	3	9	915	41608	over
GO:0005375	copper ion transmembrane transporter activity	0.0202356	0.55942	0.00190555	3	9	915	41608	over
GO:0015980	energy derivation by oxidation of organic compounds	0.0203618	0.56418	0.00199371	17	337	901	41280	over
GO:0030599	pectinesterase activity	0.0203618	0.566108	0.00200323	9	120	909	41497	over
GO:0043169	cation binding	0.0208348	0.576655	0.00210307	108	3697	810	37920	over
GO:0005886	plasma membrane	0.02257	0.608126	0.00231243	32	828	886	40789	over
GO:0005985	sucrose metabolic process	0.0258165	0.659743	0.00261768	7	79	911	41538	over
GO:0005351	sugar:hydrogen ion symporter activity	0.0296894	0.7127	0.0027295	11	177	907	41440	over
GO:0016682	oxidoreductase activity, acting on diphenols and related substances as donors, oxygen as acceptor	0.03002	0.718783	0.0027846	6	59	912	41558	over

GO:0004473	malate dehydrogenase (oxaloacetate-decarboxylating) (NADP+) activity	0.030716	0.729026	0.0029215	4	24	914	41593	over
GO:0009698	phenylpropanoid metabolic process	0.0310027	0.734377	0.0030063	18	382	900	41235	over
GO:0009690	cytokinin metabolic process	0.0320495	0.750045	0.00305293	3	11	915	41606	over
GO:0009413	response to flooding	0.0320495	0.750045	0.00305293	3	11	915	41606	over
GO:0050794	regulation of cellular process	0.032403	0.755828	0.00314195	95	3227	823	38390	over
GO:0009809	lignin biosynthetic process	0.0340078	0.774228	0.0033736	8	106	910	41511	over
GO:0009877	nodulation	0.0352005	0.787593	0.00368208	5	43	913	41574	over
GO:0008865	fructokinase activity	0.0366822	0.804623	0.00375533	3	12	915	41605	over
GO:0030675	Rac GTPase activator activity	0.0366822	0.804623	0.00375533	3	12	915	41605	over
GO:0046524	sucrose-phosphate synthase activity	0.0373939	0.814978	0.00377942	4	26	914	41591	over
GO:0046915	transition metal ion transmembrane transporter activity	0.0373939	0.814978	0.00377942	4	26	914	41591	over
GO:0006857	oligopeptide transport	0.0373939	0.817669	0.00382154	7	85	911	41532	over
GO:0015833	peptide transport	0.0373939	0.817669	0.00382154	7	85	911	41532	over
GO:0009719	response to endogenous stimulus	0.0386522	0.829478	0.0041691	69	2238	849	39379	over
GO:0006658	phosphatidylserine metabolic process	0.0437327	0.870649	0.00445576	2	3	916	41614	over
GO:0046509	1,2-diacylglycerol 3-beta-galactosyltransferase activity	0.0437327	0.870649	0.00445576	2	3	916	41614	over

GO:0006659	phosphatidylserine biosynthetic process	0.0437327	0.870649	0.00445576	2	3	916	41614	over
GO:0051740	ethylene binding	0.0437327	0.870649	0.00445576	2	3	916	41614	over
GO:0003872	6-phosphofructokinase activity	0.0470897	0.890743	0.00479349	4	28	914	41589	over
GO:0012501	programmed cell death	0.0472404	0.89279	0.00487237	11	192	907	41425	over
GO:0005874	microtubule	0.0476739	0.896211	0.00510961	15	310	903	41307	over
